# Synthesis of Known and Previously Inaccessible Poly(pyrazolyl)Borates under Mild Conditions

**DOI:** 10.1021/acs.joc.3c00761

**Published:** 2023-06-22

**Authors:** María M. Melero, Zuzanna Kłosek, Carmen Ramírez de Arellano, Andrea Olmos

**Affiliations:** Departamento de Química Orgánica, Universidad de Valencia, Av. Vicente Andrés Estellés S/N. 46100 Burjassot, Valencia, Spain

## Abstract

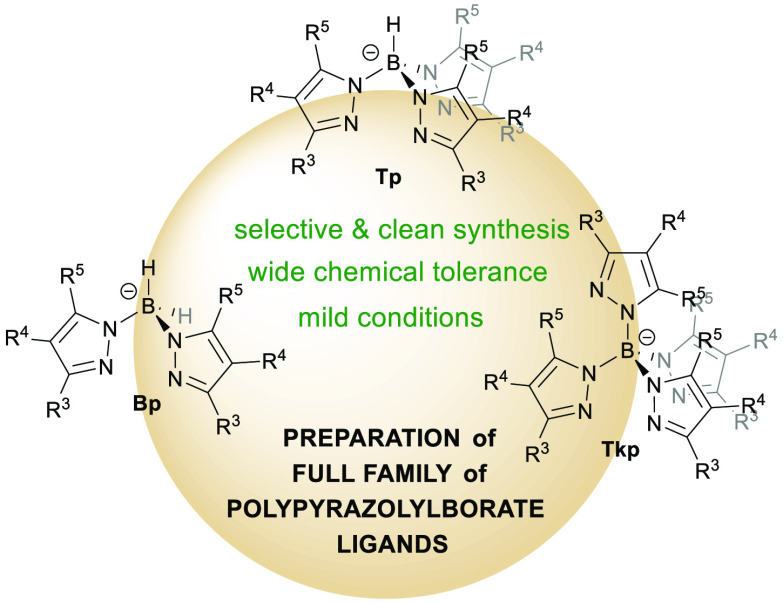

Poly(pyrazolyl)borate ligands have been obtained through the reaction of highly reactive haloboranes with in situ formed pyrazolides under very mild conditions. This versatile synthetic method allows the selective synthesis of bis-, tris-, or tetrakis(pyrazolyl)borates. Furthermore, the method is compatible with the use of functional groups on the heterocyclic moieties of the poly(pyrazolyl)borates that were not accessible to date. Strongly encumbered sodium and thallium(I) poly(pyrazolyl)borates with a reduced donating ability have been obtained for the first time.

## Introduction

Poly(pyrazolyl)borate ligands were described for the first time by Trofimenko during the 1960s.^1^ The first generation of these scorpionate ligands provided sandwich complexes of metals presenting octahedral coordination.^2^ The introduction of bulky substituents on position 3 of the heterocyclic rings was considered the second generation and allowed the isolation of metal complexes bearing only one ligand^3^ and therefore vacant coordination positions at the metal opening the door to develop catalytic processes. Since then, poly(pyrazolyl)borates of almost any transition metal have been prepared^4^ and used as enzymatic models,^5^ for the development of new materials,^6^ and as power catalysts in reactions as carbene or nitrene C–H insertion, polymerization or carbonyl derivatizations.^7^ The success of this family of ligands resides in the possibility of fine-tuning the electronic and steric properties of the metal complexes through the introduction of appropriate groups on the heterocyclic rings. However, despite the more than 4200 crystal structures of this type of complexes that have been described,^8^ the simultaneous pyrazole decoration with bulky and electron-withdrawing substituents has not been possible to date.

**Scheme 1:**
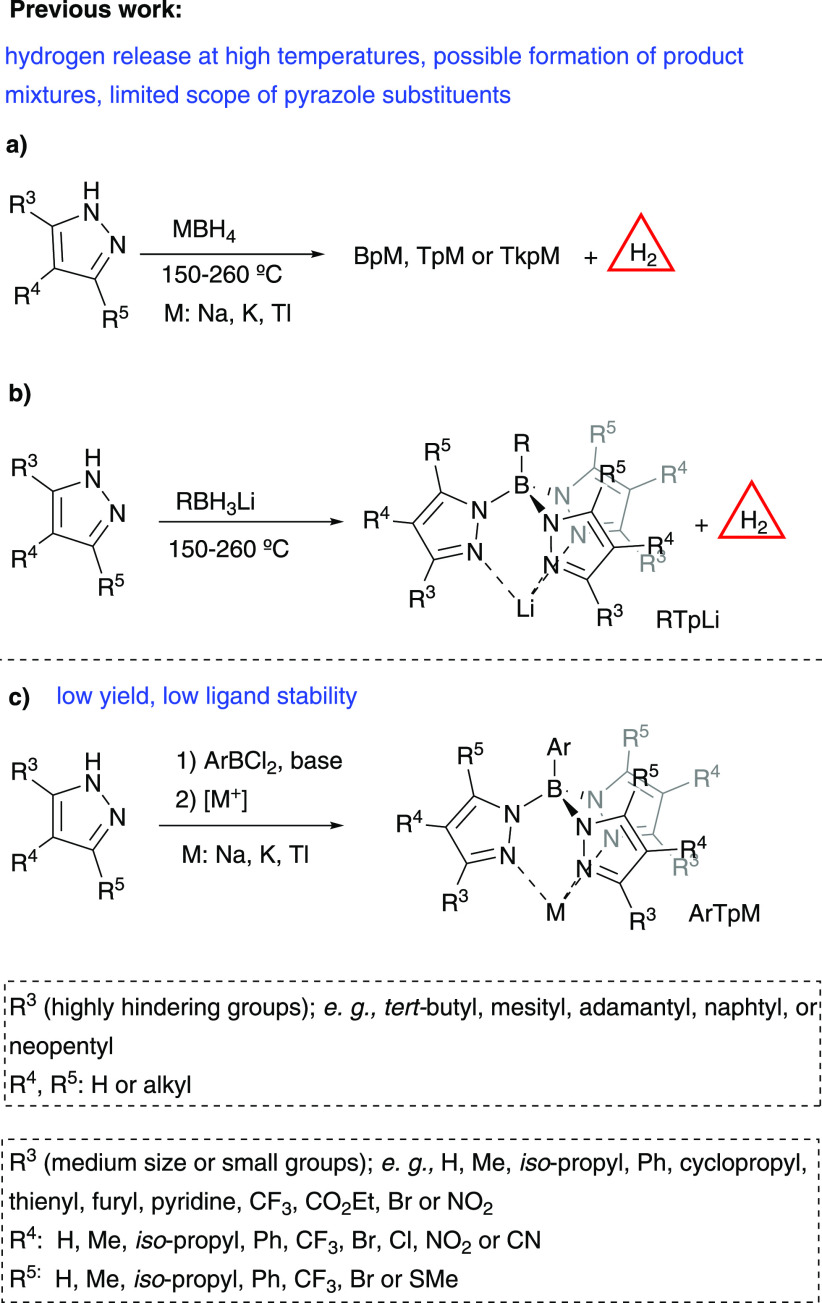
Previous Syntheses of Poly(pyrazolyl)Borate Ligands

The most common route to prepare poly(pyrazolyl)borates is the reaction of a high excess of the desired pyrazole derivative with a metal borohydride in the absence of solvent (Scheme 1a).^9^ This transformation presents drawbacks: (i) difficult control of the reaction stoichiometry with possible formation of mixtures of dihydrobis(pyrazolyl)borates (Bp^x^), hydrotris(pyrazolyl)borates (Tp^x^) and tetrakis(pyrazolyl)borates (Tkp^x^), (ii) hazardous evolution of hydrogen gas under high temperature conditions,^10^ (iii) limited functional group scope due to their sensibility under reductant conditions and (iv) pyrazoles containing simultaneously electron-withdrawing and bulky substituents do not react under these conditions due to reduced nucleophilicity and higher steric hindrance. The access to Tp^x^ ligands presenting these characteristics could widen the catalytic applications of their metal complexes due to the increased electrophilicity,^11^ easier reduction,^12^ and higher stability of low oxidation states^13^ of the corresponding metal centers.

The third generation of tris(pyrazolyl)borates appeared with the substitution of the hydrogen atom with an alkyl or an aryl moiety. Alkyltris(pyrazolyl)borates were prepared from lithium alkylborohydride derivatives,^14^ very flammable reagents, in a reaction with the same disadvantages related before (Scheme 1b). In this case, addition of a Lewis acid allowed the use of milder conditions but did not avoid the use of hazardous alkylborohydride reagents.^15^ Aryldihaloboranes were used as an alternative to borohydride compounds for the preparation of aryltris(pyrazolyl)borates (Scheme 1c).^16^ Although milder reaction conditions were used, this procedure was limited by the poor yield achieved in most cases, the low availability of aryldichloroborane derivatives,^17^ and the lower stability of the ligands, associated with the lability of the B–N bonds increased by the introduction of an aryl moiety on the boron atom. Good yield was obtained for (Ipc)BCl_2_ with nonsubstituted sodium pyrazolide.^17^

**Scheme 2:**
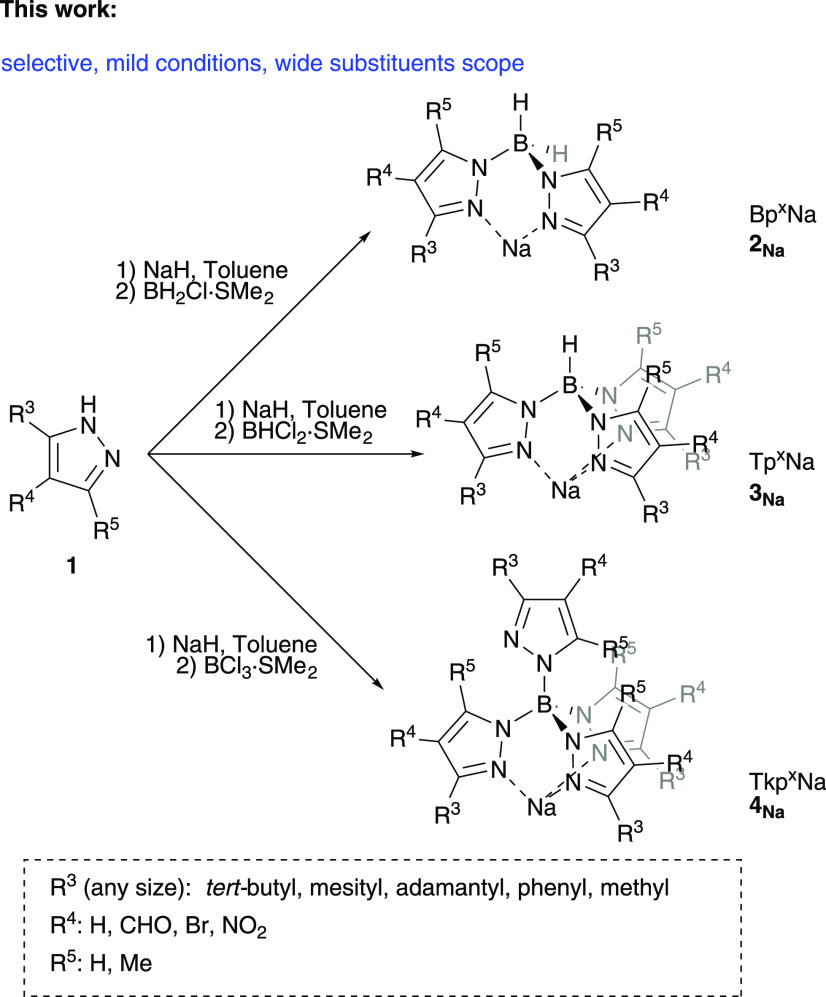
New Route to Poly(pyrazoly)Borate Ligands

Here we present a new practical and direct methodology for the preparation of poly(pyrazolyl)borate ligands under very mild conditions, with wide applicability that yields exclusively the desired poly(pyrazolyl)borate derivative in good to excellent yields (Scheme 2).

## Results and Discussion

### Synthesis of Tris(pyrazolyl) Borate Ligands (**3**)

**Scheme 3:**
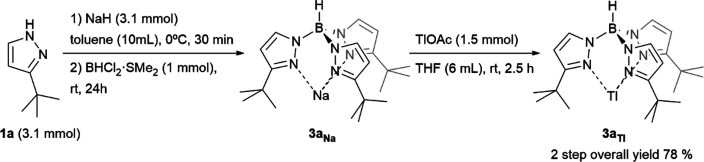
Optimized Conditions for **3a**_**Tl**_

First, we focused on more extended thalium(I) tris(pyrazolyl)borates (**3**_**Tl**_), the usual entry for metal exchange.^4a^ A dichloroborane dimethylsulfide complex (BHCl_2_·SMe_2_) was chosen as the highly reactive boron source and 3-*tert*-butylpyrazole (**1a**) as the standard azaheterocycle (Scheme 3). A base must be added to avoid reaction inhibition through protonation of the remaining pyrazole as the reaction proceeds (see SI). A smooth reaction takes place between *in situ* formed sodium pyrazolide and the chosen boron source with the formation of the sodium salt of the desired hydrotris(3-*tert*-butylpyrazol-1-yl)borate (**3a**_**Na**_) just using the *ca*. stoichiometric amounts of all reagents on toluene at room temperature for 24 h. Conditions optimization for tris(pyrazolyl)borate ligands synthesis has been performed and is specified in the Supporting Information (see SI Section 3). Thallium salt **3a**_**Tl**_ was prepared *in situ* through standard procedures to facilitate product purification.^14a^

**Figure 1:**
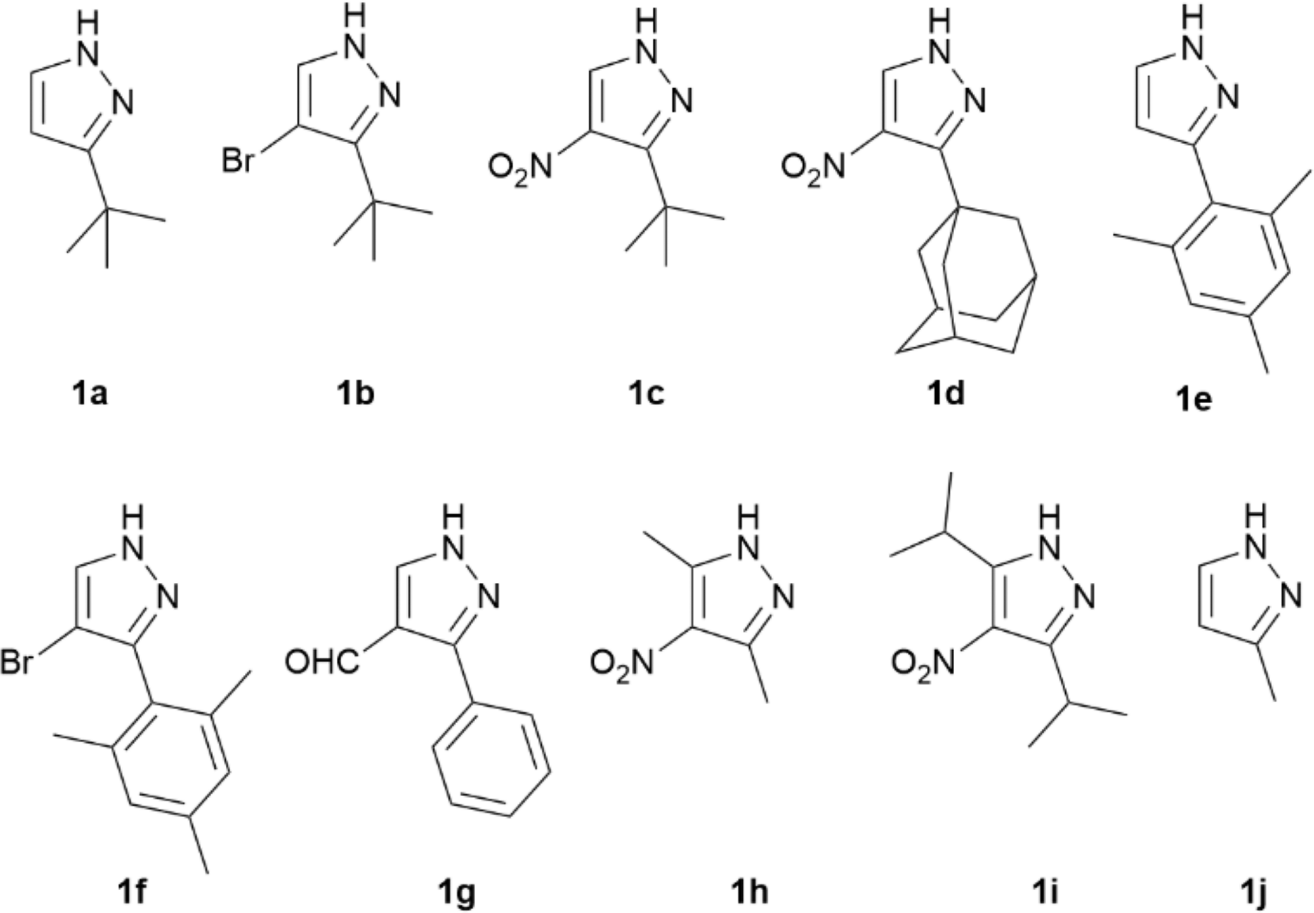
Pyrazole derivatives used in the preparation of thallium(I) hydrotris(pyrazolyl) borates.

These optimized conditions were used in the preparation of thallium hydrotris(pyrazolyl)borate complexes using the selection of pyrazole derivatives, as shown in Figure 1. Table 1 summarizes the results obtained for the preparation of Tp^x^Tl (**3a**–**j**_**Tl**_) using the optimized reaction conditions. An excellent 90% yield was obtained for complex **3b**_**Tl**_ bearing the encumbering *tert*-butyl group at position 3 and a bromine atom at position 4 of the heterocycle. This method allows for the first time the preparation of boron scorpionate ligands with a strongly electron-withdrawing nitro group in the presence of a bulky substituent such as *tert*-butyl. Ligand **3c**_**Tl**_ was prepared in a satisfactory 93% yield. Introduction of the bulkier adamantyl group at position 3 (**3d**_**Tl**_) did not negatively affect the product yield. All attempts to prepare **3b**_**Tl**_, **3c**_**Tl**_ and **3d**_**Tl**_ presenting electron-withdrawing groups and highly hindering *tert*-butyl or adamantyl substituents through literature methods were unsuccessful.^3a,3b,9a,9b^ The preparation of Tp^x^Tl bearing a mesityl group on position 3 also proceeded smoothly in good yields with a hydrogen (**3e**_**Tl**_) or a bromine (**3f**_**Tl**_) atom on position 4 of the pyrazole ring.

**Table 1:** Yield and Regioselectivity of Thallium(I) Hydrotris(pyrazolyl) Borate Complexes (**3a**–**j**_**Tl**_)^a^

Pyrazole	Tp^x^Tl	R^3^	R^4^	R^5^	Yield (%)^b^
**1a**	**3a**_**Tl**_	*t*Bu	H	H	78, 92^c^
**1b**	**3b**_**Tl**_	*t*Bu	Br	H	96
**1c**	**3c**_**Tl**_	*t*Bu	NO_2_	H	93
**1d**	**3d**_**Tl**_	Ad	NO_2_	H	51
**1e**	**3e**_**Tl**_	Ms	H	H	81
**1f**	**3f**_**Tl**_	Ms	Br	H	78
**1g**	**3g**_**Na**_	Ph	CHO	H	79^d^
**1h**	**3h**_**Na**_	Me	NO_2_	Me	86^d^
**1i**	**3i**_**Tl**_	iPr	NO_2_	iPr	<5^e^^,^^f^
**1j**	**3j**_**Tl**_	Me/H	H	Me/H	85

a Reaction conditions: **1a**–**j** (12.1 mmol), NaH (12.1 mmol), toluene (40 mL) 30 min at 0 °C, BHCl_2_·SMe_2_ (4 mmol) 24 h at rt. Evaporation and addition of THF (25 mL), TlOAc (6 mmol), 2 h at rt.

b Isolated yield.

c Isolated yield obtained in a reaction performed on 5 g scale.

d Tp^x^Na complexes were isolated.

e 24 h at 100 °C.

f Conversion into **3i**_**Tl**_ calculated by ^1^H NMR.

We have also explored the compatibility of this new procedure for the synthesis of pyrazolylborates supporting functional groups of special sensibility to reductant environments. We used our standard conditions in the reaction with pyrazole **1g** bearing a sensitive aldehyde substituent at position R^4^. As expected, the reaction proceeded smoothly, and the corresponding sodium complex **3g**_**Na**_ was obtained in 79% yield. Finally, we challenged the scope of the method using as the starting material the trisubstituted pyrazole **1h** containing two methyl groups and one electron-withdrawing nitro group. The expected product **3h**_**Na**_ was obtained in a satisfactory 86% yield. For pyrazoles **1g** and **1h** sodium tris(pyrazolyl)borates were obtained directly, and sodium to thallium exchange was not performed. The introduction of two encumbering isopropyl groups hinders the formation of **3i**_**Tl**_, and only traces of the expected product could be detected even at higher temperatures.

It is noticeable the complete regioselectivity of the reaction with the boron bonded to the nitrogen atom placed farther from the encumbering group for pyrazoles **1a**–**g** (R^3^ in Table 1), thus providing good control of the potential catalytic pocket for hindered pyrazoles. This is a significant advantage over previous methods that often provide regioisomeric mixtures.^3c^ The regioselectivity achieved is dependent on the size of the substituent at position 3. For methyl-substituted pyrazole **1j**, the formation of the four possible regiosiomers was observed. The synthetic usefulness of this procedure was demonstrated by the synthesis of **3a**_**Tl**_ on a 5 g scale that provided a remarkable 92% yield.

### Synthesis of Bis(pyrazolyl)Borate Ligands (**2**)

We extended this new methodology to the preparation of less explored thallium dihydrobis(pyrazolyl)borates (**2**_**Tl**_),^16,18^ difficult to isolate as pure materials.^3b,9a,19^ The results obtained using a commercially available chloroborane dimethylsulfide complex (BH_2_Cl·SMe_2_) for a selection of thallium(I) Bp^x^ complexes are shown in Table 2. Optimized reaction conditions for **3_Tl_** were used, and pyrazole and base equivalents were adjusted to ca. ideal stoichiometric amounts with good results. Pyrazoles **1a**–**c** bearing a highly encumbering *tert*-butyl group cleanly produced the expected **2a**–**c**_**Tl**_ in good to excellent isolated yields. In these cases, the isolation of compounds **2a**–**c**_**Tl**_ is greatly facilitated by our selection of the boron source that allows full control of the 2:1 stoichiometry, thus avoiding contamination with other pyrazolylborates. However, the use of the standard conditions for **2e**–**f** yielded a complex reaction mixture due to the formation of the desired **2e**–**f**_**Tl**_ besides the two corresponding pyrazaboles with two bridgehead boron atoms (see SI).^1a,20^ The use of an excess of pyrazolide for **1f** prevented the formation of the undesired product and yielded exclusively the formation of **2f**_**Tl**_. Unreacted pyrazole can be easily removed from the reaction crude through Et_2_O washing before sodium to thallium exchange.

**Table 2:**
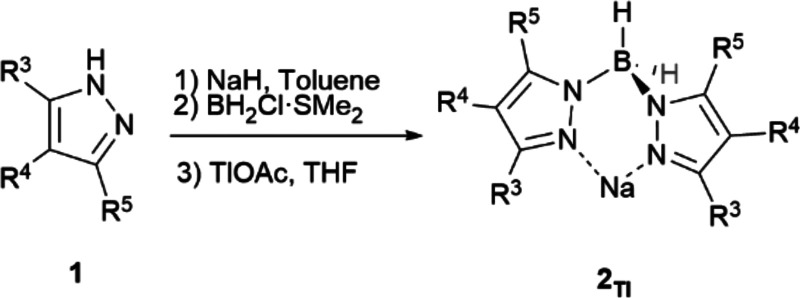
Yield and Regioselectivity of Thallium(I) Dihydrobis(pyrazolyl)borate Complexes (**2a**–**f**_**Tl**_)^a^

Pyrazole	R^3^	R^4^	R^5^	Bp^x^Tl (**2_Tl_**) Yield (%)^b^
**1a**	*t*Bu	H	H	90
**1b**	*t*Bu	Br	H	87
**1c**	*t*Bu	NO_2_	H	92
**1f**	Ms	Br	H	76^c^

a Reaction conditions: **1a**–**c** (10.1 mmol), NaH (10.1 mmol), toluene (50 mL) 30 min at 0 °C, BH_2_Cl·SMe_2_ (5 mmol) 24 h at rt. Evaporation and addition of THF (30 mL), TlOAc (7.5 mmol), 2 h at rt.

b Isolated yield.

c 20.1 mmol of **1f** and NaH (4.02 equiv) were used.

### Synthesis of Tetrakis(pyrazolyl)Borate Ligands (**4**)

Further, we extended our procedure for the preparation of sterically challenging and rare tetrakis(pyrazolyl)borates.^21^ Standard conditions were applied to the synthesis of Tkp^x^ ligands increasing pyrazole and base amounts to almost stoichiometric 4.1 equiv to ensure complete conversion of BCl_3_·SMe_2_ into the desired tetrakis(pyrazolyl)borate. The scarce examples described of these ligands are almost restricted to those presenting methyl or hydrogen groups on positions 3 and/or 5 of the pyrazole rings due to the difficulty associated with the thermal introduction of the fourth heterocycle.^3b,19c,19g,22^Table 3 shows the remarkable yields obtained for a variety of Tkp^x^Na (**4a**–**f**_**Na**_) with highly encumbering substituents. Alkaline salts of these ligands are a common entrance to other metal complexes through metal exchange.^22^

**Table 3:**
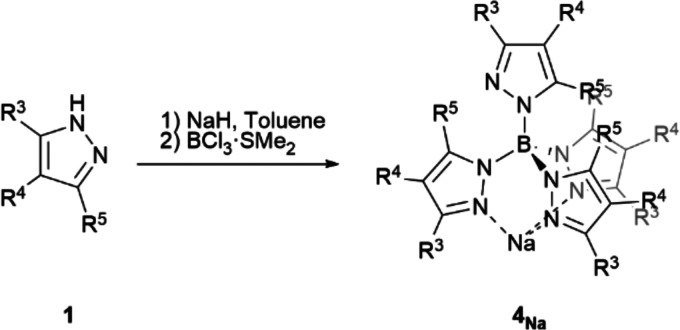
Yield and Regioselectivity of Sodium Tetrakis(pyrazolyl)Borates (**4a**–**f**_**Na**_)^a^

Pyrazole	R^3^	R^4^	R^5^	Tkp^x^Na (**4_Na_**) Yield (%)^b^
**1a**	*t*Bu	H	H	73
**1b**	*t*Bu	Br	H	64
**1c**	*t*Bu	NO_2_	H	91
**1f**	Ms	Br	H	93

a Reaction conditions: **1a**–**f** (4.1 mmol), NaH (4.1 mmol), toluene (15 mL) 30 min at 0 °C, BCl_3_·SMe_2_ (1 mmol) 24 h at rt.

b Isolated yield.

### Molecular Structure Determination

**Figure 2:**
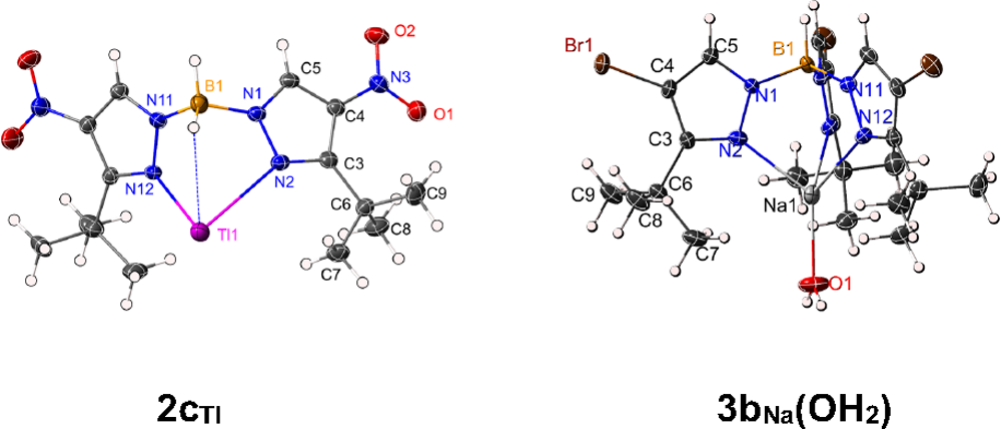
X-ray structures of series **2c**_**Tl**_ and **3b**_**Na**_**(OH**_**2**_**)**.

Structures of **2c**_**Tl**_, **3b**_**Na**_**(OH**_**2**_**)**, and **4f**_**Tl**_ were determined by single crystal X-ray diffraction (Figure 2).

## Conclusions

In conclusion, we have developed a useful and versatile new synthetic procedure for the selective preparation of Bp^x^, Tp^x^ and Tkp^x^ ligands in a complete selective way under safe and mild conditions. The wide scope of this reaction allows for the first time access to compounds bearing labile functional groups, such as nitro or aldehyde, and including highly hindering and electron-withdrawing substituents simultaneously. Remarkably, a significant additional advantage of the method compared to described procedures is the use of ca. stoichiometric amounts of starting pyrazole derivatives. This method has allowed for the first time the preparation of a challenging new set of poly(pyrazolyl)borate ligands with excellent yields and complete regioselectivity.

## Data Availability

The data underlying this study are available in the published article and its Supporting Information.

## Supporting Information Available

Detailed synthetic procedures, characterization of all products and X-ray structural discussion (PDF)
FAIR data, including the primary NMR FID files, for compounds **1a**, **1b**, **1c**, **1d**, **1e**, **1f**, **1g**, **1h**, **1i**, **2a**_**Tl**_, **2b**_**Tl**_, **2c**_**Tl**_, **2f**_**Tl**_, **3a**_**Tl**_, **3b**_**Tl**_, **3c**_**Tl**_, **3d**_**Tl**_, **3e**_**Tl**_, **3f**_**Tl**_, **3g**_**Na**_, **3h**_**Na**_, **4a**_**Na**_, **4b**_**Na**_, **4c**_**Na**_, **4f**_**Na**_, **4f**_**Tl**_, pyrazaboles from **1e** and **1f** (ZIP)
