# Supplementary material for: Synthesis of Known and Previously Inaccessible Poly(pyrazolyl)Borates under Mild Conditions
Source: J Org Chem. 2023 Jun 22;88(13):9130–5. doi: 10.1021/acs.joc.3c00761 (PMC10337037; doi:10.1021/acs.joc.3c00761)
Supplement: Supplementary file 1 — jo3c00761_si_001.pdf [file jo3c00761_si_001.pdf]

Synthesis of known and previously inaccessible poly(pyrazolyl)borates under mild conditions.

María M. Melero,[a] Zuzanna Kłosek,[a] Carmen Ramírez de Arellano*[a] and Andrea Olmos*[a]

[a]Departamento de Química Orgánica, Universidad de Valencia. Av. Vicente Andrés Estellés S/N. 46100 Burjassot (SPAIN).

Corresponding authors: carmen.ramirezdearellano@uv.es, andrea.olmos@uv.es

Supporting Information

Content

1. General information	SI-3
2. Preparation of pyrazole derivatives (**1**)	SI-4
3. Optimization of $\mathbf{3a_{Na}}$ formation	SI-9
4. General synthesis of thallium (I) and sodium hydrotris(pyrazolyl)borates ($\mathbf{3a_{Tl}}$, $\mathbf{3b_{Tl}}$, $\mathbf{3c_{Tl}}$, $\mathbf{3d_{Tl}}$, $\mathbf{3e_{Tl}}$, $\mathbf{3f_{Tl}}$, $\mathbf{3g_{Na}}$, and $\mathbf{3h_{Na}}$)	SI-10
5. General synthesis of thallium (I) dihydrobis(pyrazolyl)borates ($\mathbf{2a\text{-}f_{Tl}}$)	SI-12
6. Characterization of pyrazabole derivatives from **1e-f**	SI-14
7. General synthesis of sodium tetrakis(pyrazolyl)borates ($\mathbf{4_{Na}}$)	SI-14
8. NMR, IR and HRMS spectra	SI-16
9. Single crystal X ray structure determination for $\mathbf{2c_{Tl}}$, $\mathbf{3b_{Na}(OH_2)}$, and $\mathbf{4f_{Tl}}$	SI-69
10. References (S1-S15)	SI-74

1. General information.

Haloboranes are reagents sensitive to hydrolysis. All transformations implying these reagents have been performed using common Schlenk techniques or in a glove box. Dichloroborane dimethylsulfide complex is obtained from Merck. A careful manipulation of this reagent is advised since while recently opened commercial reagent presents < 4 % of $BCl_3{\cdot}SMe_2$ and < 4 % of $BH_2Cl{\cdot}SMe_2$, as checked by ^{1}H and ^{11}B-NMR, when handling the reagent, small amounts of auxiliary SMe_2 are lost through evaporation and a very slow decomposition through H/Cl exchange is observed. After a period of three months *ca*. 10 % of $BCl_3{\cdot}SMe_2$ and 10 % of $BH_2Cl{\cdot}SMe_2$ was quantified. This contamination must be inhibited to avoid the loss of selectivity on the borate formation reaction. This decomposition can be prevented storing the reagent in the fridge and adding small amounts of anhydrous SMe_2 periodically. $BH_2Cl{\cdot}SMe_2$ reagent, also purchased from Merck, presented the same exchange process, and received identical treatment. Dimethylsulfide has an unpleasant odor and manipulation of its derivatives should be manipulated on a fuming hood. Solutions containing SMe_2 should be treated with sodium hypochlorite before their disposal. CAUTION! Thallium salts and their derivatives are highly toxic. All thallium containing compounds must be handled with care and disposed conveniently. Pyrazole **1j** was obtained from VWR and used as received. Anhydrous toluene was dried with sodium, anhydrous Et_2O and THF were dried with sodium using benzophenone as indicator prior to use. DMF was achieved anhydrous and used as received. Poly(pyrazolyl)borates are stable compounds against moisture. Deuterated chloroform, deuterated acetone and deuterated dimethylsulfoxide have been used as received. Siligel 60 (40-63 mm) from Merck was used for column chromatography purification. NMR analysis have been performed in a Bruker AvanceIII 300, a Bruker AV400 or a Bruker Neo500 spectrometer. NMR data have been processed using MestReNova™ and are expressed in ppm. Residual signals of deuterated solvents have been used as internal reference ($CHCl_3$ at 7.26 ppm in ^{1}H-NMR and $CDCl_3$ 77.2 ppm in ^{13}C-NMR, $CHDCl_2$ at 5.32 ppm in ^{1}H-NMR and CD_2Cl_2 53.8 ppm in ^{13}C-NMR, DMSO-d_5 at 2.50 ppm and DMSO-d_6 39.5 ppm in ^{1}H-NMR and ^{13}C-NMR respectively, acetone-d_5 at 2.05 ppm and acetone-d_6 at 29.8 ppm in ^{1}H-NMR and ^{13}C-NMR respectively, C_6HD_5 at 7.16 pp in 1H-NMR and C6D6 at 128.1 ppm in ^{13}C-NMR). Internal equipment calibration was used for ^{11}B-NMR. IR spectra have been recorded on a Thermo Scientific Nicolet iS10 and processed with Omnic™. IR frequencies have been rounded to 1 cm^{-1}. HRMS (+ESI) analysis have been performed in AB SCIEX TripleTOF™ 5600 LC/MS/MS System and data have been processed using PeakView™. Acidic methanolic conditions used during HRMS analysis yielded the identification of protonated borates as $[Tp^{x}H+H]^{+}$, $[Bp^{x}H+H]^{+}$ or $[Tkp^{x}H+H]^{+}$. MALDI-QTOF analyses were performed in a TIMS-TOFF Flex (Bruker) in MALDI operation, in reflector positive mode at 200-3500 m/z rang and a laser intensity of 40 % The analysis was performed in the proteomics facility of SCSIE University of Valencia. Elemental analyses have been performed in a Thermofisher Flashmart Eager 200. X ray single crystal structures have been measured on Oxford Diffraction Supernova or D8 Venture Diffractometers. The structures were solved through dual space methods using SHELXT[S1] and SHELXL-2018[S2].

2. Preparation of pyrazole derivatives (1)

3-*tert*-butyl-1-*H*-pyrazole (1a).

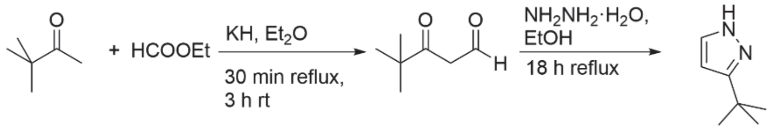

1a

3-tert-butyl-1-H-pyrazole (**1a**) was prepared through a modified procedure of a described method.[S3] On a dry 250 two-necked round bottom flask provided with a condenser and an addition funnel a mixture of pinacolone (6.25 mL, 50 mmol, 1 eq.) and ethyl formate (8.1 mL,100 mmol, 2 eq.) was slowly added over a suspension of potassium hydride (2.4 g, 60 mmol, 1.2 eq.), previously washed with *n*-hexane, on 25 mL of anhydrous diethyl ether under argon. The mixture was gently refluxed for 30 minutes in an oil bath and stirred a room temperature for additional 3 hours. The reaction crude was carefully hydrolyzed with 2 mL of isopropyl ether and diluted with 150 mL of water. The aqueous was washed with diethyl ether (2 x 30 mL) and acidified with concentrated aqueous hydrochloric acid until pH 1-2. The product was then extracted with diethyl ether (3x30 mL). The acidic extracts were combined, washed with brine, dried over anhydrous $MgSO_4$ and concentrated to provide 5.24 g (40.9 mmol, 82 % yield) of crude 4,4-dimethyl-3-oxopentanal as a yellowish oil. The crude product was directly solved in 150 mL of ethanol and monohydrated hydrazine as added (2.0 mL, 40.9 mmol, 1 eq.). The yellow solution was refluxed for 18 hours. Once at room temperature, the solvent was evaporated and the crude product purified through distillation on a kugelrohr oven (bp: 90 ^{o}C, 0.3 mbar). The product solidified upon cooling down after distillation. Pure 3-*tert*-butyl-1-*H*-pyrazole was obtained as a colorless solid (4.2 g, 34.76 mmol, 85 % yield). Colorless crystalline solid; mp:78 ^{o}C (described mp: 83 ^{o}C). Its NMR data were consistent with literature values.[S3]

^{1}H NMR ($CDCl_3$, 300 MHz, 25 ^{o}C): δ 7.48 (d, 1H, J = 2.0 Hz), 6.11 (d, 1H, J = 2.0 Hz), 1.34 (s, 9H); ^{13}C{^{1}H} NMR ($CDCl_3$, 75 MHz, 25 ^{o}C): δ 156.7, 135.5, 101.4, 31.4, 30.6.

4-bromo-3-*tert*-butyl-1-*H*-pyrazole (1b).

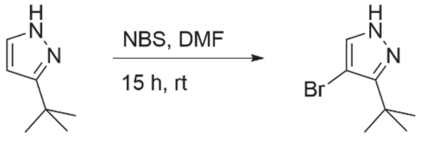

1a 1b

4-bromo-3-tert-butyl-1-H-pyrazole (**1b**) was prepared through a modified procedure of a described method.[S4] To a solution of **1a** (6.21 g, 50 mmol, 1 eq.) in 50 mL of *N,N*-dimethylformamide was added *N*-bromosuccinimide at once (9.07 g, 51 mmol, 1.02 eq.). The solution was stirred for 15 h at room temperature and diluted with 200 mL of water. The product was extracted with ethyl acetate (4 x 40 mL). The organic extracts were combined, washed with water (3x 40 mL), washed with brine, dried over anhydrous $MgSO_4$, and concentrated. Crude **1b** was purified through a short column chromatography using *n*-hexane:AcOEt 5:1 as eluent. Pure 4-bromo-3-*tert*-butyl-1-*H*-pyrazole (**1b**) was obtained as a white solid in 86 % yield (8.8 g, 42.99 mmol). mp:128 ^{o}C (described mp:122-123 ^{o}C). Its NMR data were consistent with literature values.[S5]

^{1}H NMR ($CDCl_3$, 500 MHz, 25 ^{o}C): δ 9.24 (bs, 1H), 7.48 (s, 1H), 1.43 (s, 9H); ^{13}C{^{1}H} NMR ($CDCl_3$, 125 MHz, 25 ^{o}C): δ 150.3, 139.1, 90.9, 32.4, 28.7.

3-*tert*-butyl-4-nitro-1-*H*-pyrazole (1c).

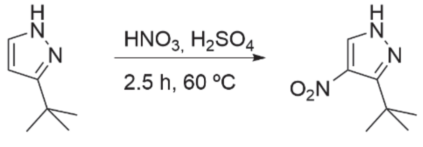

1a **1c**

3-tert-butyl-4-nitro-1-H-pyrazole (**1c**) was prepared through a described method.[S6] 3-*tert*-butylpyrazole (**1a**, 1.24 g, 10 mmol, 1 eq.) was solved in 6 mL of cold concentrated sulfuric acid. The solution was warmed to 60 °C in an oil bath and 65 % aqueous nitric acid (1.1 mL, 11 mmol, 1.1 eq.) was added. The mixture was maintained at that temperature for 2.5 hours. After cooling to room temperature, the mixture was poured onto 60 g of iced water. The aqueous phase was thoroughly extracted with ethyl acetate (5 x 40 mL). Organic phases were combined, washed with brine, dried over anhydrous $MgSO_4$, and concentrated. Crude **1c** was purified through short column chromatography using *n*-hexane:AcOEt 2:1 as eluent. 3-*tert*-butyl-4-nitro-1-*H*-pyrazole (**1c**) was obtained in 89 % yield as a pale-yellow solid (1.35 g, 8.9 mmol). mp:115-118 °C (described mp:118-119 °C) Its NMR data were consistent with literature values.[S6]
^1H NMR ($CDCl_3$, 300 MHz, 25 °C): δ 8.29 (s, 1H), 1.52 (s, 9H); $^{13}C\{^1H\}$ NMR ($CDCl_3$, 75 MHz, 25 °C): δ 151.4, 138.5, 133.3, 33.3, 27.8.

3-adamantyl-4-nitro-1-*H*-pyrazole (1d).

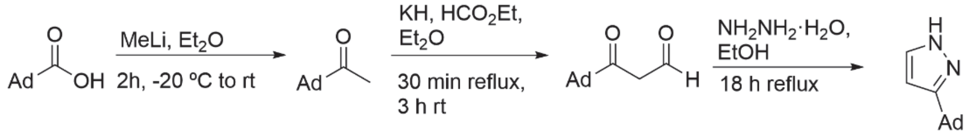

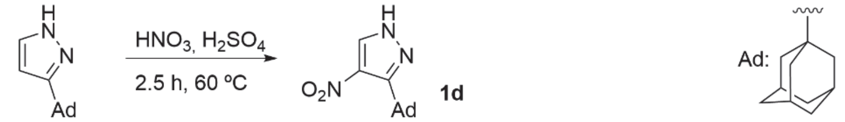

3-adamantyl-4-nitro-1-H-pyrazole (**1d**). To a solution of 1-adamantanecarboxylic acid (1.8 g, 10 mmol, 1 eq.) in 10 mL anhydrous diethyl ether at -20 °C was added under argon 21 mL of a 1M solution of methyllithium in diethyl ether (21 mmol, 2.1 eq.). After 10 min stirring at that temperature, the reaction mixture is allowed to reach room temperature and stirred for additional 2 hours. The solution was carefully hydrolyzed with water (20 mL) and the phases separated. The aqueous phase was additionally extracted with diethyl ether (2 x 20 mL). Crude adamantyl methyl ketone was obtained in 1.73 g (9.7 mmol, 97 % yield) as a white solid and used without purification.
A mixture of adamantyl methyl ketone (2.7 g, 15 mmol, 1 eq.) and ethyl formate (2.4 mL, 30 mmol, 2 eq.) was slowly added over a suspension of potassium hydride (0.7 g, 18 mmol, 1.2 eq.), previously washed with *n*-hexane, on 10 mL of anhydrous diethyl ether under argon. The mixture was gently refluxed for 30 minutes in an oil bath and stirred at room temperature for additional 1.5 hours. The reaction crude was carefully hydrolyzed with 1 mL of isopropyl ether and diluted with 50 mL of water. The aqueous phase was washed with diethyl ether (2 x 15 mL) and acidified with concentrated aqueous hydrochloric acid until pH 1-2. The product was the extracted with diethyl ether (3 x 20 mL). The acidic extracts were combined, washed with brine, dried over anhydrous $MgSO_4$ and concentrated to provide 1.53 g (7.4 mmol, 49 % yield) of crude 3-adamantyl-3-oxopropanal as a yellowish oil. The crude product was directly solved in

25 mL of ethanol and monohydrated hydrazine as added (0.36 mL, 7.4 mmol, 1 eq.). The yellow solution was refluxed for 4 hours in an oil bath. Once at room temperature, the solvent was evaporated and 1.42 g of crude 3-adamantyl-1-*H*-pyrazole were obtained as a yellowish solid (7 mmol, 94 % yield).
Crude 3-adamantyl-1-*H*-pyrazole (810 mg, 4 mmol, 1 eq.) was solved in 4 mL of cold sulfuric acid. The solution was warmed to 60 °C in an oil bath and 65% aqueous nitric acid (0.45 mL, 4.4 mmol, 1.1 eq.) was added. After 4 h stirring at that temperature the mixture was allowed to cool down and poured onto 40 mL of iced water. Aqueous phase was extracted with AcOEt (5 x 25 mL). The organic phases were combined, washed with brine, dried over anhydrous $MgSO_4$ and concentrated. Crude **1d** was purified through column chromatography using *n*-hexane:AcOEt 2:1 as eluent. Pure 3-adamantyl-4-nitro-1-*H*-pyrazole (**1d**) was obtained in 67 % yield as a pale-yellow solid (662 mg, 2.56 mmol). mp: 152-153 °C.
^{1}H NMR (CD_3OD, 300 MHz, 25 °C): δ 8.31 (s, 1H), 2.32-2.28 (m, 2H), 1.15-2.06 (m, 7H), 1.80-1.68 (m, 6H); ^{13}C{^{1}H} NMR (CD_3OD, 75 MHz, 25 °C): δ 151.5, 137.7, 134.5, 68.8, 47.0, 45.0, 39.7, 38.8, 36.2, 31.8. Anal. Calcd for C13H19N3O3 ($pz^{NO2,Ad}$·H_2O): C, 58.85; H, 7.22; N, 15.84. Found: C, 58.59; H, 7.22; N, 15.84.

3-mesityl-1-*H*-pyrazole (1e).

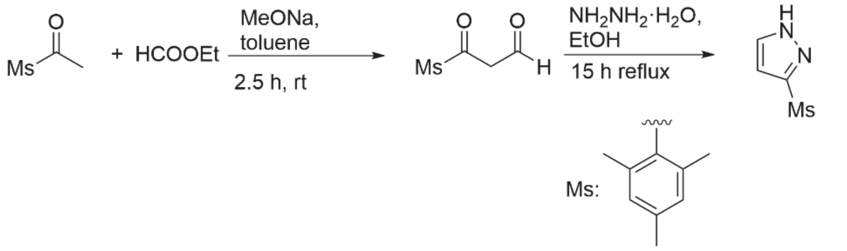

1e

3-Mesityl-1-H-pyrazole (**1e**) was prepared through a modified described procedure.[S7] Over a suspension of sodium methoxide (2.04 g, 38 mmol, 1 eq.) in 65 mL of anhydrous toluene was quickly added under argon the mixture of 2',4',6'-trimethylacetophenone (6.3 mL, 38 mmol, 1 eq.) and ethyl formate (13.6 mL, 169 mmol, 4.4 eq.). A yellow solution was formed and after some minutes it became a white paste. The mixture was stirred for 15 hours at room temperature before dilution with water. Phases were separated and the aqueous phase washed with diethyl ether twice (2 x 25 mL). The aqueous phase was acidified with concentrated hydrochloric acid to pH 1-2 and extracted with diethyl ether (3 x 40 mL). Organic acidic extracts were combined, washed with brine, dried over anhydrous $MgSO_4$ and concentrated. Crude 3-mesityl-3-oxopropanal was obtained as a yellowish oil in 72 % yield (5.2 g, 27.36 mmol). Crude dicarbonyl compound was directly solved in 100 mL of ethanol and hydrazine monohydrate was added (1.3 mL, 27.4 mmol, 1 eq.). The yellow solution was refluxed for 15 h. Once at room temperature the solvent was evaporated. Crude **1e** was purified by recrystallization in hot toluene. After recovering two crystallization crops, 3-mesityl-1-*H*-pyrazole was obtained as white crystalline needles in 85 % yield (4.4 g, 23.6 mmol). mp:188-191 °C (described mp:184-186 °C). NMR data were consistent with literature.[S7]
^{1}H NMR ($CDCl_3$, 400 MHz, 25 °C): δ 7.51 (d, 1H, J = 2.0 Hz), 6.90 (s, 2H), 6.17 (d, 1H, J = 2.0 Hz), 2.33 (s, 3H), 2.05 (s, 6H); ^{13}C{^{1}H} NMR ($CDCl_3$, 100 MHz, 25 °C): δ 144.0, 138.2, 137.9, 136.1, 128.3, 128.2, 105.7, 21.2, 20.4.

4-bromo-3-mesityl-1-*H*-pyrazole (1f).

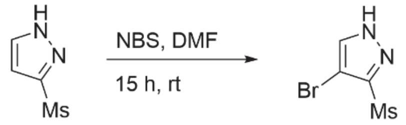

1e **1f**

4-bromo-3-mesityl-1-H-pyrazole (**1f**) was prepared through a modified procedure of a described method.[S4] To a solution of **1e** (3 g, 16.1 mmol, 1 eq.) in 16 mL of *N,N*-dimethylformamide was added *N*-bromosuccinimide at once (2.9 g, 16.4 mmol, 1.02 eq.). The solution was stirred for 15 h at room temperature and diluted with 50 mL of water. The product was extracted with ethyl acetate (4 x 20 mL). The organic extracts were combined, washed with water (3x 15 mL), and brine, dried over anhydrous $MgSO_4$, and concentrated. Crude **1f** was purified through a short column chromatography using *n*-hexane:AcOEt 5:1 as eluent. Pure 4-bromo-3-mesityl-1-*H*-pyrazole (**1f**) was obtained as a white solid in 75 % yield (3.2 g, 12.1 mmol). mp:203-206 ^{o}C.

^{1}H NMR ($CDCl_3$, 300 MHz, 25 ^{o}C): δ 9.53 (s, 1H), 7.56 (s, 1H), 6.93 (s, 2H), 2.32 (s, 3H), 2.03 (s, 6H); ^{13}C{^{1}H} NMR ($CDCl_3$, 75 MHz, 25 ^{o}C): δ 142.8, 139.5, 138.4, 138.3, 128.4, 125.5, 95.0, 21.4, 20.4. HRMS (+ESI) for $(M+H)^+$ $C_{12}H_{14}BrN_2^+$ (*m/z*) calcd 265.0340, found 265.0333.

4-carboxaldehyde-3-phenyl-1-*H*-pyrazole (1g).

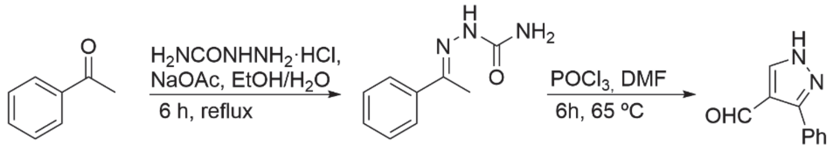

1g

4-carboxaldehyde-3-phenyl-1-H-pyrazole (**1g**) was prepared according to a described procedure.[S8] To a solution of sodium acetate (0.53 g, 6.25 mmol, 1.3 eq.) and acetophenone (0.55 mL, 4.8 mmol, 1.eq.) in 90 mL of ethanol is added semicarbazide hydrochloride (0.56 g, 5 mmol, 1.05 eq.) in 90 mL of water. The resulting suspension is warmed to 100 ^{o}C in an oil bath for 6 h and stirred at room temperature for additional 18 h. Solvent is evaporated to yield crude semicarbazone as a white crystalline solid. Crude product is directly solved in 4.5 mL of anhydrous DMF and cooled to 0 ^{o}C. Phosphorous(V) oxychloride (1 mL, 10.7 mmol, 2.2 eq.) is added dropwise. After 30 min stirring at 0 ^{o}C the solution is warmed to 65 ^{o}C in an oil bath for 6 h. Once at room temperature, the mixture Is poured onto 30 mL of iced water and neutralized with aqueous 2M NaOH solution. The aqueous solution is extracted with AcOEt (3 x 20 mL). Organic phases were combined, washed with brine, dried over anhydrous $MgSO_4$, and concentrated. Crude **1g** is purified through crystallization from chloroform. Pure 4-carboxaldehyde-3-phenyl-1-*H*-pyrazole (**1g**) is obtained as a colorless crystalline solid (0.51 g, 2.97 mol, 51 % yield). mp:139-141 ^{o}C (described mp:145 ^{o}C) Its NMR data were consistent with literature values.[S9]

^{1}H NMR (DMSO-d_6, 500 MHz, 25 ^{o}C): δ 13.76 (bs, 1H), 9.90 (s, 1H), 8.36 (bs, 1H), 7.81 (d, 2H, *J* = 7.6 Hz), 7.52-7.47 (m, 3H). IR (cm^{-1}) 1630 (C=O) 1481, 1305, 1319, 771.

3,5-dimethyl-4-nitro-1-*H*-pyrazole (1h).

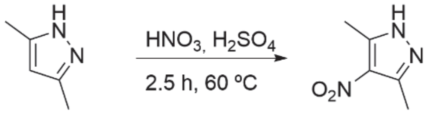

1h

3,5-dimethyl-4-nitro-1-H-pyrazole (**1h**) was prepared through a described method.[S6] Commercial 3,5-dimethylpyrazole (4.8 g, 50 mmol, 1 eq.) was solved in 30 mL of cold concentrated sulfuric acid. The solution was warmed to 60 ºC in an oil bath and 65 % aqueous nitric acid (6.5 mL, 55 mmol, 1.1 eq.) was added. The mixture was maintained at that temperature for 2.5 hours. After cooling to room temperature, the mixture was poured onto 60 g of iced water. The aqueous phase was thoroughly extracted with ethyl acetate (5 x 40 mL). Organic phases were combined, washed with brine, dried over anhydrous $MgSO_4$, and concentrated. Crude **1h** was purified through short column chromatography using *n*-hexane:AcOEt 2:1 as eluent. 3,5-dimethyl-4-nitro-1-H-pyrazole (**1h**) was obtained in 93 % yield as a pale-yellow solid (6.6 g, 46.8 mmol). mp: 121-122 ºC (described mp:123-124 ºC) Its NMR data were consistent with literature values.[S10]

^1H NMR ($CDCl_3$, 500 MHz, 25 ºC): δ 10.48 (s, 1H), 2.62 (s, 6H); $^{13}C\{^1H\}$ NMR ($CDCl_3$, 125 MHz, 25 ºC): δ 144.6, 131.2, 13.1.

3,5-diisopropyl-4-nitro-1-*H*-pyrazole (1i).

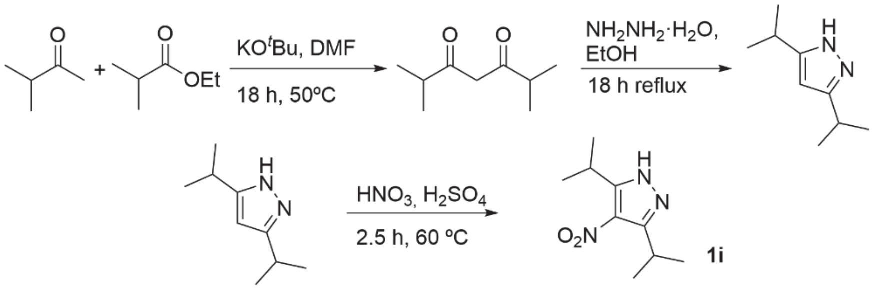

1i

3,5-diisopropyl-4-nitro-1-H-pyrazole (**1i**). 2,6-dimethyl-3,5-heptadione was prepared according to a described procedure.[11] Potassium *tert*-butoxide (1.7g, 15 mmol, 1.5 eq.) was suspended in 1.5 mL of anhydrous DMF and warmed to 50 ºC in an oil bath. The mixture of 3-methyl-2-butanone (1.1 mL, 10 mmol, 1 eq.) and ethyl 2-methylpropanoate (4.03 mL, 30 mmol, 3 eq.) was added dropwise during 30 min. The mixture was stirred 8 hours at that temperature. Once at room temperature, the solution was diluted with 30 mL of water and acidified with 50% aqueous sulfuric acid until pH 1-2. The aqueous phase was extracted with diethyl ether (3 x 20 mL). Organic phases were combined, washed with brine, dried over anhydrous $MgSO_4$, and concentrated. Crude 2,6-dimethyl-3,5-heptadione was obtained as a yellowish oil in 90 % yield (1.44 g, 9 mmol). Dicarbonyl compound was directly solved in 50 mL ethanol and hydrazine monohydrate was added (0.44 mL, 9 mmol, 1 eq.). The yellow solution was heated to reflux 15 h in an oil bath. Once cold, solvent was evaporated to yield 3,5-diisopropyl-1-*H*-pyrazole as a yellowish solid (1.0 g, 6.6 mmol, 73 % yield).

Crude 3,5-diisopropyl-1-*H*-pyrazole (1.0 g, 6.6 mmol, 1 eq.) was directly solved in 4 mL cold concentrated sulfuric acid and the solution warmed to 60 ºC. Aqueous 65 % nitric acid (0.76 mL, 7.26 mmol, 1.1 eq.) was added and the mixture stirred at that temperature for 4.5 h. Once at room temperature the reaction mixture was poured onto

40 mL of iced water. Aqueous phase was extracted with AcOEt (3 x 20 mL). Organic phases were combined, washed with brine, dried over anhydrous $MgSO_4$, and concentrated. Crude **1i** was purified through column chromatography using *n*-hexane:AcOEt 2:1 as eluent. Pure 3,5-diisopropyl-4-nitro-1*H*-pyrazole was obtained as a crystalline pale-yellow solid in 77 % yield (1.0 g, 5.1 mmol). mp:118-122 °C (described mp:112 °C) NMR data were consistent with literature values.[S12]

^{1}H NMR ($CDCl_3$, 300 MHz, 25 °C): δ 3.70 (hept, 1H, J = 6.9Hz), 1.35 (d, 6H, J = 6.9Hz); ^{13}C NMR ($CDCl_3$, 75 MHz, 25 °C): δ 153.6, 129.0, 26.6, 21.2.

3. Optimization of $3a_{Na}$ formation.

Table S1 summarizes the conditions assayed during the optimization of **$3a_{Na}$** synthesis. Dichloroborane was preferred over dibromoborane to facilitate the resulting salt removal through filtration due to the lower solubility of chloride salts in organic solvents compared to their bromide counterparts. Among commercially available dichloroboranes, the dimethyl sulfide complex was chosen as starting boron source due to its higher stability compared to the tetrahydrofuran complex. We selected 3-*tert*-butylpyrazole as standard heterocycle for reaction optimization.

Preparation of **$3a_{Na}$** was firstly attempted in dichloromethane. In the absence of a base (entry 1) the reaction did not complete due to starting pyrazole protonation with the HCl evolved from the reaction between **1a** and dichloroborane. Addition of triethylamine or CsOAc (entries 2 and 6) increased the reaction performance, but yield was still low. Furthermore, in the first case ammonium salt formed was difficult to remove from the reaction mixture. Use of DBU, DMAP or K_2CO_3 (entries 3, 4, and 5) as base yielded complex mixtures due to the reaction between the boron source and the base. We explored then the formation *in situ* of sodium pyrazolide with NaH and its direct reaction with the haloborane. This alternative presented as an advantage the formation of NaCl as concomitant product, easy to filter off from the reaction media. Use of THF resulted in the reaction of the solvent with the haloborane under the reaction conditions providing an unidentified mixture of products (entries 7 and 12). Less coordinating toluene gave better results than donating diethyl ether able to form complexes with the starting borane reducing its reactivity (entries 10 and 11 *vs* entries 9). Extension of the reaction time from 2 hours to 24 hours gave the best result (entry 11). Equivalents of pyrazole and base were not further explored due to the good results obtained with the ideal ca. stoichiometric amounts. Conditions of entry 11 were chosen as standard conditions for all poly(pyrazolyl)borates prepared. These optimized conditions were used with small adaptations for the synthesis of bis- and tetrakis(pyrazolyl)borates. For Bp^x (**2**) ligands pyrazole and base equivalents were reduced to 2.05 equivalents and $BH_2Cl{\cdot}SMe_2$ used as boron source. Preparation of Tkp^x (**4**) ligands was performed using 4.1 equivalents of pyrazole and base and $BCl_3{\cdot}SMe_2$.Satisfactory results were obtained in both cases.

Table S1. Reaction optimization conditions for $\mathbf{3a_{Na}}$.[a]

Entry	Base	Solvent	Temperature (°C)	Time (h)	Conversion into 3_{Na} (%)[b]
**1**	none	DCM	rt	2	15
**2**	Et_3N	DCM	rt	2	25
**3**	DBU	DCM	rt	2	complex mixture
**4**	DMAP	DCM	rt	2	complex mixture
**5**	K_2CO_3	DCM	rt	2	complex mixture
**6**	CsOAc	DCM	rt	2	24
**7**	Et_3N	THF	rt	2	complex mixture
**8**	Et_3N	toluene	rt	2	50
**9**	NaH	Et_2O	rt	24	60
**10**	NaH	toluene	rt	2	65
**11**	**NaH**	**toluene**	**rt**	**24**	**80**
**12**	NaH	THF	rt	2	complex mixture

[a] Reaction conditions: **1a** (3.1 mmol), base (3.1 mmol), solvent (10 mL) 10 min at 0 °C, $BHCl_2{\cdot}SMe_2$ (1 mmol) 24 h at rt. Evaporation and solution in $CDCl_3$. [b] NMR conversion into $\mathbf{3a_{Na}}$.

4. General synthesis of thallium(I) hydrotris(pyrazolyl)borates (3_{Tl})

Pyrazole **1a-j** (12.1 mmol, 3.02 eq.) was solved in 40 mL of anhydrous toluene and cooled to 0 °C on an ice bath. Sodium hydride (60% dispersion in mineral oil, 484 mg, 12.1 mmol, 3.02 eq.) was added at once. After 10 min stirring at that temperature, dichloroborane dimethyl sulfide complex (0.46 mL, 4 mmol, 1 eq.) was quickly added. The resulting suspension was stirred at room temperature for 24 h. Toluene was evaporated and replaced with 30 mL tetrahydrofuran. Thallium(I) acetate (1.58 g, 6 mmol, 1.5 eq.) was added and the suspension stirred at room temperature for 2.5 h. Precipitate was removed through filtration and washed twice with 10 mL THF. The filtrates were combined and evaporated, and the white solid obtained washed with *n*-hexane (3 x 10 mL), and methanol (3 x 15 mL), and dried under vacuum on a rotatory pump to yield pure thallium(I) hydrotris(pyrazolyl)borate $\mathbf{3a_{Tl}}$, $\mathbf{3b_{Tl}}$, $\mathbf{3c_{Tl}}$, $\mathbf{3d_{Tl}}$, $\mathbf{3e_{Tl}}$, and $\mathbf{3f_{Tl}}$. Sodium complexes were isolated for $\mathbf{3g_{Na}}$ and $\mathbf{3h_{Na}}$.

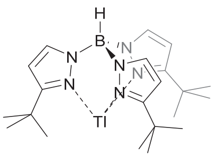

thallium(I) hydrotris(3-*tert*-butylpyrazol-1-yl)borate ($3a_{Tl}$). 1.83 g, 78% yield over 5 mmol scale; 4,6 g, 92 % yield over 8,4 mmol scale; white solid; mp:195 °C.

^{1}H NMR ($CDCl_3$, 500 MHz, 25 °C): δ 7.56 (d, 3H, *J* = 2.2 Hz), 6.05 (d, 3H, *J* = 2.2 Hz), 1.37 (s, 27); ^{11}B NMR ($CDCl_3$, 96 MHz, 25°C): δ -1.24 (d, $J_{B\text{-}H}$ = 106.4 Hz, $\Delta_{1/2}$ = 208 Hz); ^{13}C{^{1}H} NMR ($CDCl_3$, 125 MHz, 25 °C): δ 163.6 (broad signal), 136.4, 101.3, 32.4, 31.9 (d, $J_{C\text{-}Tl}$ = 161 Hz); IR (cm^{-1}) 2953, 2864, 2430 (B-H), 1502, 1355, 1340, 1196, 1048, 733. Anal. Calcd for C21H34BN6Tl ($Tp^{tBu}Tl$): C, 43.06; H, 5.85; N, 14.35. Found: C, 42.90; H, 5.99; N, 14.15.

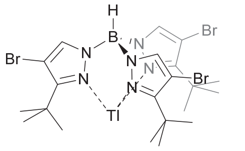

thallium(I) hydrotris(4-bromo-3-*tert*-butylpyrazol-1-yl)borate ($3b_{Tl}$). 3.16 g, 96 %; white solid; mp:280 ^{o}C.

^{1}H NMR ($CDCl_3$, 500 MHz, 25 ^{o}C): δ 7.61 (s, 3H), 1.46 (s, 27); ^{11}B NMR ($CDCl_3$, 160 MHz, 25°C): δ -1.24 (d, J_{B-H} = 108 Hz, $\Delta_{1/2}$ = 204 Hz); ^{13}C{^{1}H} NMR ($CDCl_3$,125 MHz, 25 ^{o}C): δ 158.5 (d, J_{C-Tl} = 38.4 Hz), 139.8, 91.6 (d, J_{C-Tl} = 23.6 Hz), 34.1, 31.4, 29.9, 28.6; IR (cm^{-1}): 2956, 2864, 2428 (B-H), 1505, 1359, 1131, 1027, 737. Anal. Calcd for C21H31BBr3N6Tl ($Tp^{tBu,Br}Tl$): C, 30.67; H, 3.80; N, 10.22. Found: C, 30.76; H, 3.55; N, 10.18.

Single crystals susceptible for its study through X ray diffraction were obtained through slow evaporation of a solution of **$3b_{Na}(OH_2)$** in AcOEt/*n*-hexane.

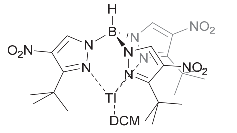

thallium(I) hydrotris(3-*tert*-butyl-4-nitropyrazol-1-yl)borate ($3c_{Tl}$·DCM). 2.68 g, 93 %; tanned solid; mp:230 ^{o}C (decomp.).

^{1}H NMR (acetone-d_6, 500 MHz, 25 ^{o}C): δ 8.44 (s, 3H),1.40 (s, 27 H); ^{11}B NMR (acetone-d_6, 96 MHz, 25°C): δ -1.72 (d, J_{B-H} = 118.5 Hz, $\Delta_{1/2}$ = 212 Hz); ^{13}C{^{1}H} NMR (acetone-d_6, 125 MHz, 25 ^{o}C): δ 155.8, 138.5, 134.1, 34.2, 28.9; IR (cm^{-1}): 3119, 2965, 2397 (B-H), 1735, 1519, 1315, 1127, 815. Anal. Calcd for C22H33BCl2N9O6Tl ($Tp^{tBu,NO2}Tl$·DCM): C, 32.80; H, 4.13; N, 15.65. Found: C, 32.35; H, 3.97; N, 15.82.

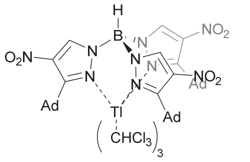

thallium hydrotris(3-adamantyl-4-nitropyrazol-1-yl)borate ($3d_{Tl}$·3CHCl3). 487 mg, 51 %; yellowish solid; mp:190-197 ^{o}C.

^{1}H NMR ($CDCl_3$, 500 MHz, 25 ^{o}C): δ 8.27 (s, 3H), 2.37-2.12 (m, 15H), 1.97-1.80 (m, 30H); ^{11}B NMR (acetone-d_6, 160 MHz, 25°C): δ -7.65 (d, J_{B-H} = 46 Hz, $\Delta_{1/2}$ = 78 Hz); ^{13}C{^{1}H} NMR ($CDCl_3$, 125 MHz, 25 ^{o}C): δ 149.8 (bs), 138.2, 133.7 (bs), 68.9, 46.6, 44.5, 38.7, 37.4, 34.8, 30.4; IR (cm^{-1}): 2986, 2867, 2757, 2653 (B-H), 2608 (B-H), 1598, 1360, 1147, 831. Anal. Calcd for C114H174B2Cl18N18O12Tl2 ($Tp^{Ad,NO2}Tl$·3$CHCl_3$·2,5 Hex): C, 44.79; H, 5.74 N, 8.25. Found: C, 44.69; H, 5.40; N, 8.05.

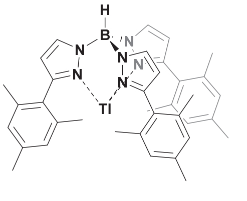

thallium(I) hydrotris(3-mesitylpyrazol-1-yl)borate ($3e_{Tl}$). 2.5 g, 81 %; white solid; mp:292-294 ^{o}C (described mp:305-308 ^{o}C). NMR data were consisted with literature.[S7]

^{1}H NMR (CD_2Cl_2, 500 MHz, 25 ^{o}C): δ 7.86 (d, 3H, J = 2.1 Hz), 6.83 (s, 6H), 6.08 (d, 3H, J = 2.1 Hz), 2.25 (s, 9H), 1.89 (s, 18H); ^{11}B NMR (CD_2Cl_2, 160 MHz, 25°C): δ -1.65 (d, J_{B-H} = 83 Hz, $\Delta_{1/2}$ = 210 Hz); ^{13}C{^{1}H} NMR (CD_2Cl_2, 125 MHz, 25 ^{o}C): δ 150.9 (bs), 137.9, 137.7, 136.2, 131.3, 128.2, 105.3 (bs), 21.2, 20.5 (d, J_{C-Tl}=38.0 Hz); IR (cm^{-1}): 2919, 2438 (B-H), 1478, 1183, 1042, 774.

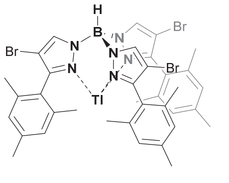

thallium(I) hydrotris(4-bromo-3-mesitylpyrazol-1-yl)borate ($3f_{Tl}$). 3.14 g, 78%; white solid; mp: 315-317 ^{o}C (dec.).

^{1}H NMR (CD_2Cl_2, 500 MHz, 25 ^{o}C): δ 7.89 (s, 3H), 6.87 (s, 6H), 2.27 (s, 9H), 1.87 (s, 18H); ^{11}B NMR (CD_2Cl_2, 160 MHz, 25°C); δ -1.90 (d, J_{B-H} = 106 Hz, $\Delta_{1/2}$ = 230 Hz); ^{13}C{^{1}H} NMR (CD_2Cl_2, 125 MHz, 25 ^{o}C): δ 150.7 (bs), 139.0, 138.9, 138.1, 136.9, 128.4, 94.2 (bs), 21.3, 20.1 (d, J_{C-Tl} = 34.0 Hz); IR (cm^{-1}): 2969, 2921, 2853, 2501 (B-H), 2361 (B-H), 1738, 1355, 1205, 1099.

Anal. Calcd for C184H193B5Br15N30OTl5 ($Tp^{Ms,Br}Tl\cdot 1/5THF$): C, 43.20; H, 3.80; N, 8.21. Found: C, 43.44; H, 3.68; N, 8.24.

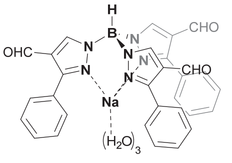

sodium hydrotris(4-carboxaldehyde-3-phenylpyrazol-1-yl)borate ($3g_{Na}$). 1.90 g, 79 %; white solid; mp:240-241 °C.
^1H NMR ($CDCl_3$, 300 MHz, 25 °C): δ 9.95 (s, 3H), 8.09 (s, 3H), 7.65-7.64 (m, 6H), 7.50-7.48 (m, 9H); ^{11}B NMR ($CDCl_3$, 96 MHz, 25°C): δ -2.90 (d, J_{B-H} = 180 Hz); IR (cm^{-1}): 3140, 2951, 2920, 2867, 2323 (B-H), 1638, 1480, 1324, 1076. Anal. Calcd for C30H28BN6NaO6 (TpPh,CHONa·3H2O): C, 59.82; H, 4.68; N, 13.95. Found: C, 60.29; H, 4.92; N, 13.88.

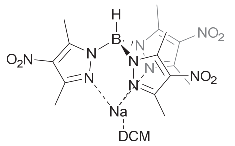

sodium hydrotris(3,5-dimethyl-4-nitropyrazol-1-yl)borate ($3h_{Na}{}^{\cdot DCM}$). 1.86 g, 86 %; white solid; mp: 308-310 °C (decomp.).
^1H NMR ($CDCl_3$, 500 MHz, 25 °C): δ 2.78 (s, 9 H), 2.63 (s, 9 H); ^{11}B NMR ($CDCl_3$, 96 MHz, 25°C): δ -8.06 (d, J_{B-H} = 111 Hz, $\Delta_{1/2}$ = 214 Hz); $^{13}C\{^1H\}$ NMR ($CDCl_3$, 125 MHz, 25 °C): δ 147.7, 146.33, 132.4, 15.0, 14.1, 12.9; IR (cm^{-1}): 2967, 2923, 2505 (B-H), 1551, 1347, 1200, 992. Anal. Calcd for C16H23BCl2N9NaO7 ($Tp^{Me2,NO2}Na\cdot DCM\cdot H_2O$): C, 34.43; H, 4.16; N, 22.59. Found: C, 34.77; H, 3.81; N, 22.90. HRMS (+ESI) for $(Tp^{Me2,NO2}H+H)^+$ $C_{15}H_{20}BN_9O_6^+$ (*m/z*) calcd: 434.1702, found: 434.1710; $(Tp^{Me2,NO2}Na+H)^+$ $C_{15}H_{29}BN_9NaO_6^+$ (*m/z*) calcd: 456.1527, found: 456.1525.

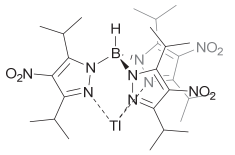

thallium(I) hydrotris(3,5-diisopropyl-4-nitropyrazol-1-yl)borate ($3i_{Tl}$). Observed in the ^1H NMR of the reaction crude. The visible signals are listed below.
^1H NMR (DMSO-d_6, 300 MHz, 25 °C): δ 2.21 (d, 18H, J = 6.4 Hz, 6CH_3), 2.21 (d, 18H, J = 6.4 Hz, 6CH_3); $^{11}B\{^1H\}$ NMR (DMSO-d_6, 96 MHz, 25°C): δ -6.3.

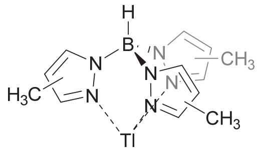

thallium(I) hydrotris[3(5)-methylpyrazol-1-yl)borate ($3j_{Tl}$). 1.56 g, 85 %; white solid.
The four possible regioisomers were formed and could not be separated. The signals for ^{11}B NMR spectrum are listed.
$^{11}B\{^1H\}$ NMR ($CDCl_3$, 160 MHz, 25°C): δ -2.0, -3.8, -5.7, -7.7.

5. General synthesis of thallium(I) dihydrobis(pyrazolyl)borates (2_{Tl})

Pyrazole **1a-f** (10.1 mmol, 2.02 eq. for **1a-c** or 20.1 mmol, 4.02 eq. for **1f**) was solved in 50 mL of anhydrous toluene and cooled to 0 °C on an ice bath. Sodium hydride (60% dispersion in mineral oil, 410 mg, 10.1 mmol, 2.02 eq. for **1a-c** or 840mg, 20.1 mmol, 4.02 eq. for **1f**) was added at once. After 10 min stirring at that temperature, chloroborane dimethyl sulfide complex (1.04 mL, 5 mmol, 1 eq.) was quickly added. The resulting suspension was stirred at room temperature for 24 h. Toluene was evaporated and replaced with 24 mL of tetrahydrofuran. Thallium(I) acetate (1.8 g, 7.5 mmol, 1.5 eq.) was added and the suspension stirred for 2.5 h at room temperature. Precipitate was removed through filtration and washed twice with 10 mL THF. The filtrates were combined and evaporated, and the white solid obtained washed with

n-hexane (3 x 10 mL) and EtOH (3 x 10 mL) and dried on a rotatory pump. Pure thallium(I) dihydrobis(pyrazolyl)borates **2a-f$_{Tl}$** were obtained as white solids.

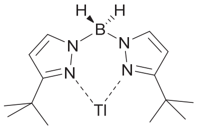

thallium(I) dihydrobis(3-*tert*-butylpyrazol-1-yl)borate (2a$_{Tl}$). 2.63 g, 90 %; white solid; mp:200-201 °C.
^{1}H NMR (CDCl$_3$, 500 MHz, 25 °C): δ 7.35 (d, 2H, *J* = 2.1 Hz), 5.98 (d, 2H, *J* = 2.1 Hz), 1.34 (s, 18 H); ^{11}B NMR (CDCl$_3$, 160 MHz, 25°C): δ -8.77 (t, J_{B-H} = 100 Hz, $\Delta_{1/2}$ = 250 Hz); ^{13}C{^{1}H} NMR (CDCl$_3$, 125 MHz, 25 °C): δ 163.4, 136.7, 100.8, 32.8, 31.9; IR (cm^{-1}): 2962, 2930, 2870, 2486 (B-H), 2394 (B-H), 2361 (B-H), 1498, 1360, 1139, 781. Anal. Calcd for C146H254B10N40Tl10 (p^{tBu}Tl·0.1*n*-Hex): C, 37.14; H, 5.42; N, 11.87. Found: C, 37.23; H, 5.35; N, 11.85.

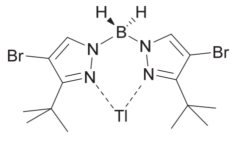

thallium(I) dihydrobis(4-bromo-3-*tert*-butylpyrazol-1-yl)borate (2b$_{Tl}$). 2.7 g, 87 %; white solid; mp:259 °C.
^{1}H NMR (CDCl$_3$, 500 MHz, 25 °C): δ 7.53 (s, 2H), 1.60 (s, 18 H); ^{11}B NMR (CDCl$_3$, 160 MHz, 25°C): δ -9.1 (t, J_{B-H} = 110 Hz; $\Delta_{1/2}$ = 375 Hz); ^{13}C{^{1}H} NMR (CDCl$_3$, 125 MHz, 25 °C): δ 152.9, 137,3, 91.3, 34.5, 31.0; IR (cm^{-1}): 2962, 2930, 2870, 2487 (B-H), 2394 (B-H), 2361 (B-H), 1498, 1139, 781. Anal. Calcd for C18H30BBr2N4OTl (p^{tBu,Br}Tl·THF): C, 31.38; H, 4.36; N, 8.08. Found: C, 30.96; H, 4.58; N, 7.86. HRMS (+ESI) for (Bp^{tBu,Br}H+H)$^{+}$ $C_{14}H_{23}BBr_2N_4^{+}$ (*m/z*) calcd 419.0438, found 419.0442.

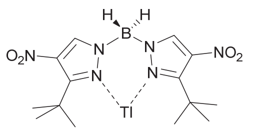

thallium(I) dihydrobis(3-*tert*-butyl-4-nitropyrazol-1-yl)borate (2c$_{Tl}$). 2.55 g, 92%; white solid; mp:315 °C decomposes.
^{1}H NMR (CD$_2$Cl$_2$, 500 MHz, 25 °C): δ 8.22 (s, 2H), 1.50 (s, 18 H); ^{11}B NMR (CDCl$_3$, 160 MHz, 25°C): δ -8.1 (t, J_{B-H} = 97 Hz, $\Delta_{1/2}$ = 270 Hz); ^{13}C{^{1}H} NMR (CDCl$_3$, 125 MHz, 25 °C): δ 151.1, 138.9, 133.2, 33.2, 27.8; IR (cm^{-1}): 2962, 2932, 2411 (B-H) 2324 (B-H), 2255 (B-H), 1510, 1311, 766. cm^{-1}): 2962, 2930, 2870, 2486 (B-H), 2394 (B-H), 2361 (B-H), 1498, 1360, 1139, 781. Anal. Calcd for C357H564B25Cl14N150O100Tl25 (Bp^{tBu,NO2}Tl·0.28DCM): C, 29.71; H, 3.94; N, 14.56. Found: C, 30.10; H, 3.96; N, 14.17. HRMS (+ESI) for (*Bp^{tBu,NO2}H*+H)$^{+}$ $C_{14}H_{23}BN_6O_4^{+}$ *m/z* calcd 351.1947, found: 351.1954.
Single crystals susceptible for its study through X ray diffraction were obtained through slow evaporation of a solution of **2c$_{Tl}$** in AcOEt/*n*-hexane.

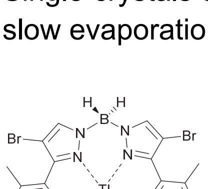

thallium(I) dihydrobis(4-bromo3-mesitylpyrazol-1-yl)borate (2f$_{Tl}$). 2.83 g, 76 %; white solid; mp:264 °C (decomp.).
^{1}H NMR (CDCl$_{30}$, 500 MHz, 25 °C): δ 7.59 (s, 2H), 6.94 (s, 4H), 2.32 (s, 6H), 2.03 (s, 12H); ^{11}B NMR (CDCl$_3$, 160 MHz, 25°C): δ= 8.3 (broad signal; $\Delta_{1/2}$ = 480 Hz); ^{13}C{^{1}H} NMR (CDCl$_3$, 125 MHz, 25 °C): δ 139.8, 138.7, 130.0, 128.5, 128.4, 125.6, 95.0, 21.3, 19.9; IR (cm^{-1}): 2948, 2911, 2848, 2490 (B-H), 2418 (B-H), 1612, 1430, 1283. Anal. Calcd for C99H107B4Br8Cl9N16Tl4 (Bp^{Ms,Br}Tl·3/4CHCl$_3$): C, 35.42; H, 3.22; N, 6.71. Found: C, 35.27; H, 3.18; N, 6.63.

6. Caracterization of pyrazabole derivatives from 1e-f

For pyrazoles **1e-f** the preparation of the corresponding **2e-f**$_{\mathbf{Tl}}$ complexes under standard conditions provided the expected complexes in low yields mixed with the corresponding pyrazabole compounds already described by S. Trofimenko with other substituents.[13] Enriched samples of pyrazaboles susceptible for NMR identification were obtained through column chromatography using *n*-hexane:DCM 10:1 to 1:1 as eluent.

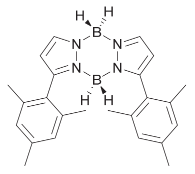

1,7-bis(mesityl)pyrazabole. 83 mg, 8 %; white solid.
^{1}H NMR (CDCl$_3$, 500 MHz, 25 °C): δ 7.26 (d, 2H, *J* = 2.2 Hz), 6.38 (s, 4H), 5.71 (d, 2H, *J* = 2.2 Hz), 1.82 (s, 6H), 1.48 (s, 12H); ^{11}B NMR (CDCl$_3$, 160 MHz, 25°C): δ -8.3 (broad signal), -11.8 (broad signal).

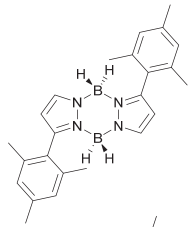

1,5-bis(mesityl)pyrazabole. 70 mg, 6 %; white solid.
^{1}H NMR (CDCl$_3$, 500 MHz, 25 °C): δ 7.60 (d, 2H, *J* = 2.3 Hz), 6.99 (s, 4H), 6.19 (d, 2H, *J* = 2.3 Hz), 2.38 (s, 6H), 2.02 (s, 12H); ^{11}B NMR (CDCl$_3$, 160 MHz, 25°C): δ -10.16 (broad signal).

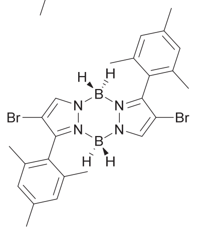

2,6-dibromo-1,5-bis(mesityl)pyrazabole. 207 mg, 15 %; white solid.
^{1}H NMR (CDCl$_3$, 500 MHz, 25 °C): δ 7.64 (s, 2H), 6.98 (s, 4H), 2.37 (s, 6H), 1.98 (s, 12H); ^{11}B NMR (CDCl$_3$, 160 MHz, 25°C): δ -9.8 (broad signal; $\Delta_{1/2}$ = 492 Hz); ^{13}C{^{1}H} NMR (CDCl$_3$, 125 MHz, 25 °C): δ 146.9, 139.8, 138.0, 135.6, 128.4, 124.9, 95.3, 21.4, 20.2; IR (cm^{-1}): 2918 2479 (B-H), 2415 (B-H), 2348 (B-H), 1642, 1158, 851.

7. General synthesis of sodium tetrakis(pyrazolyl)borates (4$_{\mathbf{Na}}$)

Pyrazole **1a-f** (4.1 mmol, 4.1 eq.) was solved in 15 mL of anhydrous toluene and cooled to 0 °C on an ice bath. Sodium hydride (60% dispersion in mineral oil, 164 mg, 4.1 mmol, 4.1 eq.) was added at once. After 10 min stirring at that temperature, trichloroborane dimethyl sulfide complex (180 mg, 1 mmol, 1 eq.) was added at once. The resulting suspension was stirred at room temperature for 24 h. Toluene was evaporated. Crude **4**$_{\mathbf{Na}}$ was washed with *n*-hexane (3 x 10 mL). Non reacted pyrazole was removed by heating at 100 °C under vacuum. Pure sodium tetrakis(pyrazolyl)borates **4a-f**$_{\mathbf{Na}}$ were obtained as white solids. NMR analysis shows the inequivalence of the four pyrazole rings. Elemental analysis indicates that these anions often act as doubly bidentated ligands.

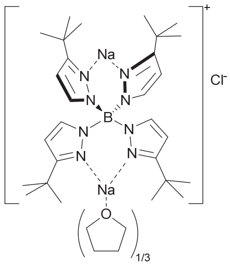

4a$_{Na}$

disodium tetrakis(3-*tert*-butylpyrazol-1-yl)borate chloride (4a$_{\mathbf{Na}}$). 427 mg, 73 %; white solid; mp>340 °C.
^{1}H NMR (DMSO-*d$_6$*, 500 MHz, 25 °C): δ 7.02 (d, 4H, *J* = 2.2 Hz), 5.84 (d, 4H, *J* = 2.2Hz), 1.19 (s, 36 H); ^{11}B NMR (DMSO-*d$_6$*, 96 MHz, 25°C): δ 0.4 (s, $\Delta_{1/2}$ = 3.5 Hz); ^{13}C{^{1}H} NMR (DMSO-*d$_6$*, 125 MHz, 25 °C): δ 159.9, 159.9, 134.1, 134.1, 98.6, 98.6, 31.6, 31.0; IR (cm^{-1}): 3164, 3076, 2963, 2851, 1470, 991, 931, 773. Anal. Calcd for C88H140B3Cl3N24Na6O3 (Tkp^{tBu}Na·1/3THF): C, 57.85 H, 7.72; N, 18.40. Found: C, 57.80; H, 8.00; N, 18.70.

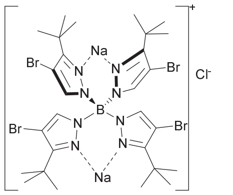

disodium tetrakis(4-bromo-3-*tert*-butylpyrazol-1-yl)borate chloride ($4b_{Na}$). 576 mg, 64 %; white solid; mp>340 ^oC.
^1H NMR (DMSO-*d6*, 500 MHz, 25 ^oC): δ 7.18 (s, 4H), 1.28 (s, 36 H); ^{11}B NMR (DMSO-d_6, 160 MHz, 25^oC): δ -0.1 (s, $\Delta_{1/2}$ = 3.4 Hz); $^{13}C\{^1H\}$ NMR (DMSO-d_6, 125 MHz, 25 ^oC): δ 154.8, 154.8, 136.1, 136.1, 87.9, 87.9, 32.6, 29.2; IR (cm^{-1}): 2962, 1456, 1362, 1126, 810. HRMS (MALDI-QTOF) for $(Tkp^{tBu,Br}Na+Na)^+$ $C_{28}H_{40}BBr_4N_8Na_2^+$ (*m/z)* calcd 864.9962, found 864.9951. Anal. Calcd for C90H142B3Br12Cl3N24Na6O4 ($Tkp^{tBU,Br}Na_2Cl\cdot 1/3Hex\cdot 4H_2O$: C, 37.80; H, 5.01; N, 11.75. Found: C, 37.74; H, 4.68; N, 11.35.

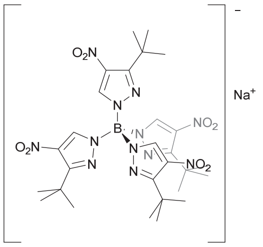

sodium tetrakis(3-*tert*-butyl-4-nitropyrazol-1-yl)borate ($4c_{Na}$). 643 mg, 91 %; white solid; mp:280-283 ^oC.
^1H NMR (C_6D_6, 500 MHz, 25 ^oC); δ 7.80 (s, 4H), 1.19 (s, 36 H); ^{11}B NMR (DMSO-d_6, 160 MHz, 25^oC): δ 0.0 (s, $\Delta_{1/2}$ = 3.8 Hz); $^{13}C\{^1H\}$ NMR (C_6D_6, 125 MHz, 25 ^oC): δ 151.4, 137.2, 133.7, 32.9, 27.6; IR (cm^{-1}): 3236, 2965, 1508, 1388, 1207, 933, 766. Anal. Calcd for C88H130B3N36Na3O26 ($Tkp^{tBu,NO2}Na\cdot 1/3THF\cdot 1/3H_2O$: C, 47.83; H, 5.93; N, 22.82. Found: C, 47.59; H, 6.35; N, 23.19.

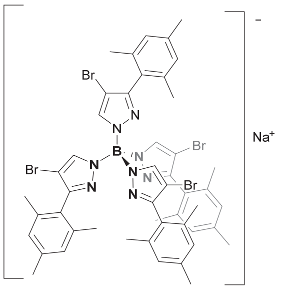

sodium tetrakis(4-bromo-3-mesitylpyrazol-1-yl)borate ($4f_{Na}$). 1.0 g, 93 %; white solid; mp:311-314 ^oC (decomp.).
^1H NMR ($CDCl_3$, 300 MHz, 25 ^oC): δ 7.51 (d, 4H, *J*=2.2 Hz), 6.93 (s, 8H), 2.32 (s, 12H), 2.01 (s, 24H); ^{11}B NMR ($CDCl_3$, 160 MHz, 25^oC): δ 0.6 (s, $\Delta_{1/2}$ = 6.1 Hz); $^{13}C\{^1H\}$ NMR ($CDCl_3$, 75 MHz, 25 ^oC): δ 142.8, 139.5, 138.4, 128.0, 138, 125.0, 94.9 (broad signal), 21.4, 19.9. Anal. Calcd for C114H125B3Br9N18Na3 ($Tkp^{Ms,Br}Na\cdot 1/3Hex$: C, 53.32; H, 4.91; N, 9.82. Found: C, 53.09; H, 4.98; N 9.94. HRMS (+ESI) for $(Tkp^{Ms,Br}H+H)^+$ $C_{48}H_{49}BBr_4N_8^+$ (*m/z)* calcd 1069.0954, found 1069.0961.

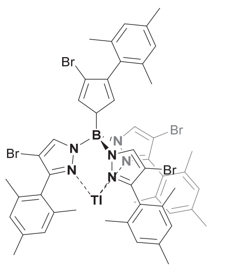

thallium tetrakis(4-bromo-3-mesitylpyrazol-1-yl)borate ($4f_{Tl}$). Metal exchange for the preparation of **$4f_{Tl}$** was performed using 6 mL of THF and 395 mg of TlOAC (1.5 mmol, 1.5 eq). Precipitated was filtered off and THF evaporated. Remaining solid was washed with ethanol. 1.08 g, 85 %; white solid; mp: 270-273 ^oC (decomp.).
^1H NMR (CD_2Cl_2, 500 MHz, 25 ^oC): δ 7.60 (s), 6.95 (s, 8H), 2.32 (s, 12H), 2.02 (s, 24H); ^{11}B NMR (CD_2Cl_2, 160 MHz, 25^oC): δ 0.84 (s, $\Delta_{1/2}$ = 5.6 Hz); $^{13}C\{^1H\}$ NMR (CD_2Cl_2, 125 MHz, 25 ^oC): δ 143.3 (broad signal), 139.7, 138.7, 138.1, 128.5, 125.6, 95.5 (broad signal), 21.6, 19.9.
Single crystals susceptible for its study through X ray diffraction were obtained through slow evaporation of a solution of **$4f_{Tl}$** in AcOEt/*n*-hexane.

8. NMR, IR and HRMS spectra

^{1}H ($CDCl_3$, 300 MHz) and ^{13}C{^{1}H} ($CDCl_3$, 75 MHz) NMR of **1a**

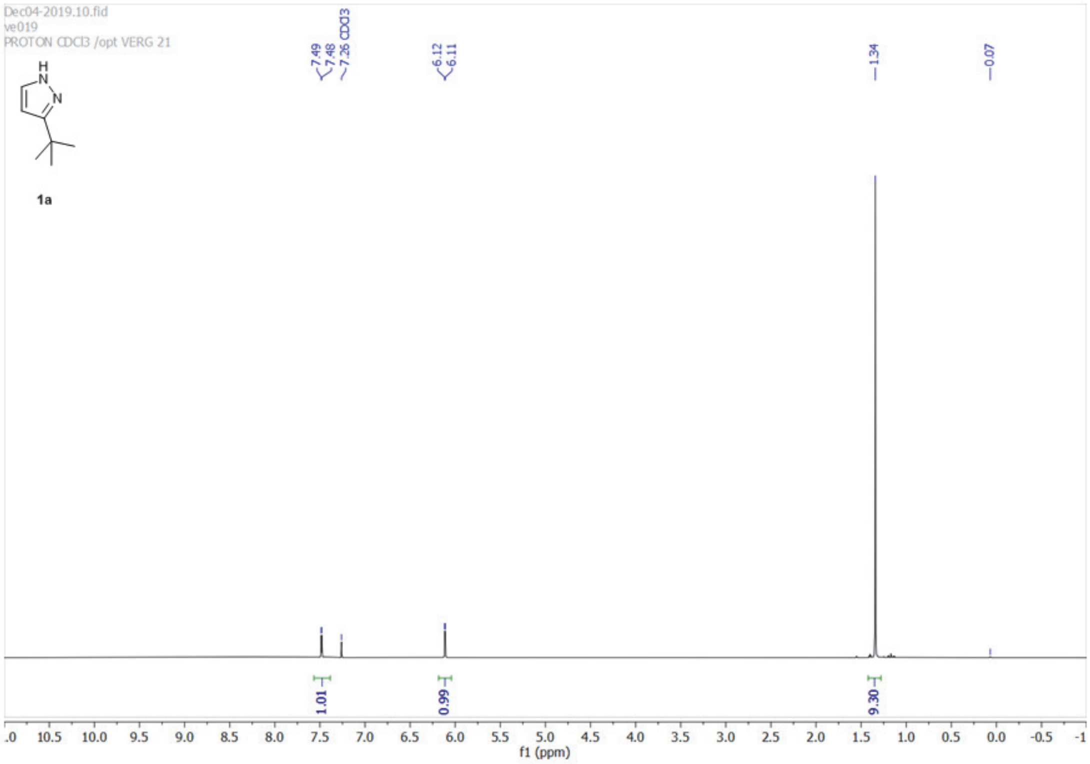

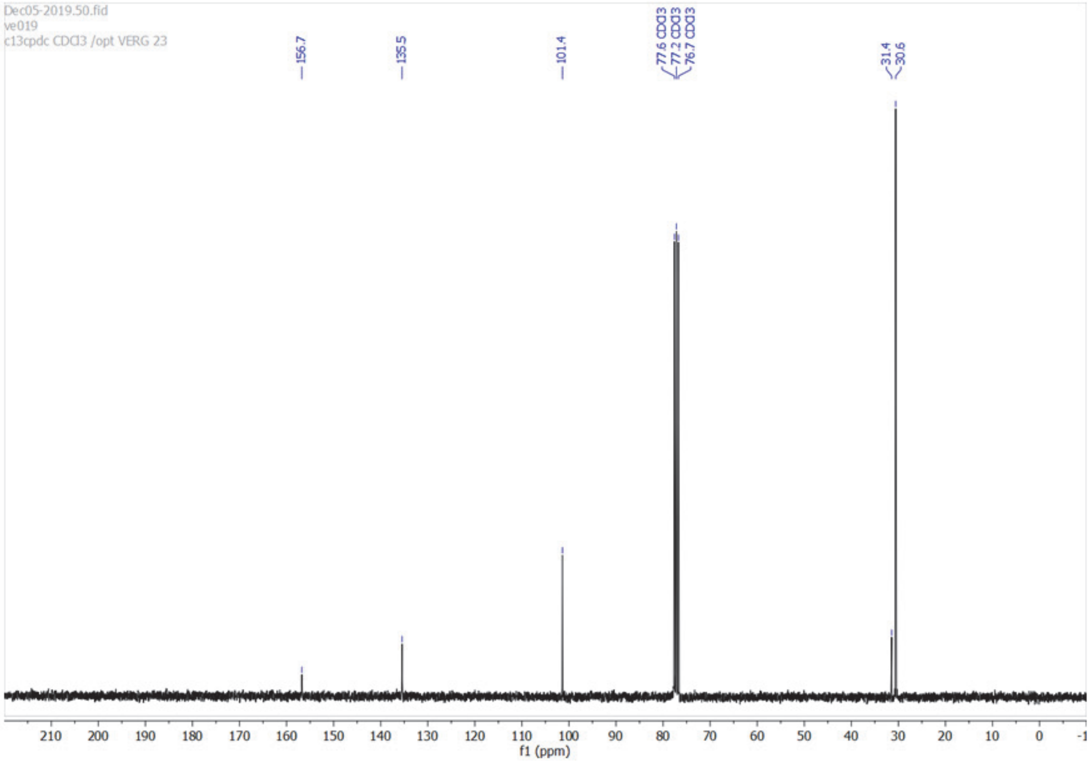

^{1}H ($CDCl_3$, 500 MHz) and $^{13}C\{^{1}H\}$ ($CDCl_3$, 125 MHz) NMR of **1b**

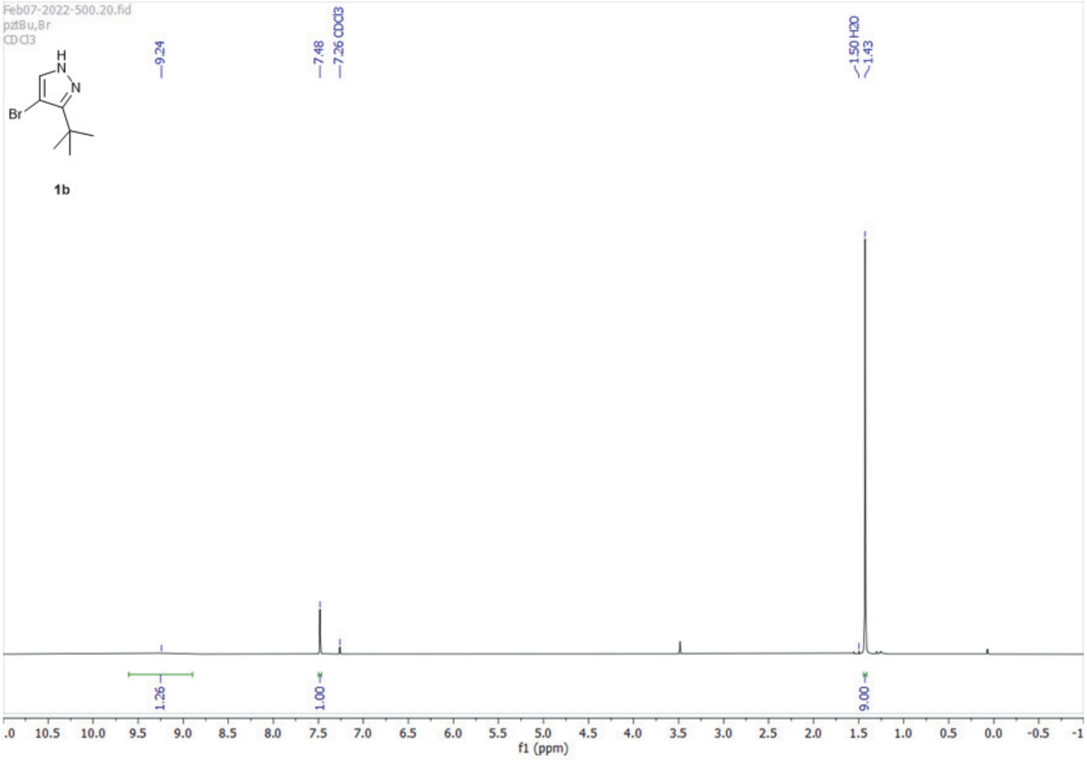

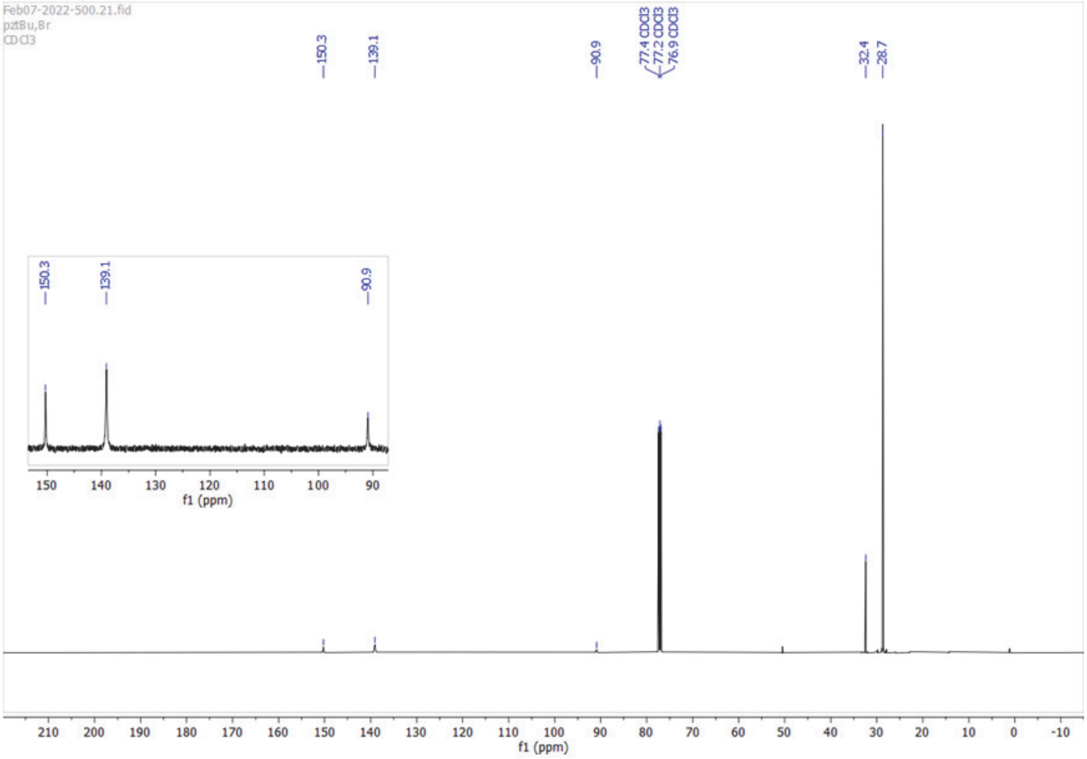

^{1}H ($CDCl_3$, 300 MHz) and ^{13}C{^{1}H} ($CDCl_3$, 75 MHz) NMR of **1c**

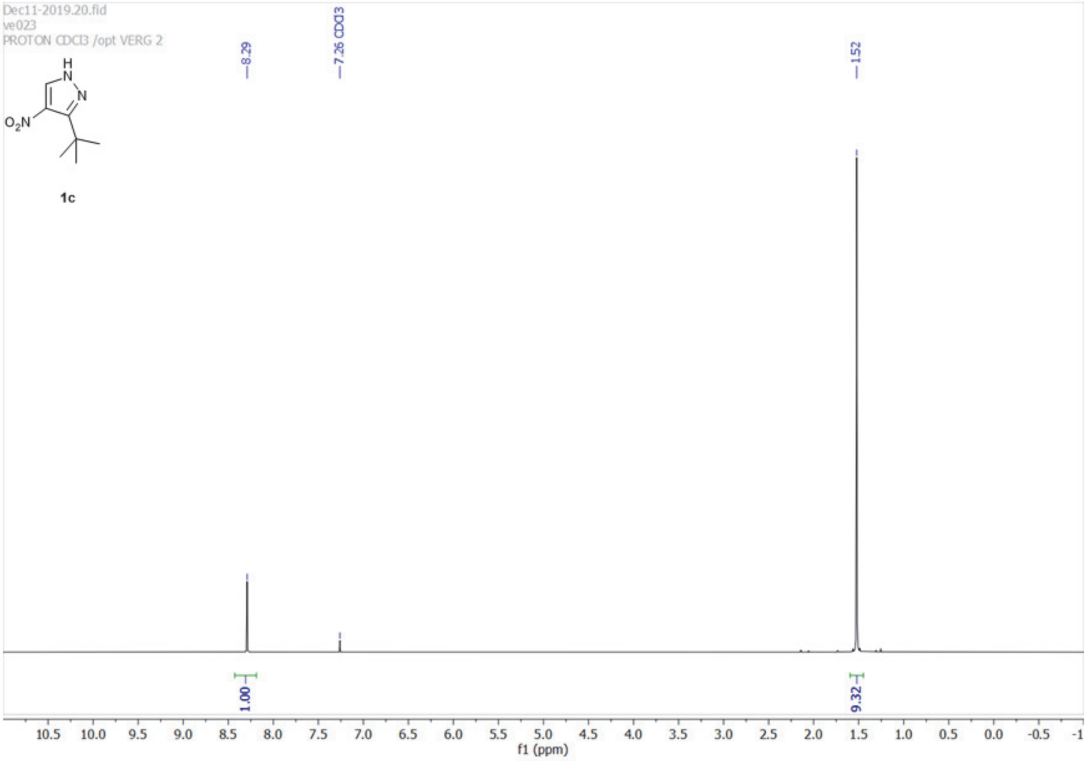

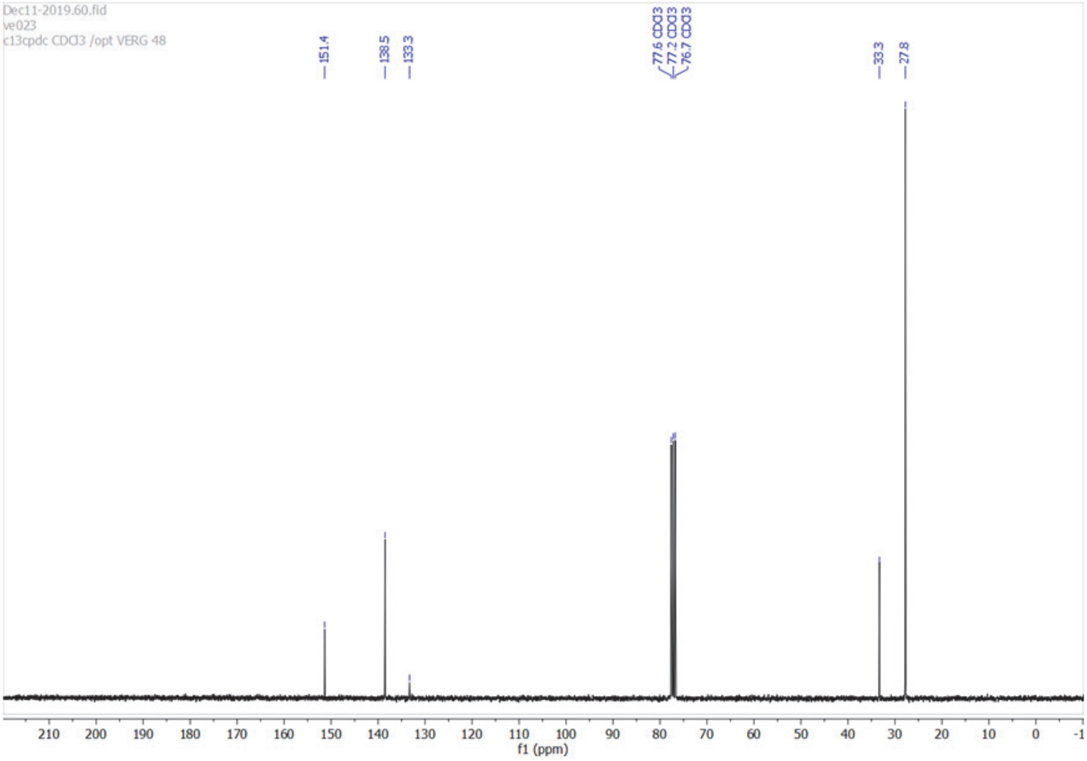

^1H (CD_3OD, 300 MHz) and $^{13}C\{^1H\}$ (CD_3OD, 75 MHz) NMR of **1d·H₂O**

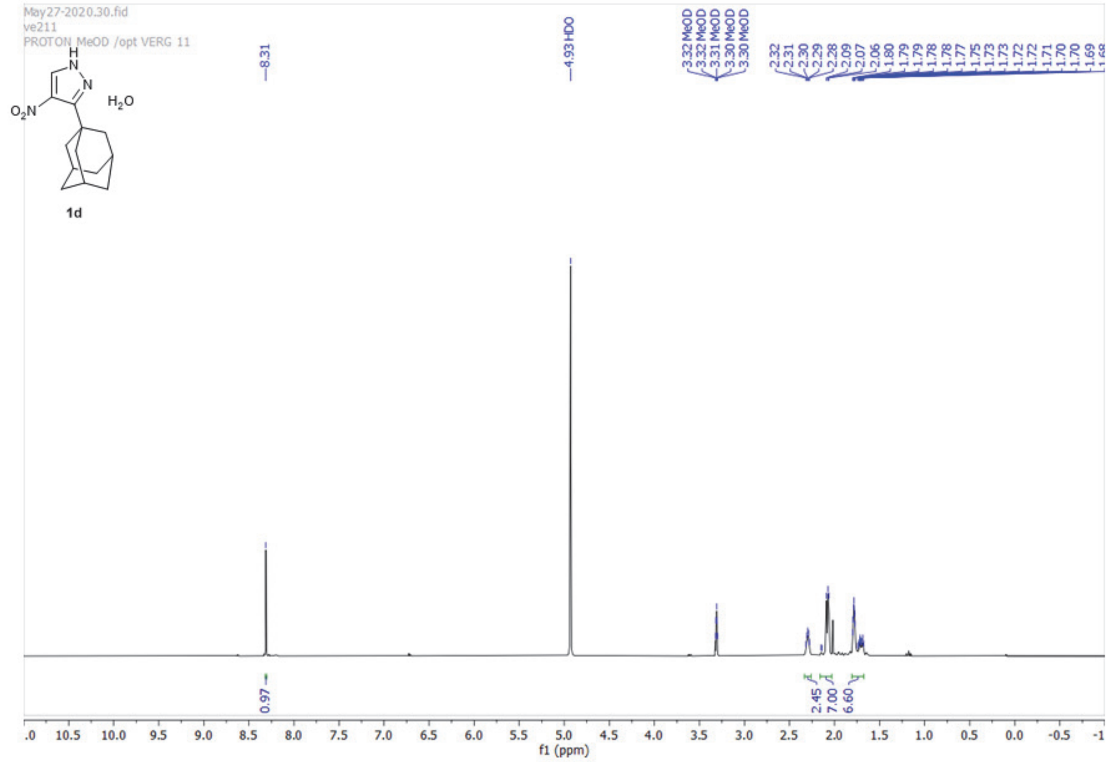

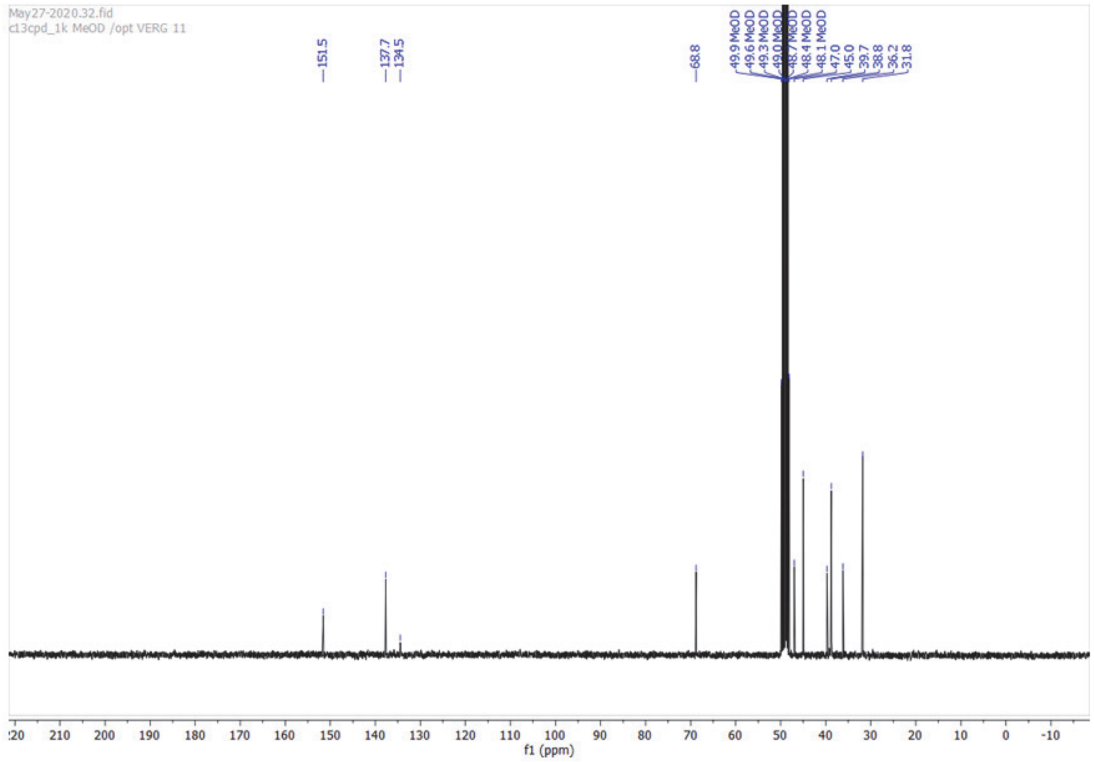

^{1}H ($CDCl_3$, 400 MHz) and $^{13}C\{^{1}H\}$ ($CDCl_3$, 100 MHz) NMR of **1e**

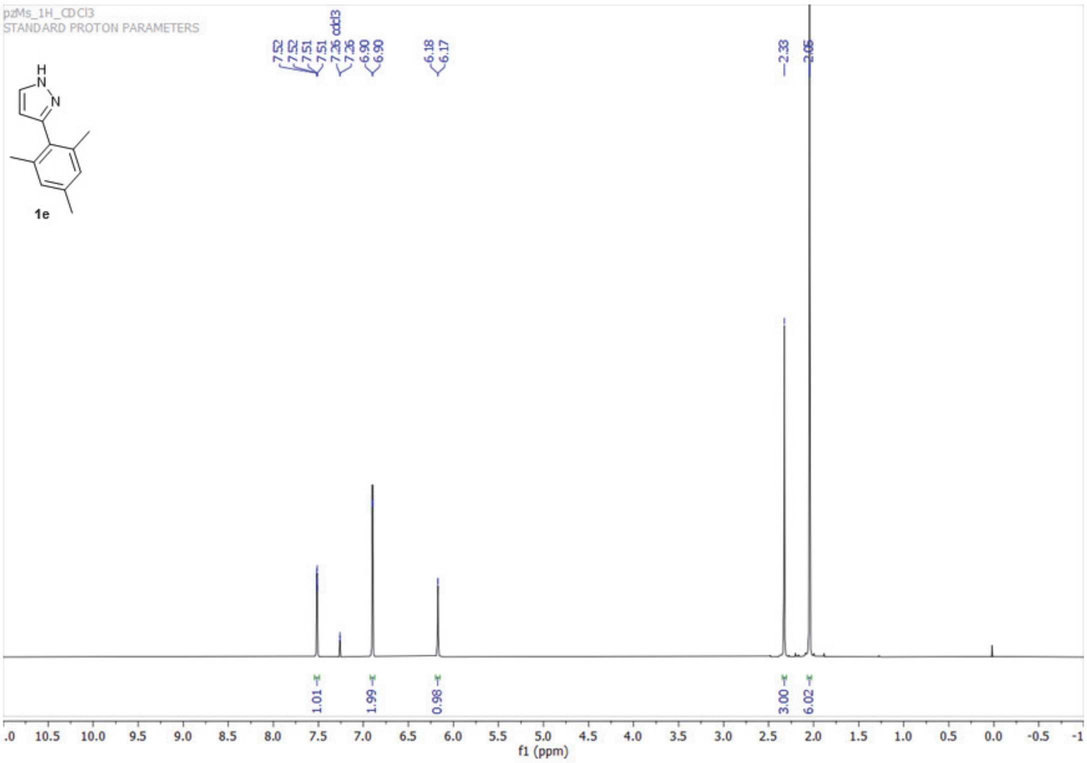

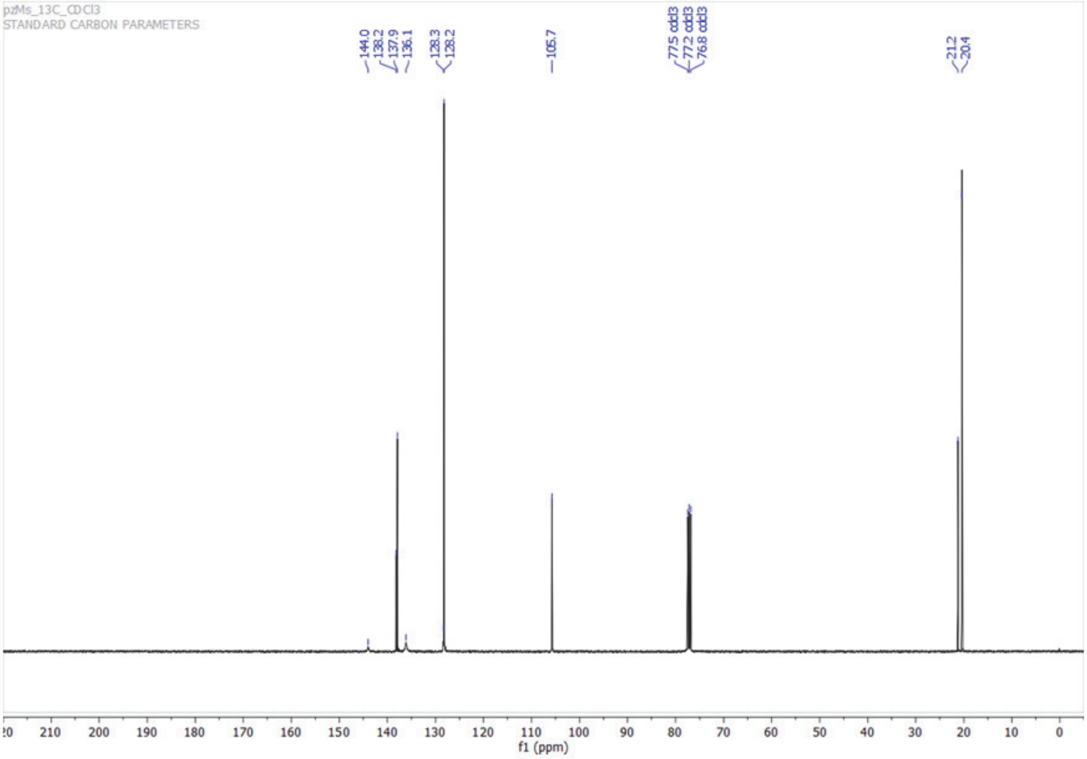

^{1}H ($CDCl_3$, 300 MHz) and $^{13}C\{^{1}H\}$ ($CDCl_3$, 75 MHz) NMR of **1f**

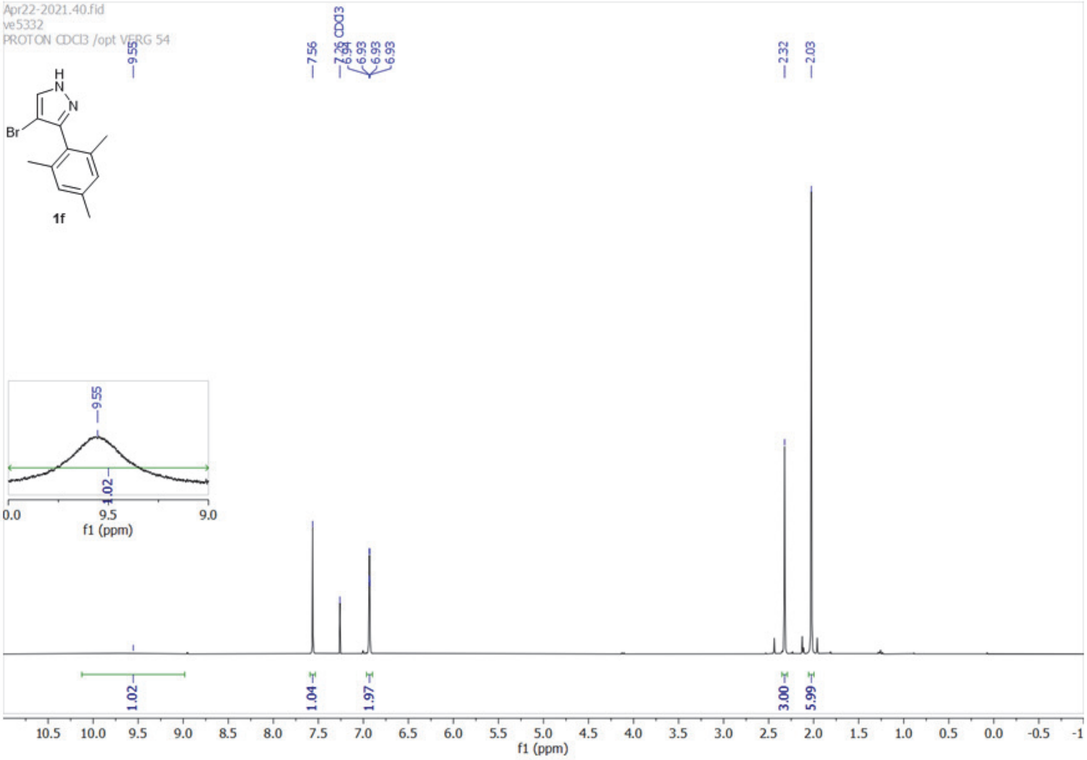

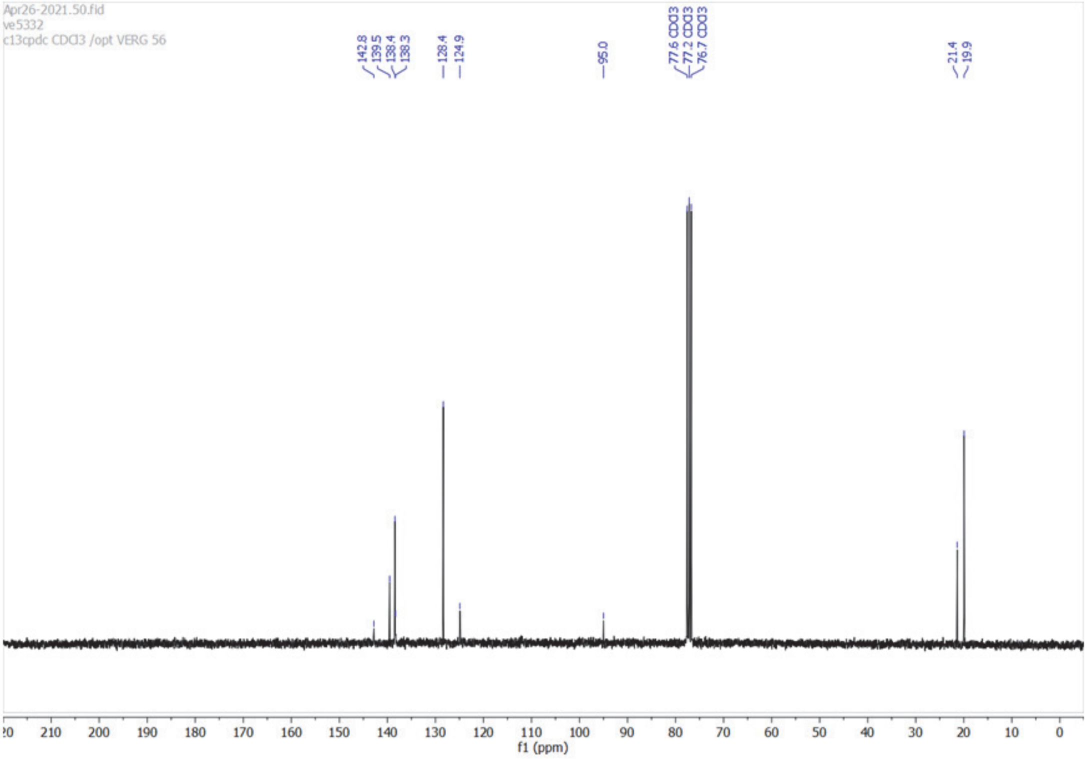

HRMS (+ESI) of **1f**:

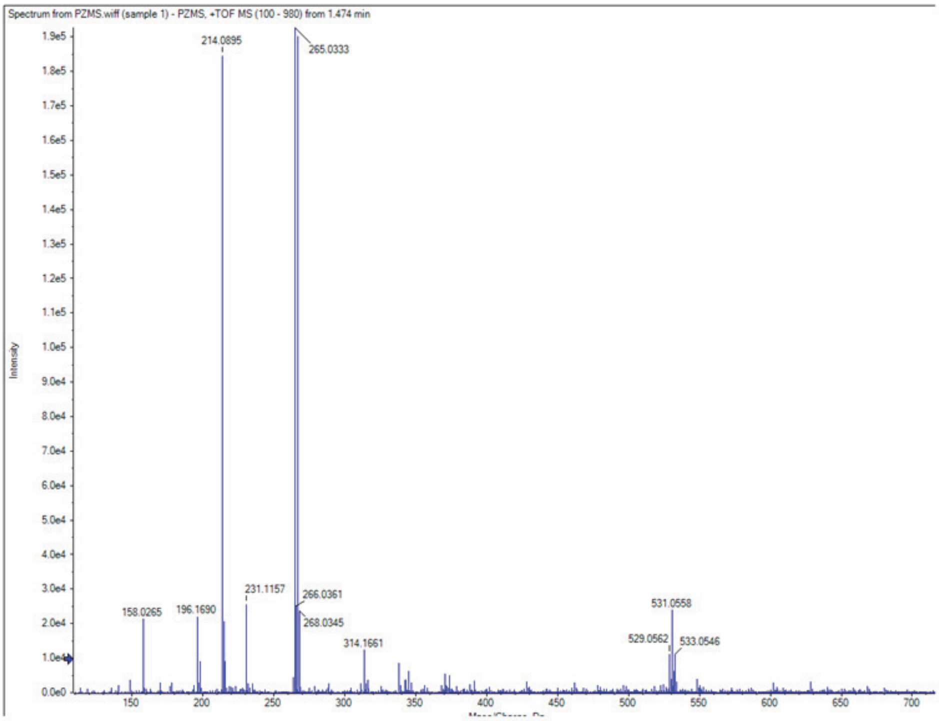

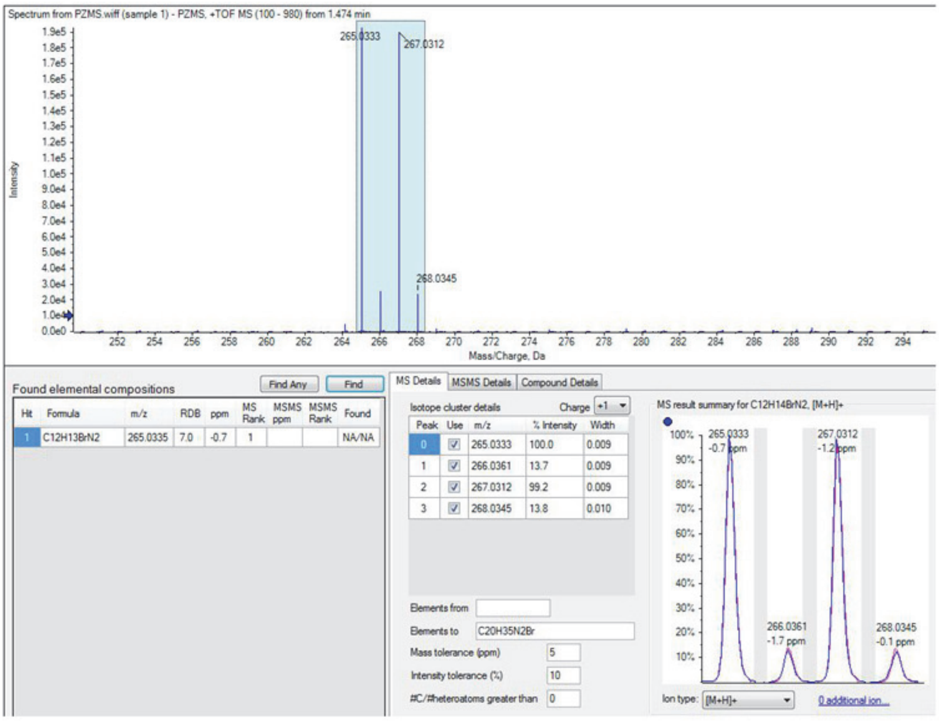

^1H (DMSO-d_6, 500 MHz) NMR of **1g**

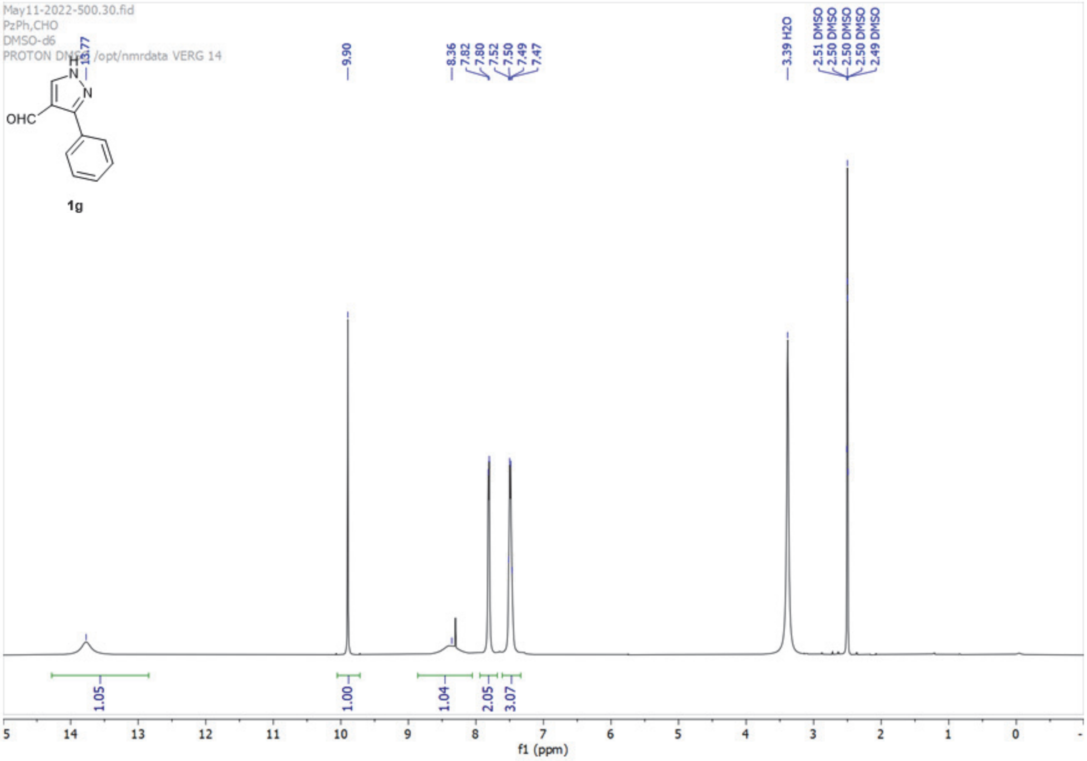

IR (ATR) spectrum of **1g**

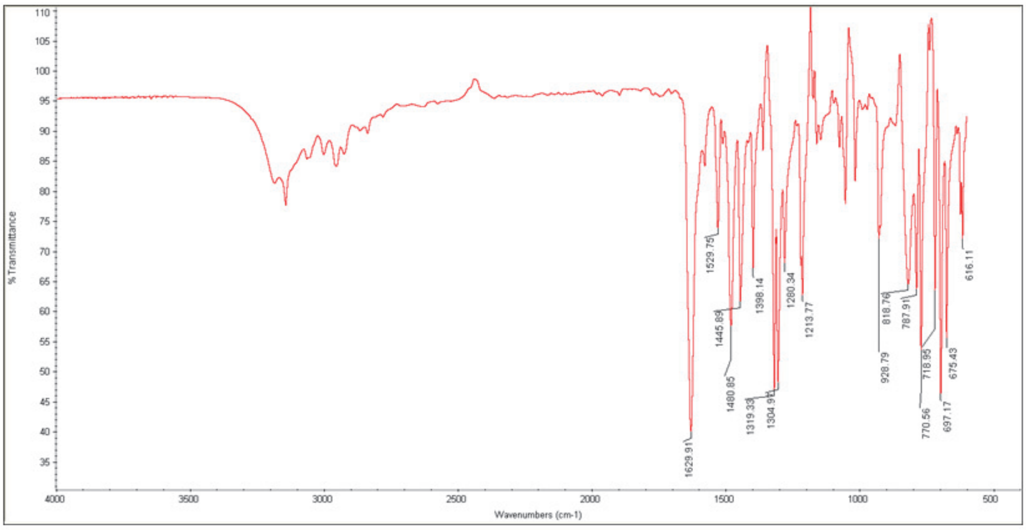

^1H ($CDCl_3$, 500 MHz) and $^{13}C\{^1H\}$ ($CDCl_3$, 125 MHz) NMR of **1h**

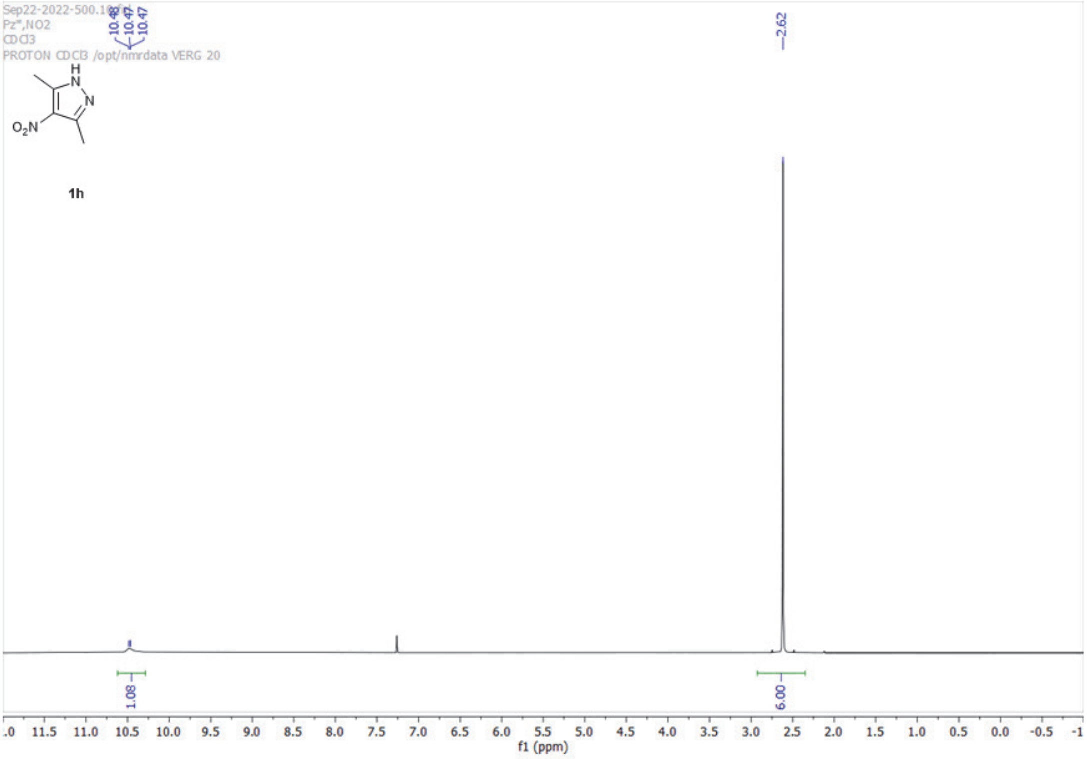

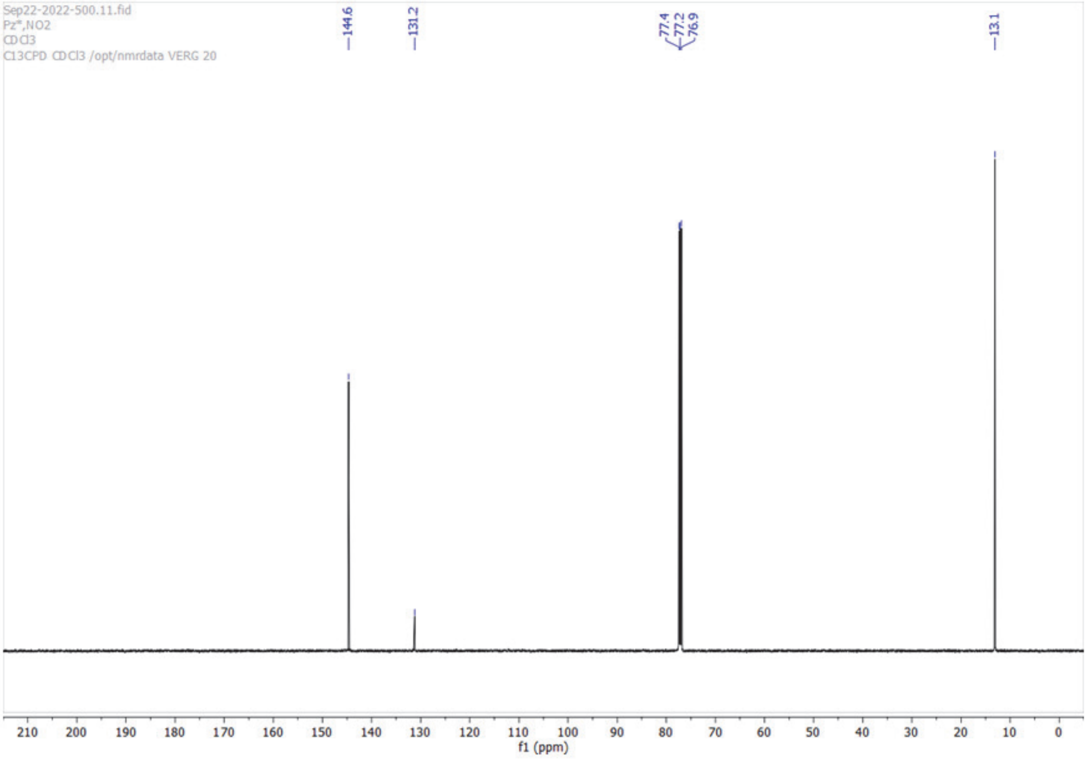

^{1}H ($CDCl_3$, 300 MHz) and $^{13}C\{^{1}H\}$ ($CDCl_3$, 75 MHz) NMR of **1i**

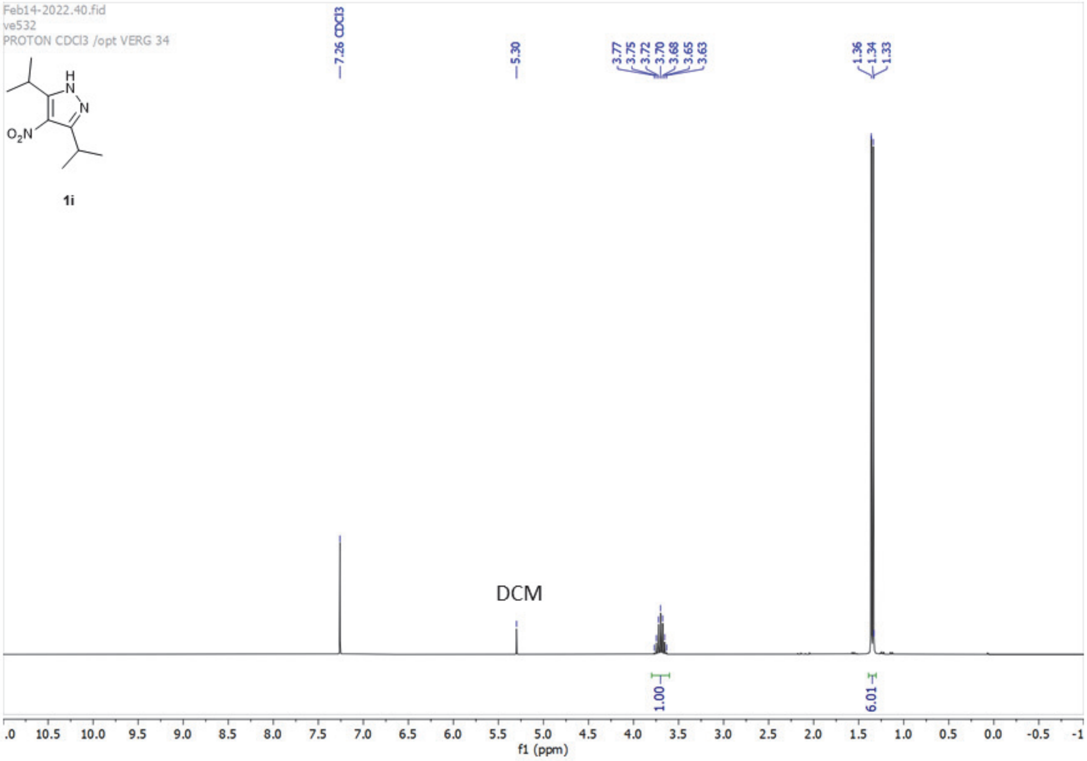

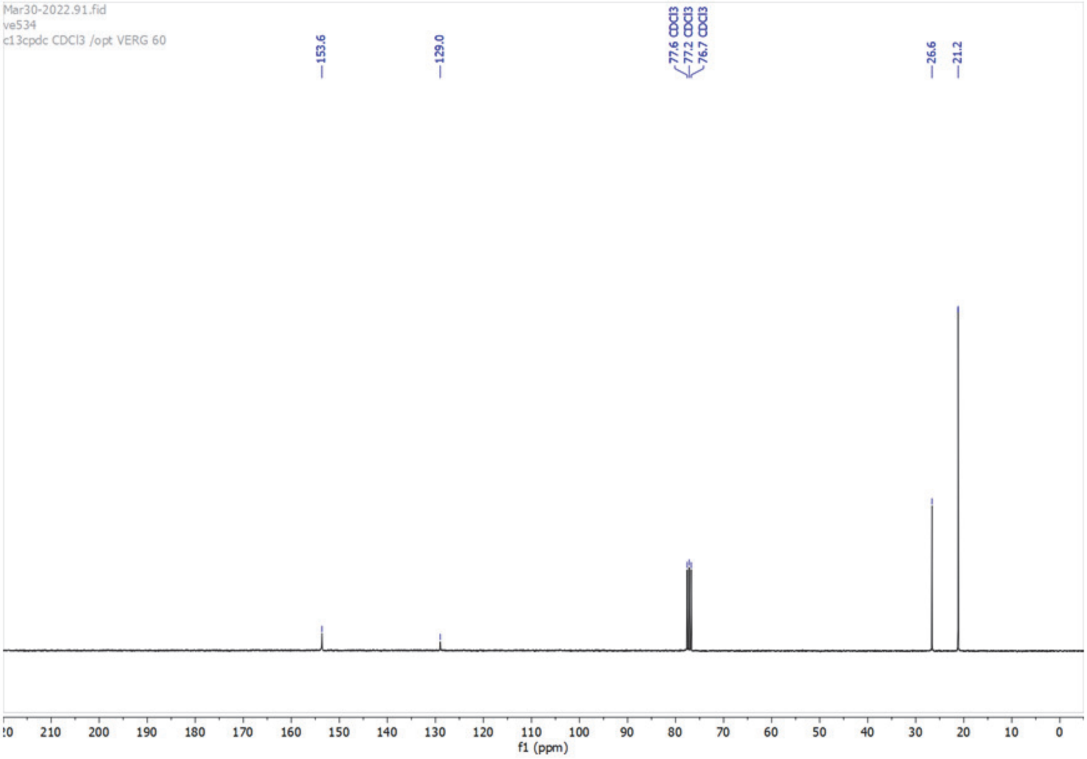

^1H ($CDCl_3$, 500 MHz) and ^{11}B ($CDCl_3$, 96 MHz) NMR of **$3a_{Tl}$**

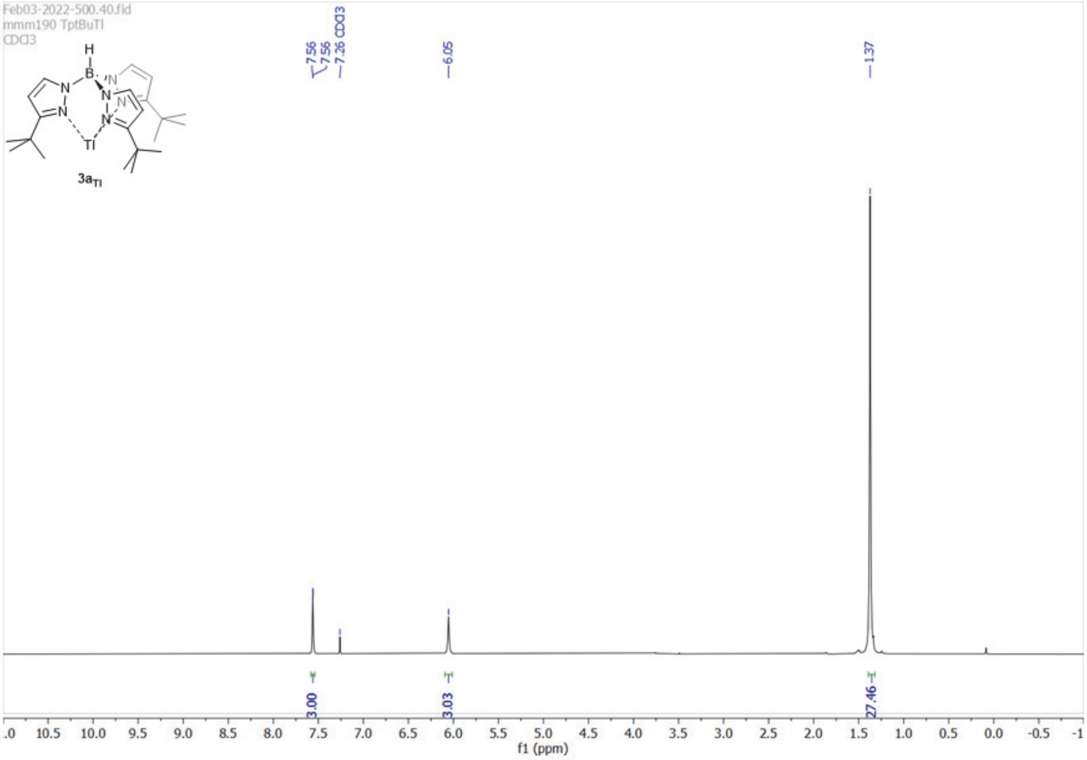

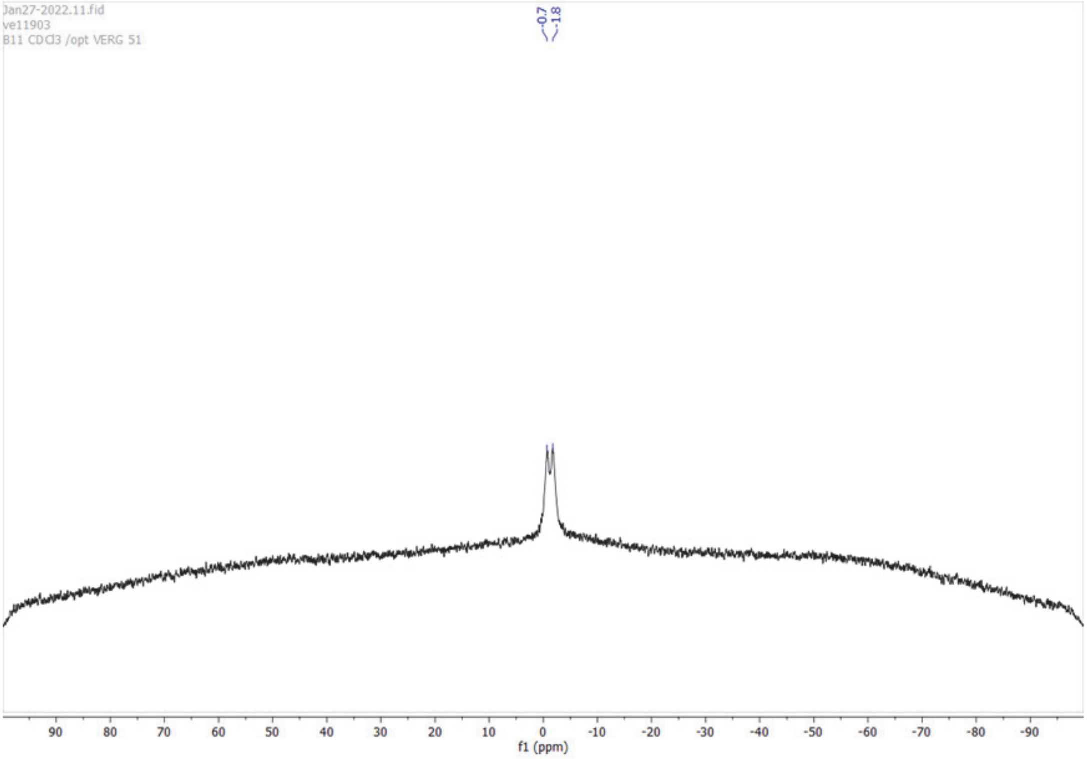

$^{13}C\{^1H\}$ ($CDCl_3$, 125 MHz) NMR of **3a$_{Tl}$**

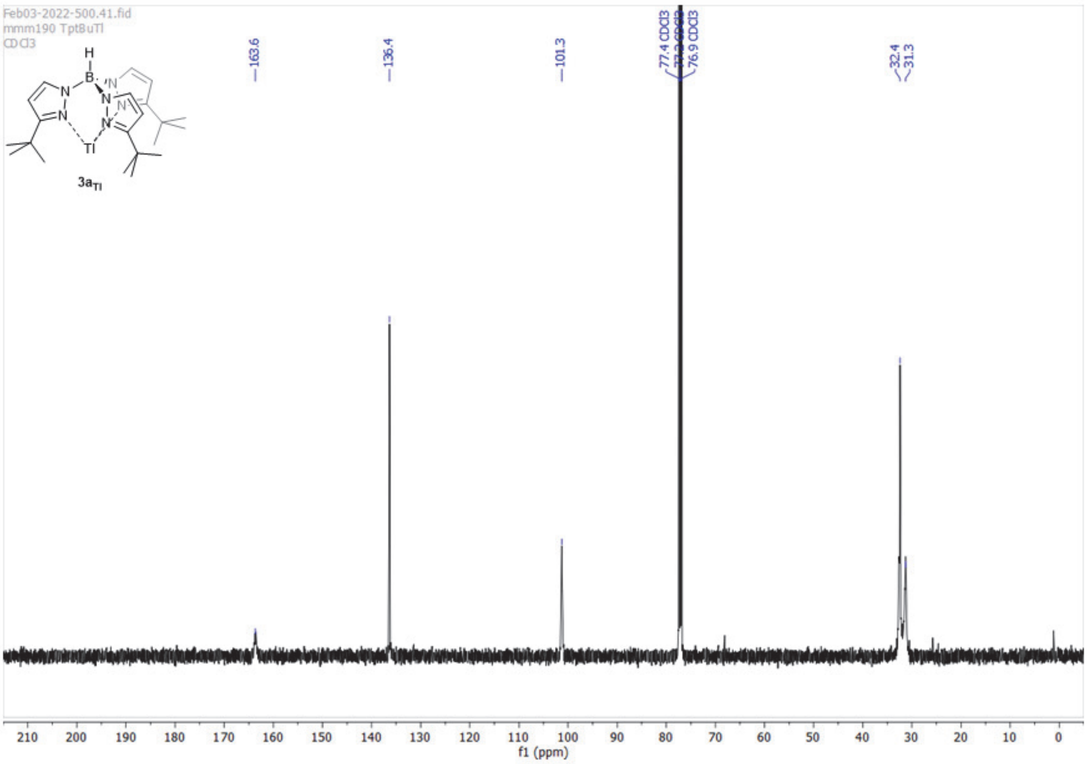

IR (ATR) spectrum of **3a$_{Tl}$**

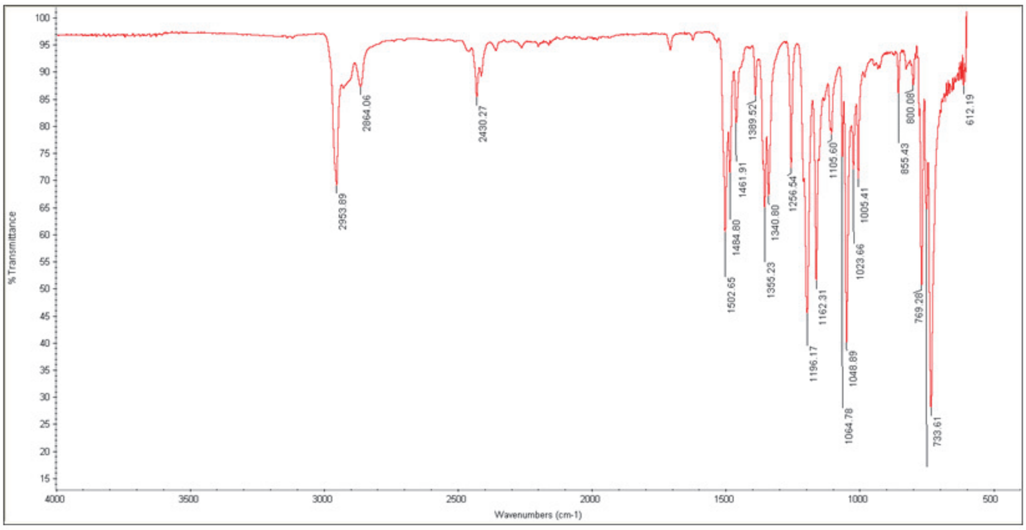

^{1}H ($CDCl_3$, 500 MHz) and ^{11}B ($CDCl_3$, 160 MHz) NMR of **$3b_{Tl}$**

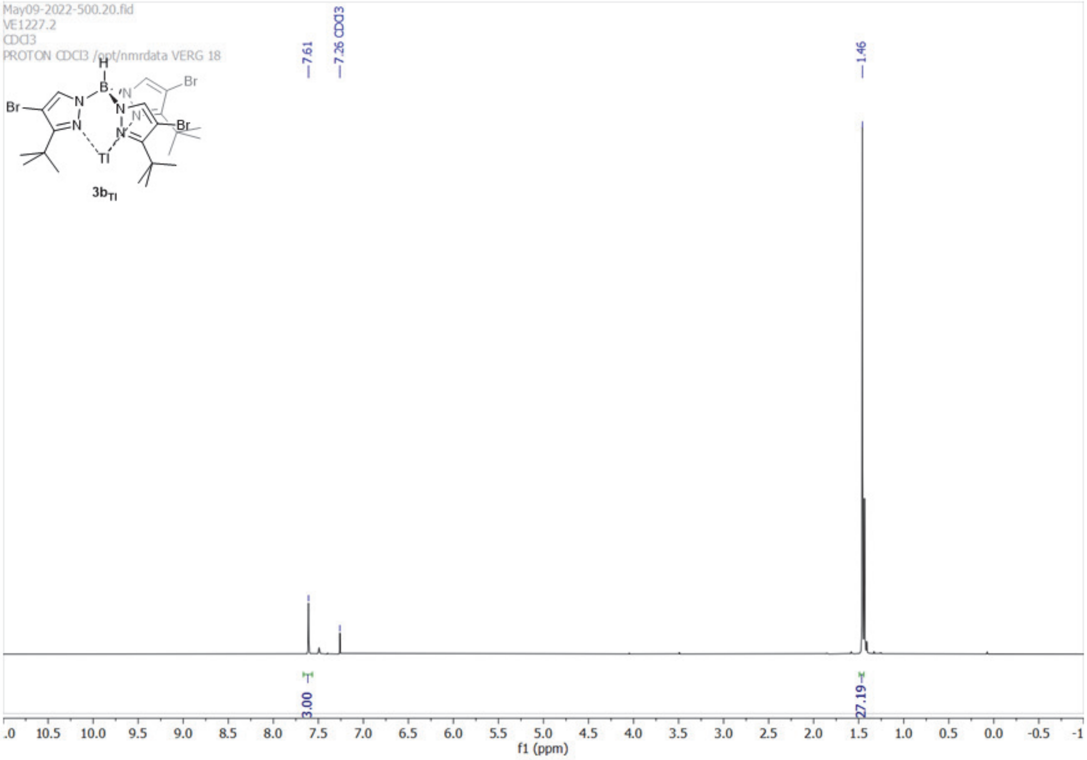

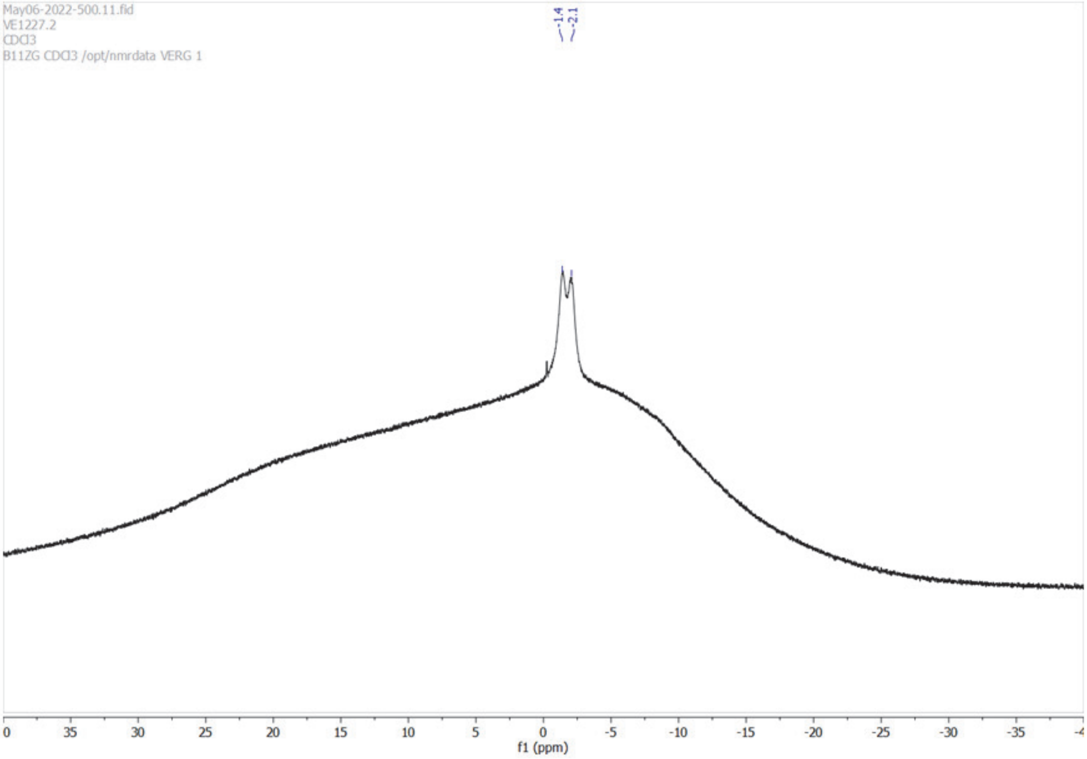

$^{13}C\{^{1}H\}$ ($CDCl_3$, 125 MHz) NMR of **$3b_{Tl}$**

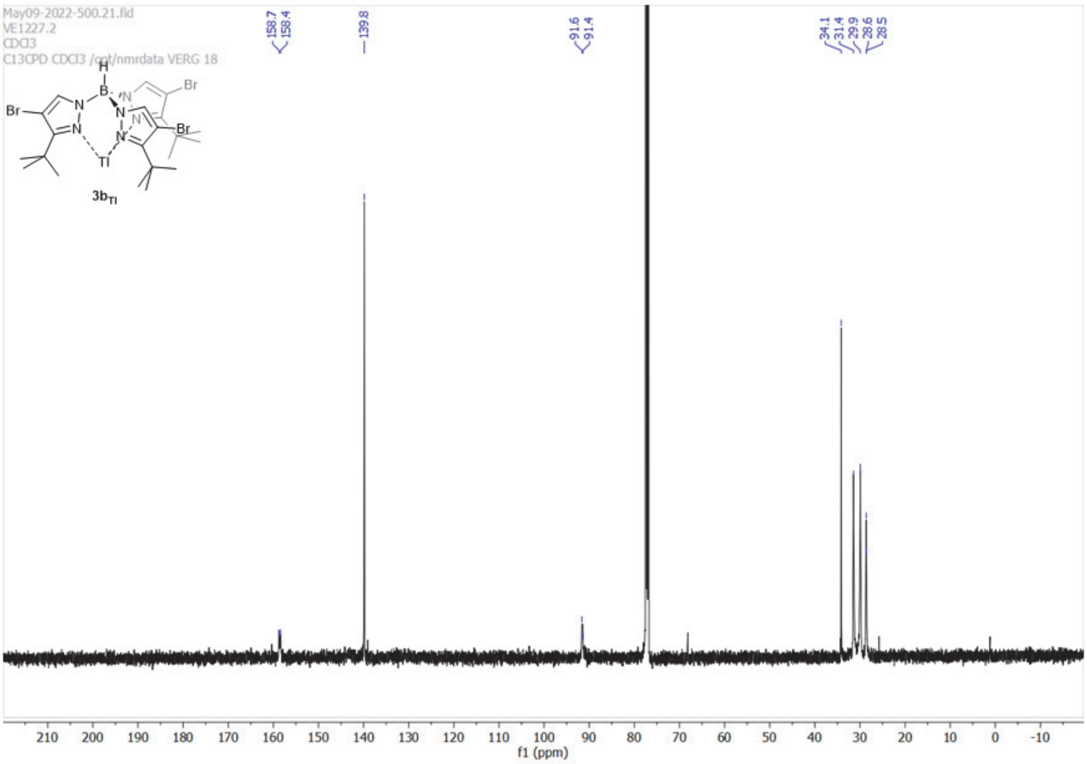

IR (ATR) spectrum of **$3b_{Tl}$**

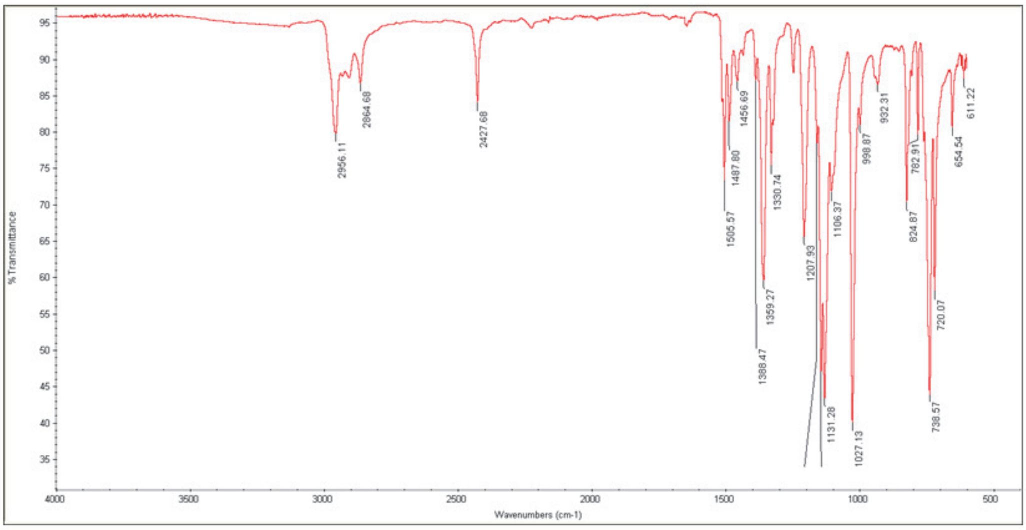

^{1}H (acetone-d_6, 500 MHz) and ^{11}B (acetone-d_6, 160 MHz) NMR of **$3c_{Tl}$·DCM**

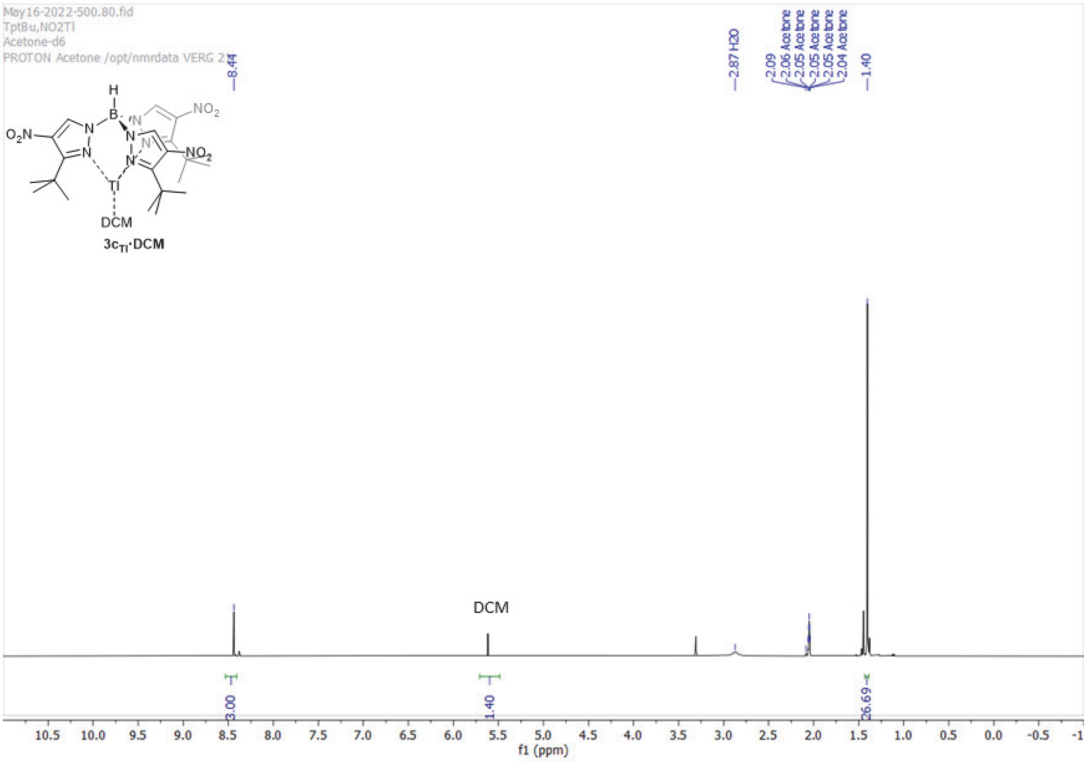

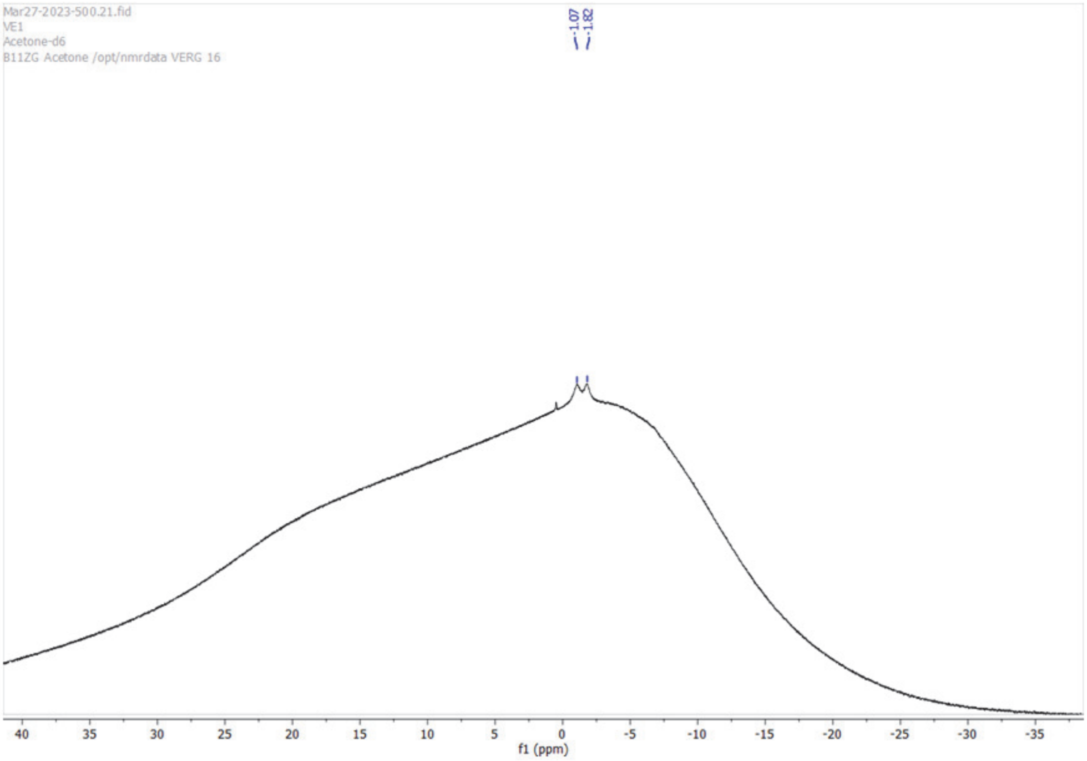

$^{13}C\{^{1}H\}$ (acetone-d_6, 125 MHz) NMR of **$3c_{Tl}$·DCM**

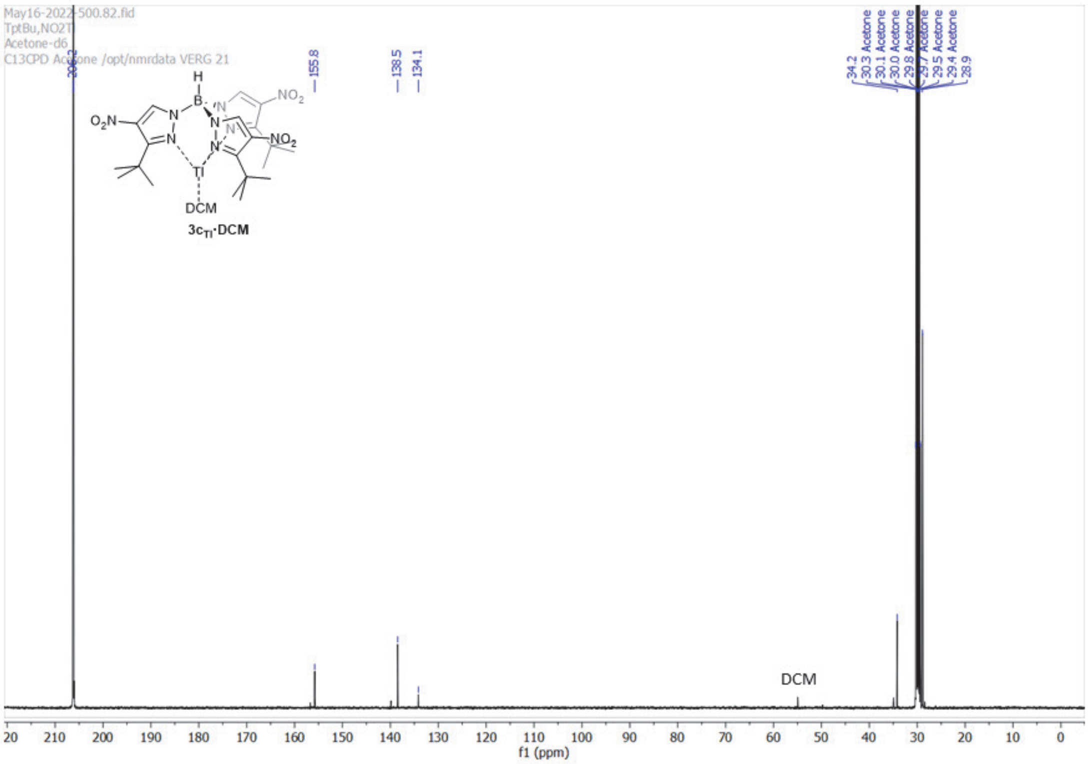

IR (ATR) spectrum of **$3c_{Tl}$**

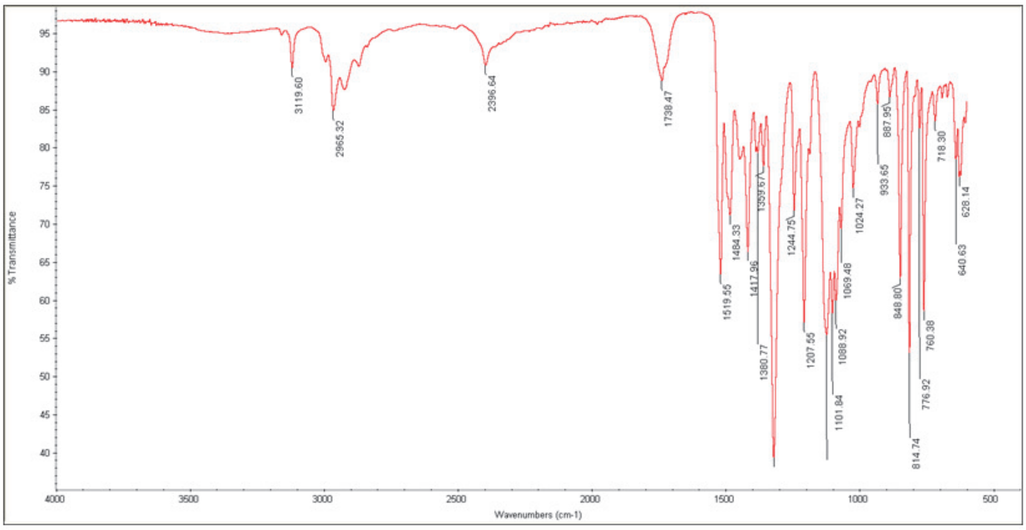

^{1}H ($CDCl_3$, 300 MHz) and ^{11}B (acetone-d_6, 160 MHz) NMR of **$3d_{Tl}$·3CHCl$_3$**

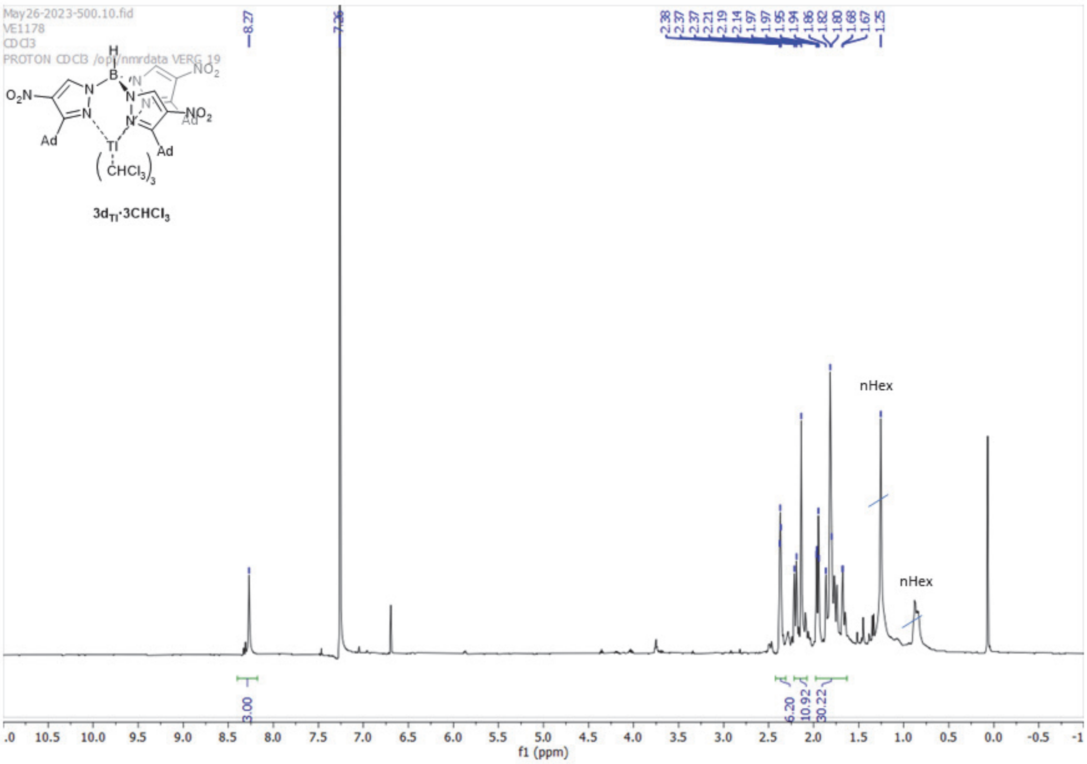

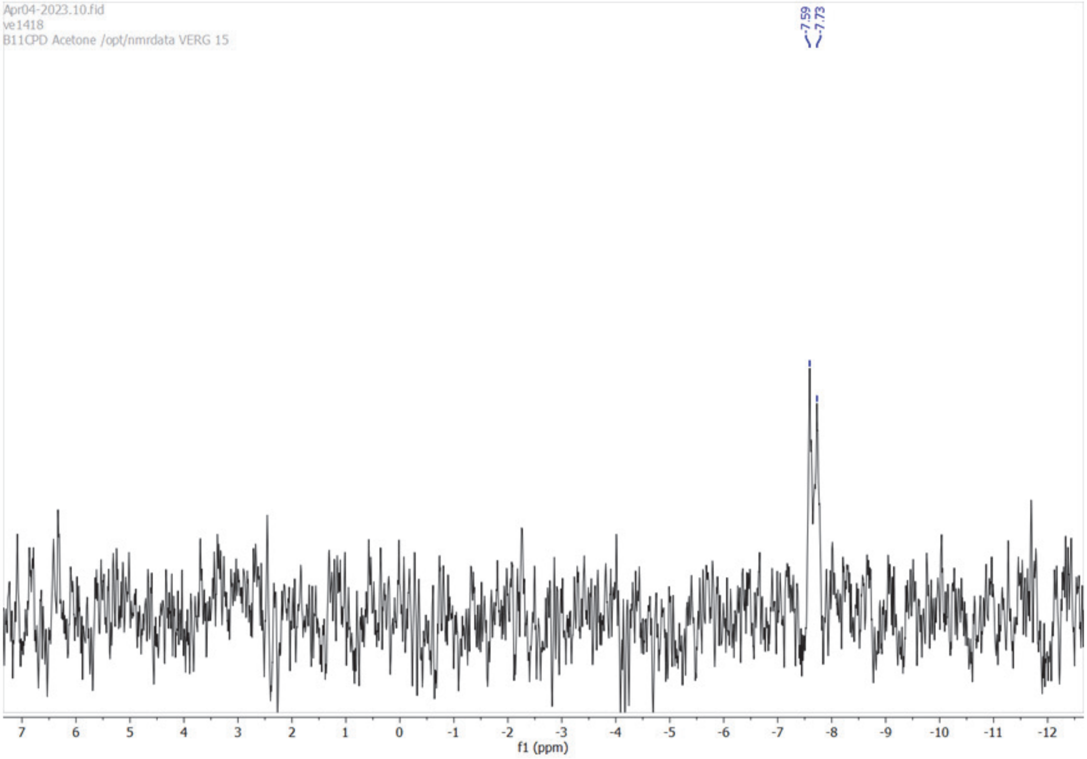

$^{13}C\{^1H\}$ ($CDCl_3$, 125 MHz) NMR of **$3d_{Tl}$·3CHCl$_3$**

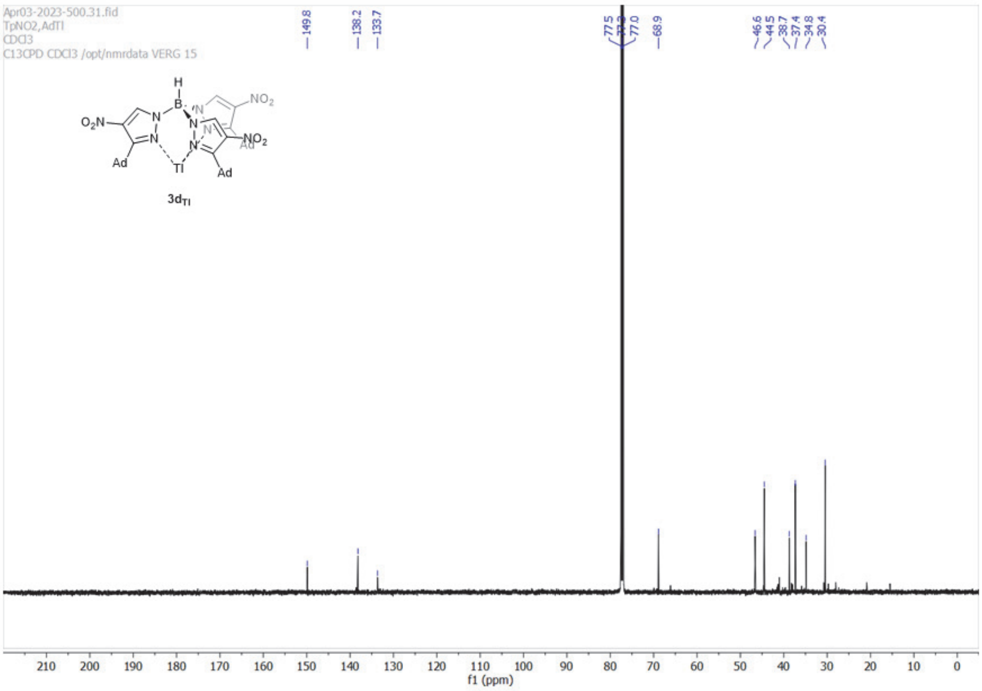

IR (ATR) spectrum of **$3d_{Tl}$·3CHCl$_3$**

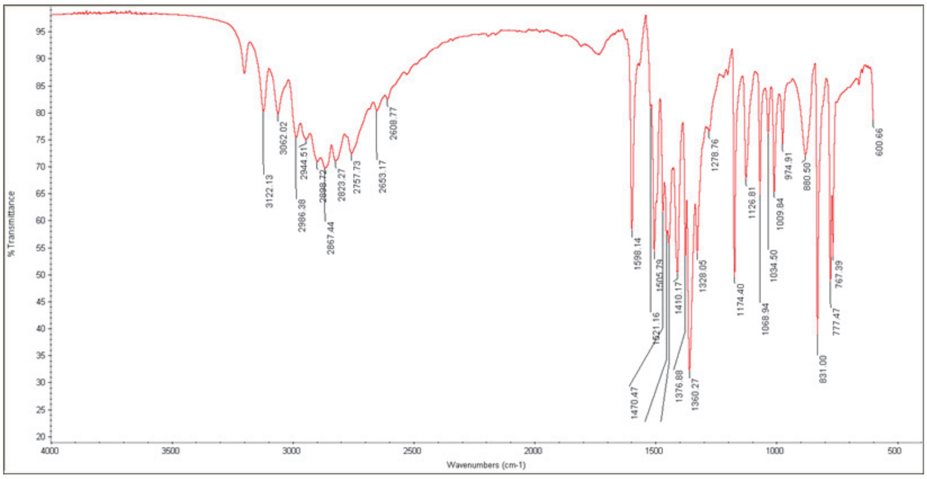

^{1}H (CD_2Cl_2, 500 MHz) and ^{11}B (CD_2Cl_2, 160 MHz) NMR of **$3e_{Tl}$**

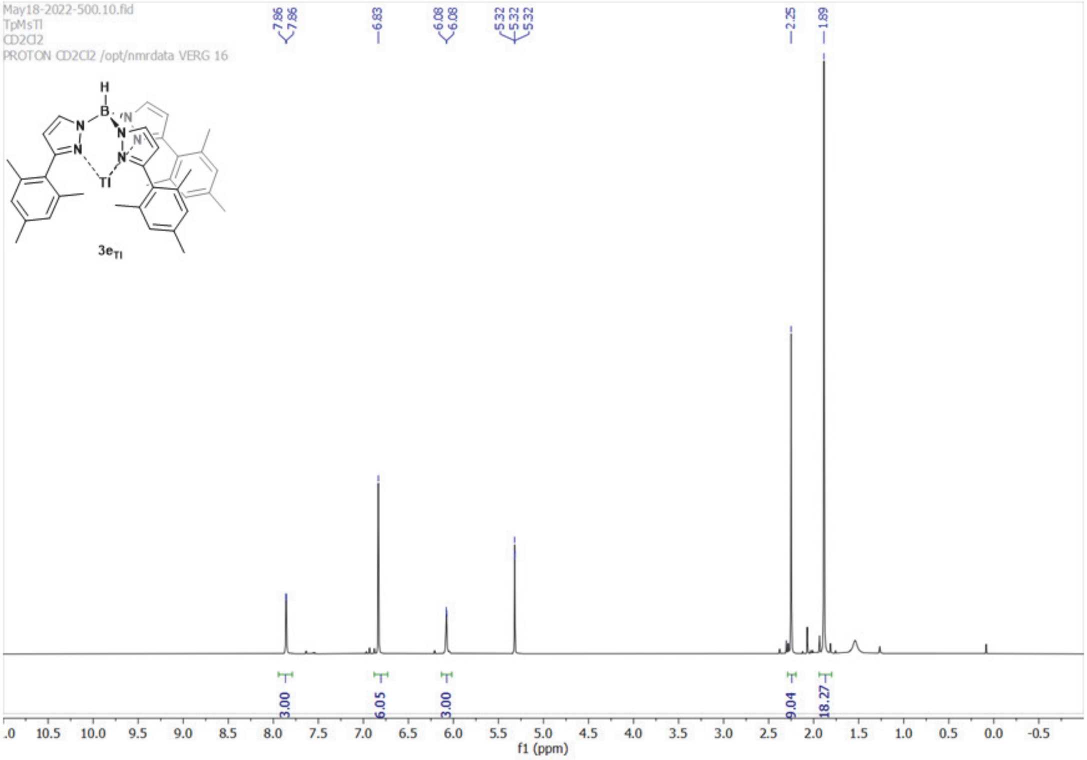

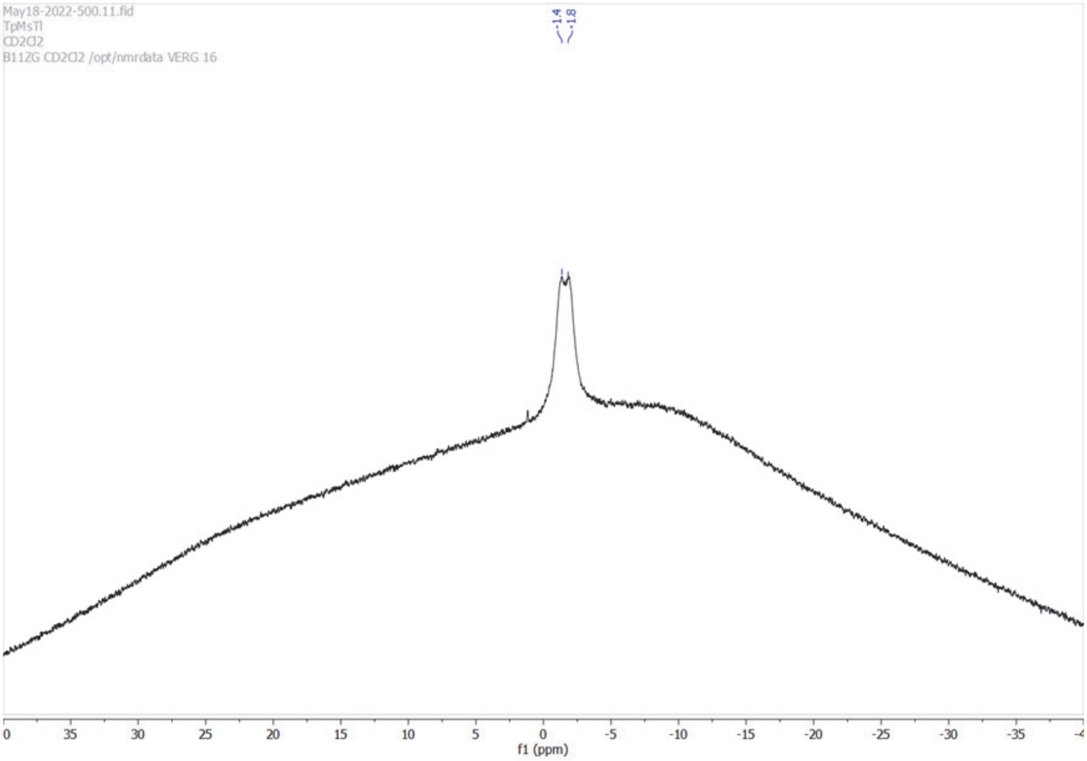

$^{13}C\{^{1}H\}$ (CD_2Cl_2, 125 MHz) NMR of **3e$_{Tl}$**

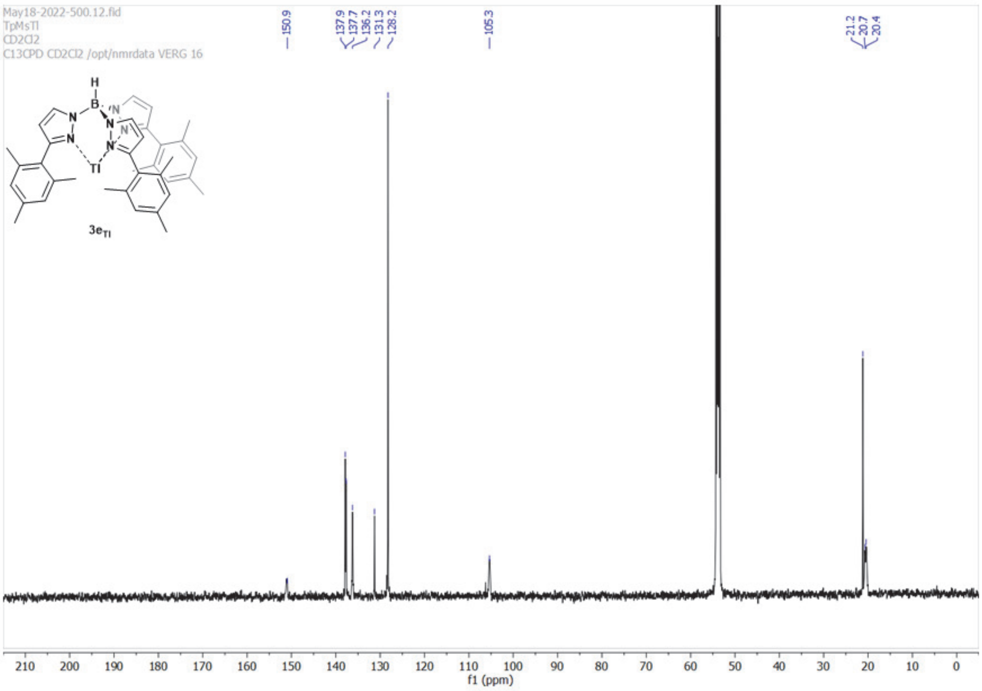

IR (ATR) spectrum of **3e$_{Tl}$**

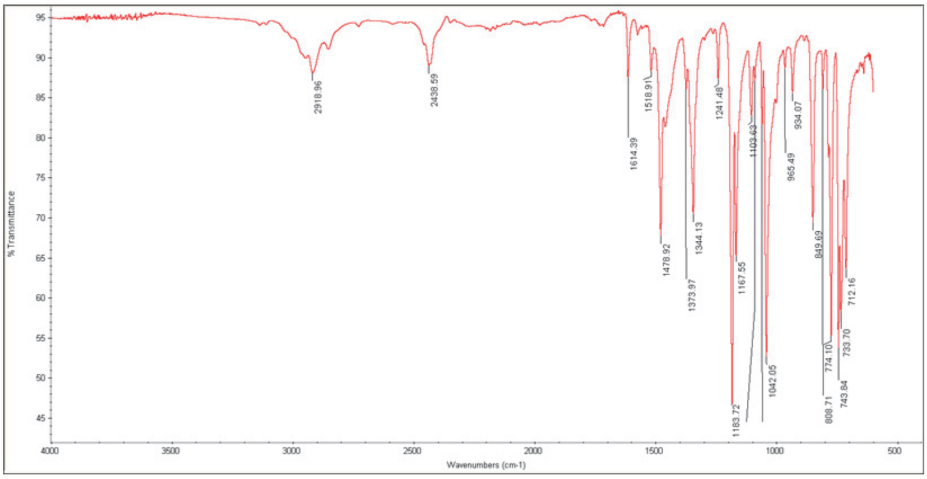

^{1}H (CD$_2$Cl$_2$, 500 MHz) and ^{11}B (CD$_2$Cl$_2$, 160 MHz) NMR of **3f$_{Tl}$·1/5THF**

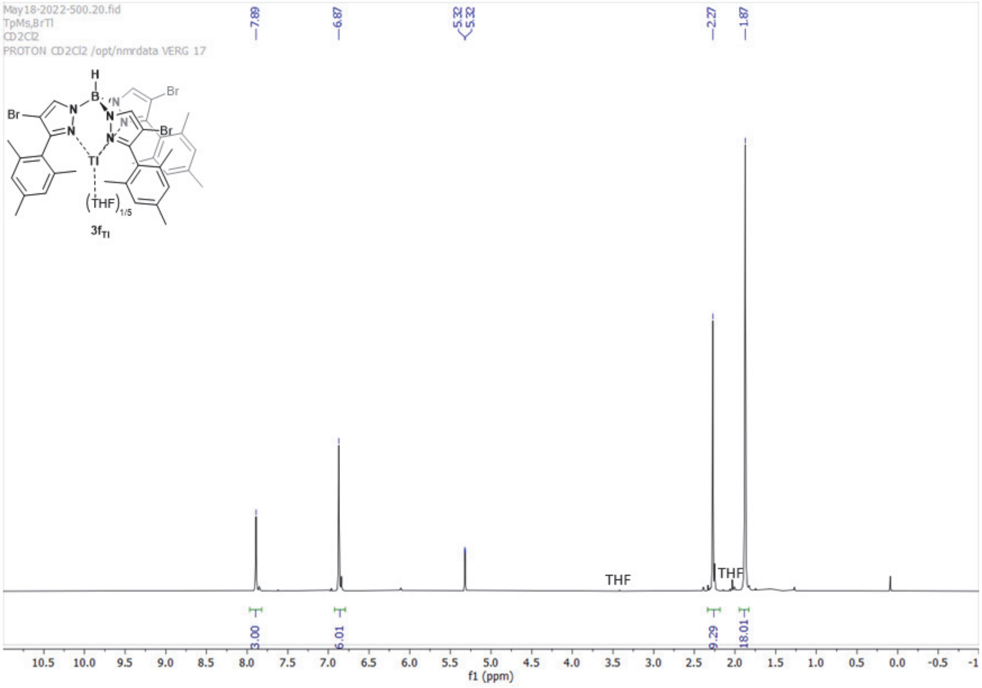

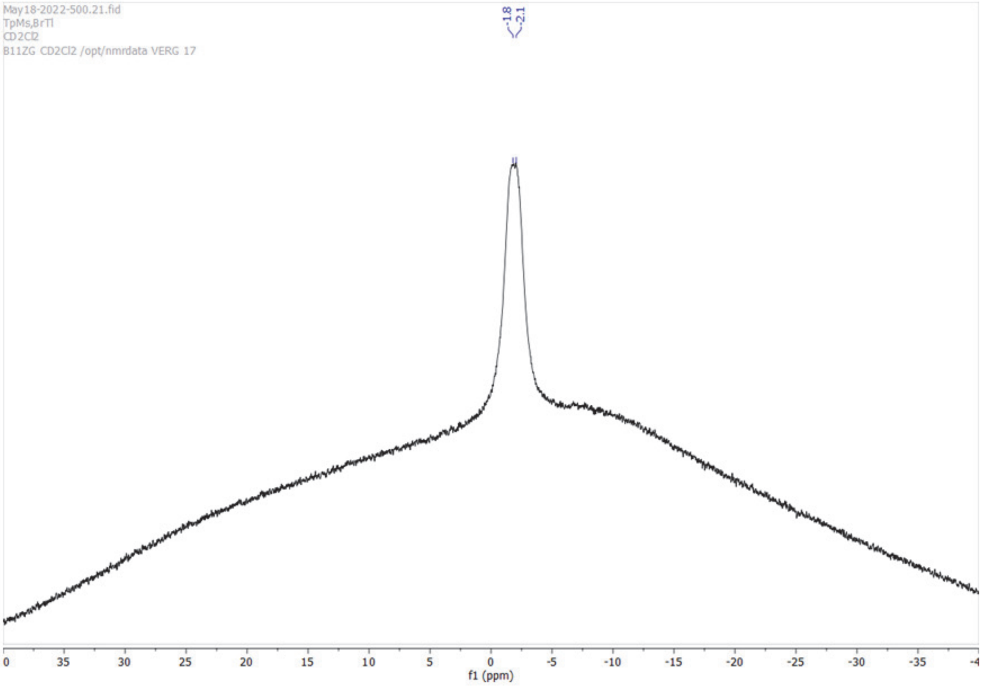

$^{13}C\{^1H\}$ (CD_2Cl_2, 125 MHz) NMR of **$3f_{Tl}$·1/5THF**

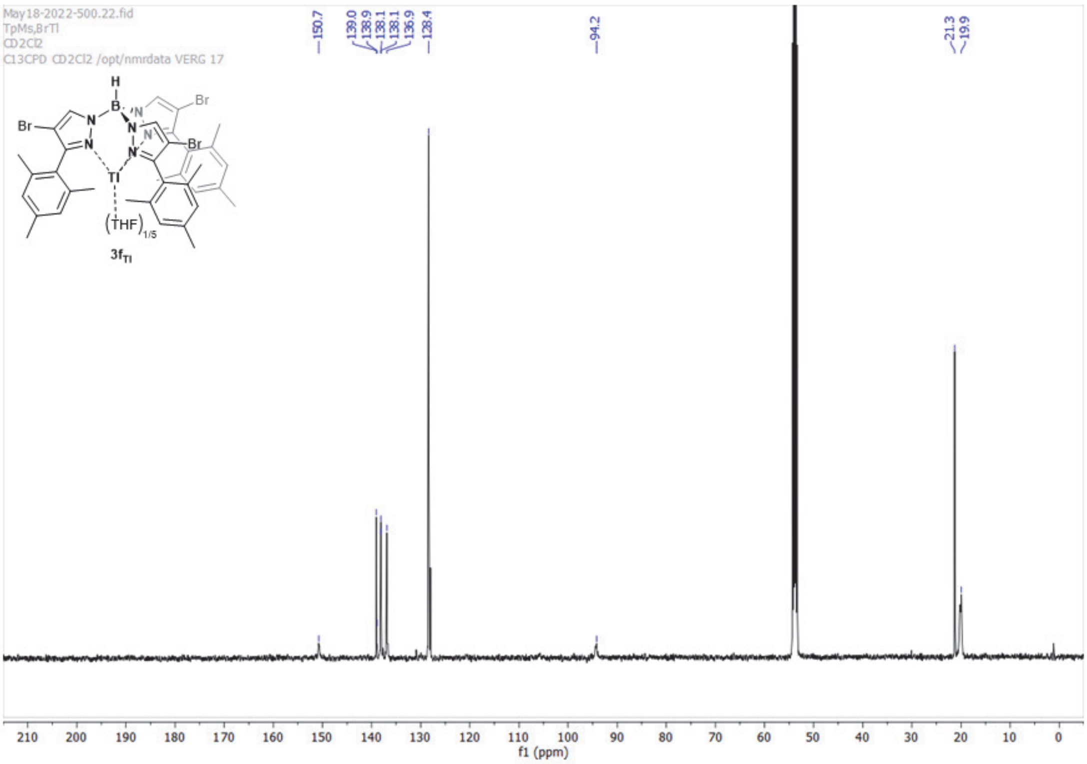

IR (ATR) spectrum of **$3f_{Tl}$**

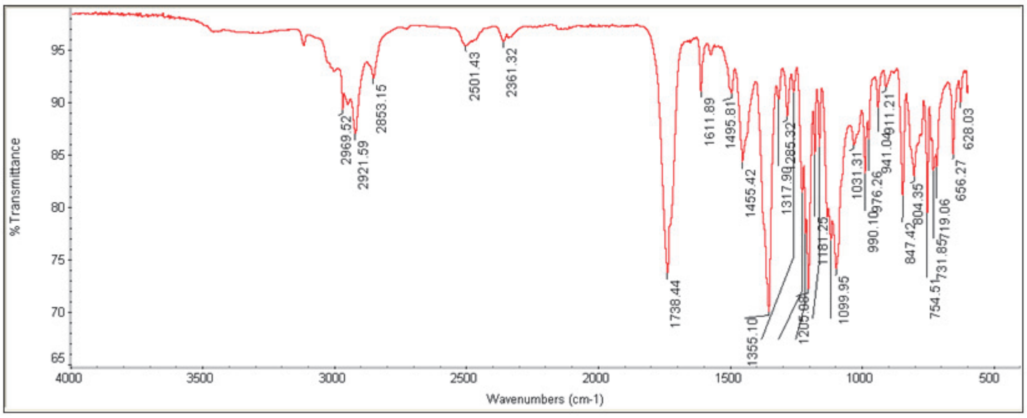

^{1}H ($CDCl_3$, 300 MHz) and ^{11}B ($CDCl_3$, 96 MHz) NMR of **$3g_{Na}$·$3H_2O$**

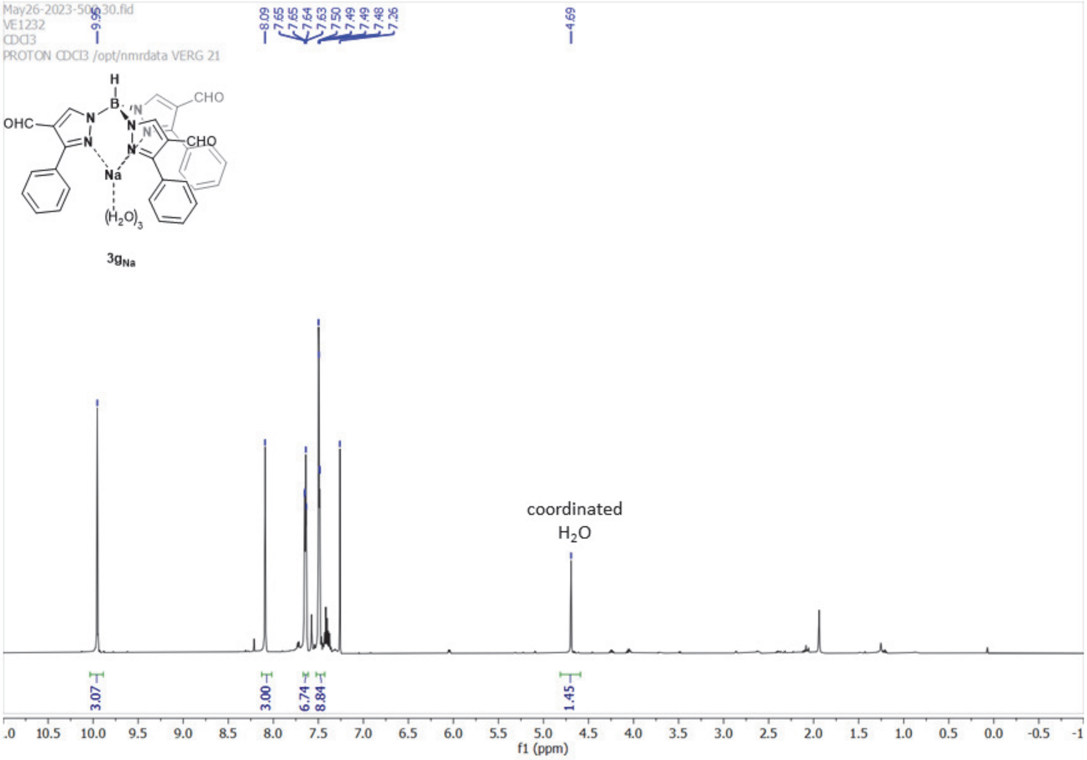

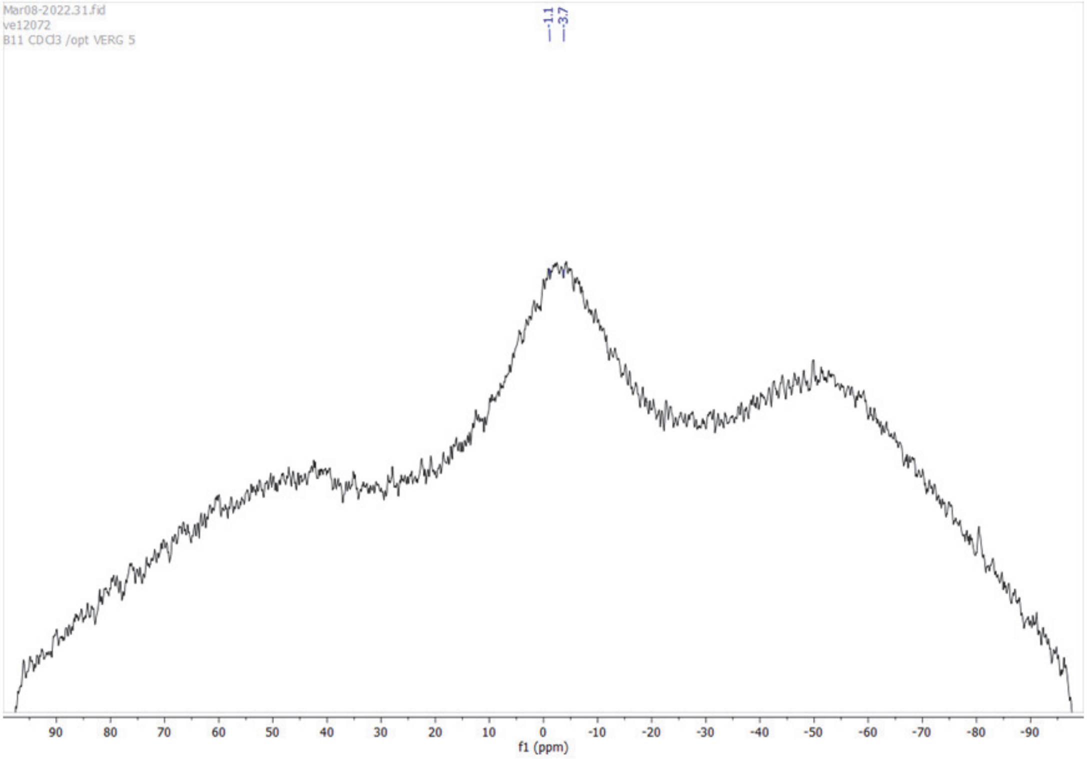

$^{13}C\{^1H\}$ $CDCl_3$, 125 MHz) NMR of $\mathbf{3g_{Na}\cdot 3H_2O}$

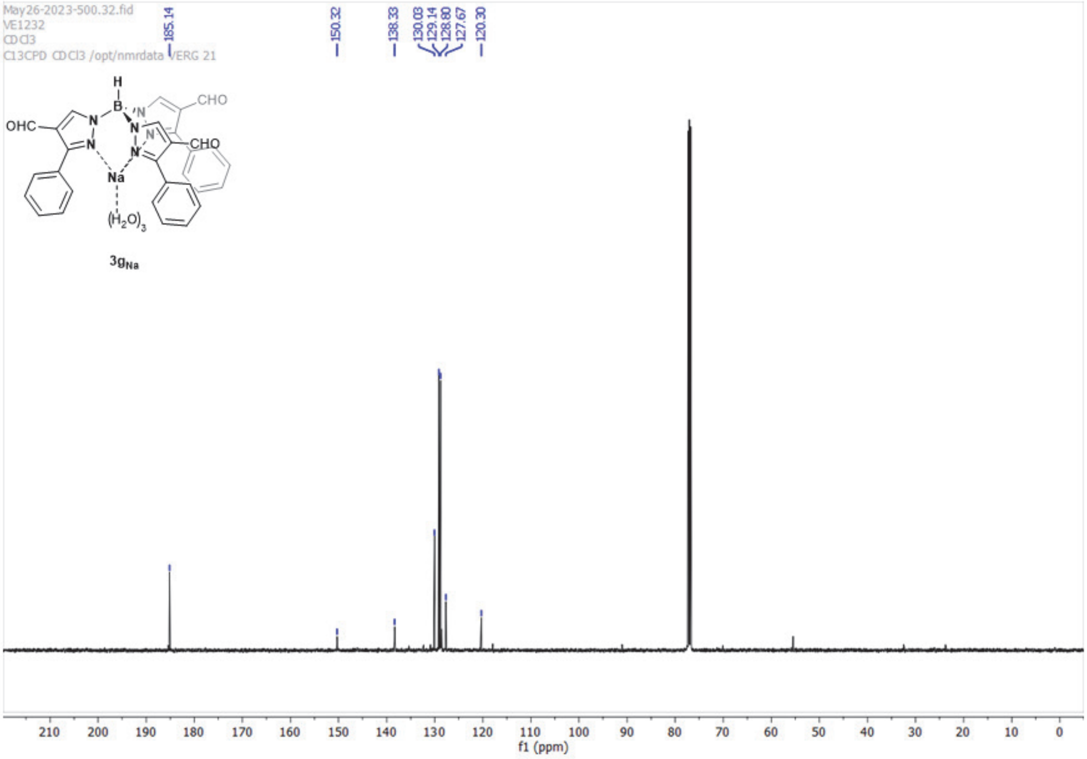

IR (ATR) spectrum of $\mathbf{3g_{Na}}$

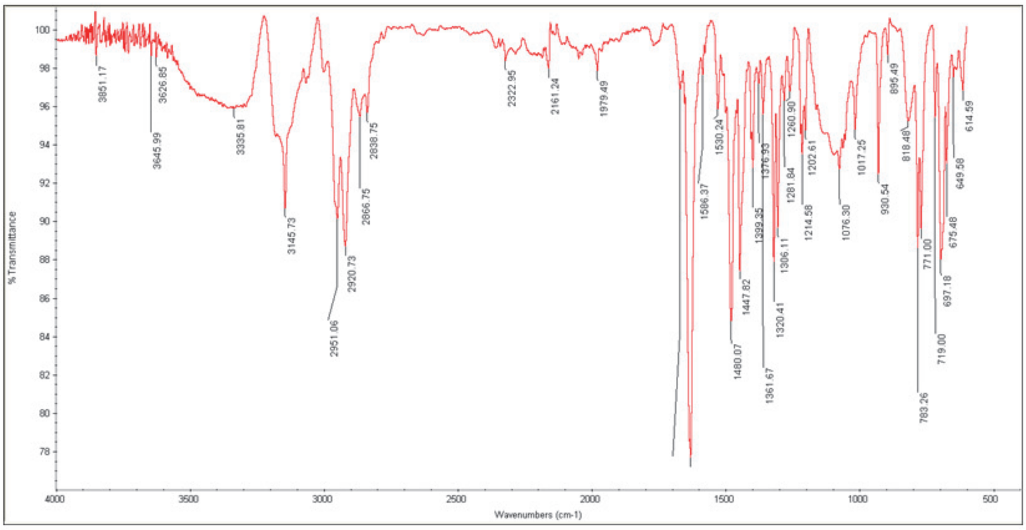

^{1}H ($CDCl_3$, 500 MHz) and ^{11}B ($CDCl_3$, 96 MHz) NMR of **$3h_{Na}$·DCM**

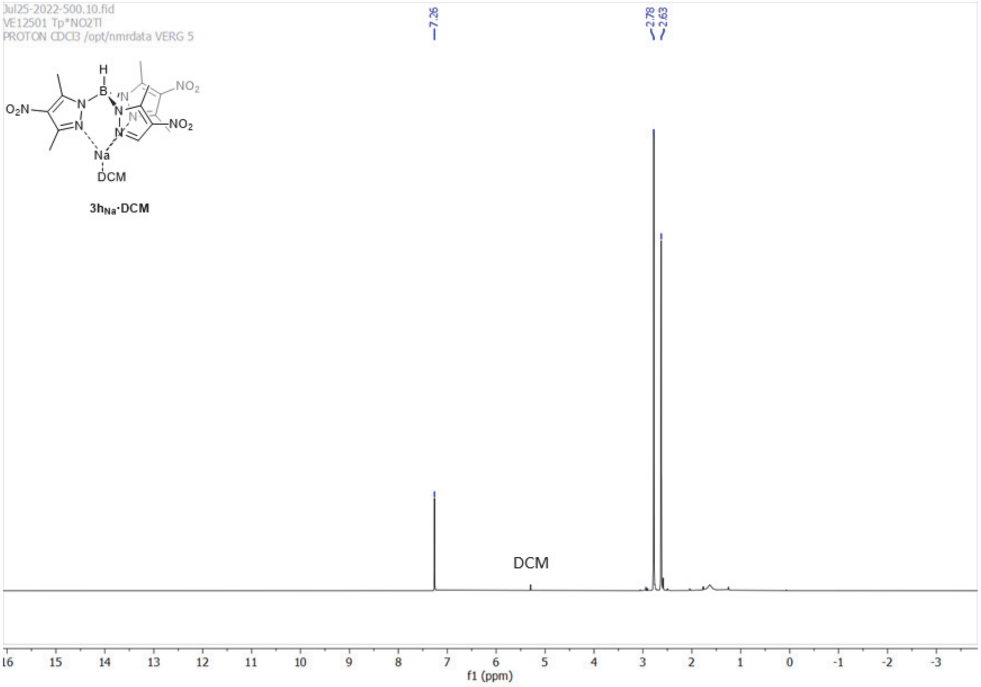

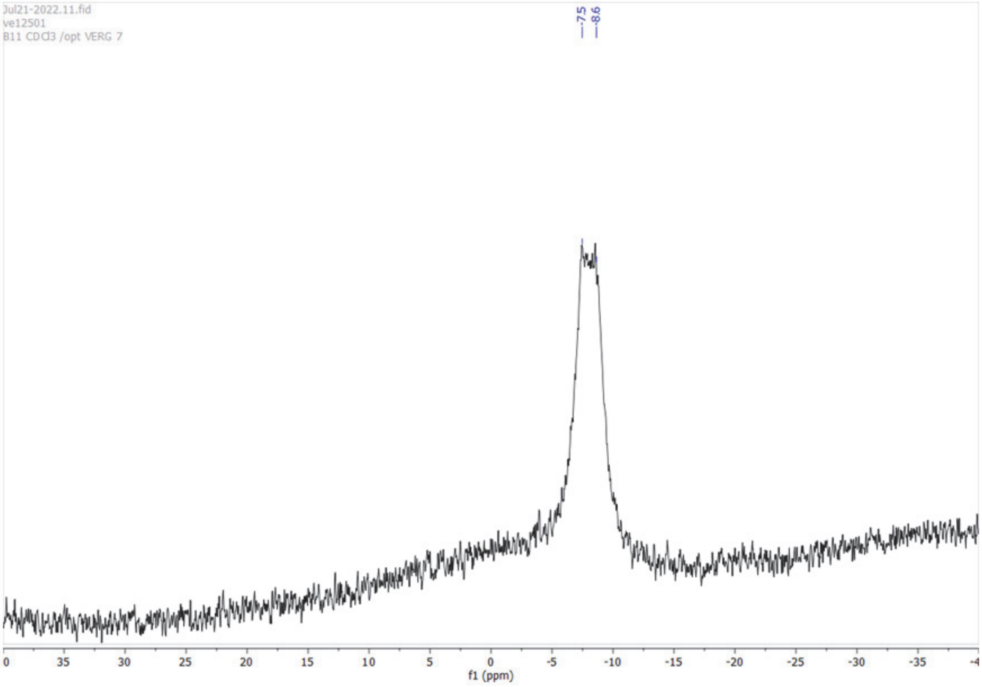

$^{13}C\{^1H\}$ ($CDCl_3$, 125 MHz) NMR of **$3h_{Tl}$**

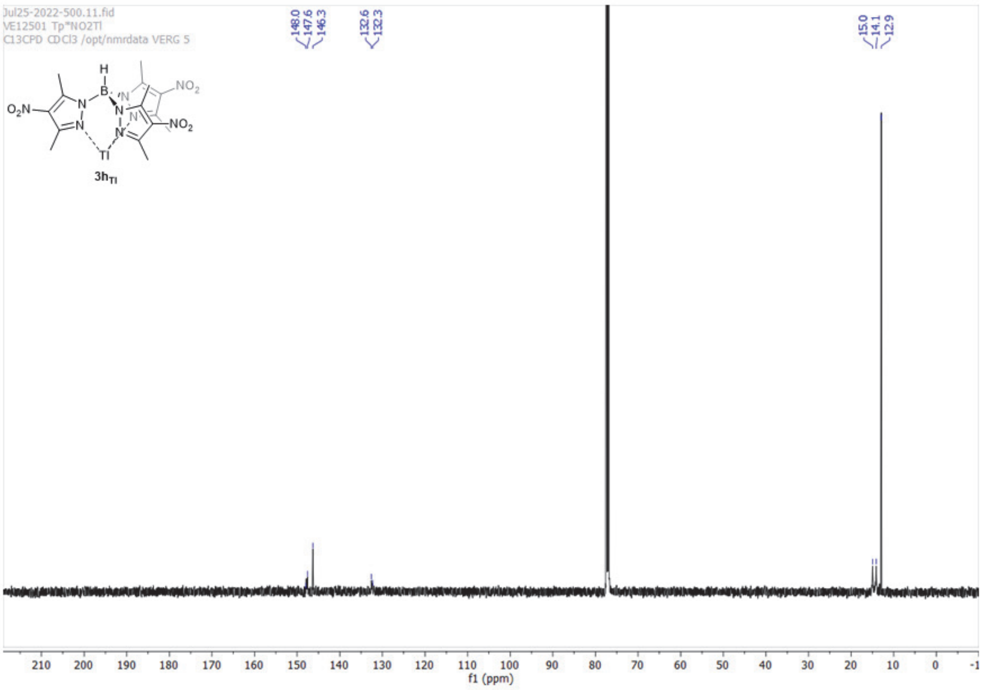

IR (ATR) spectrum of **$3h_{Tl}$**

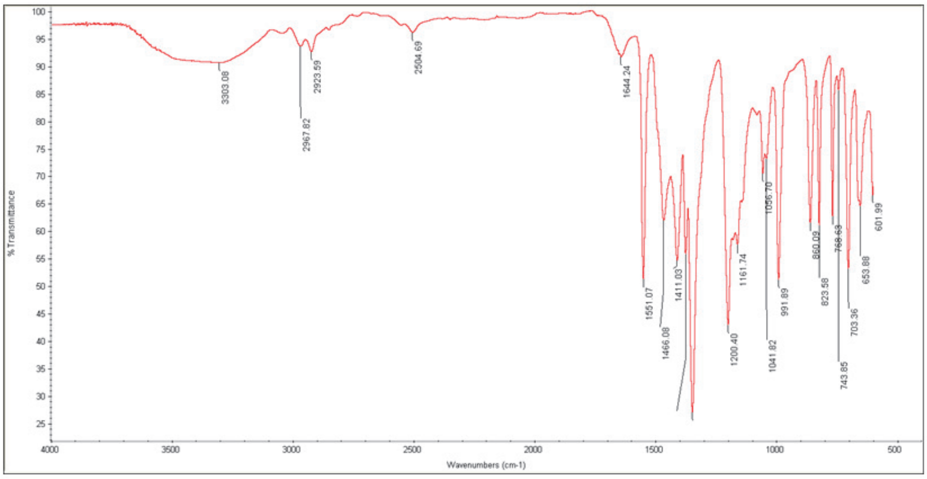

HRMS (+ESI) of **3h**$_{Na}$:

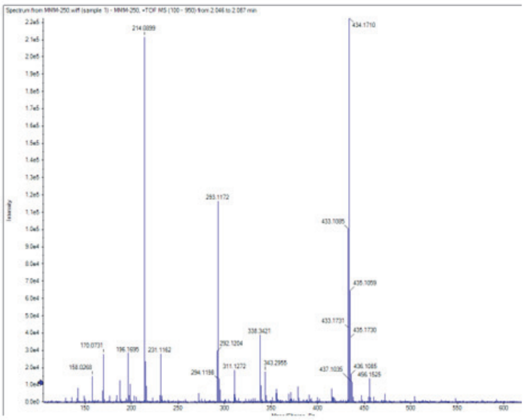

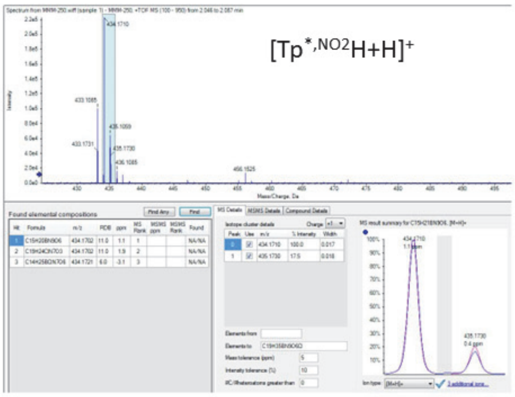

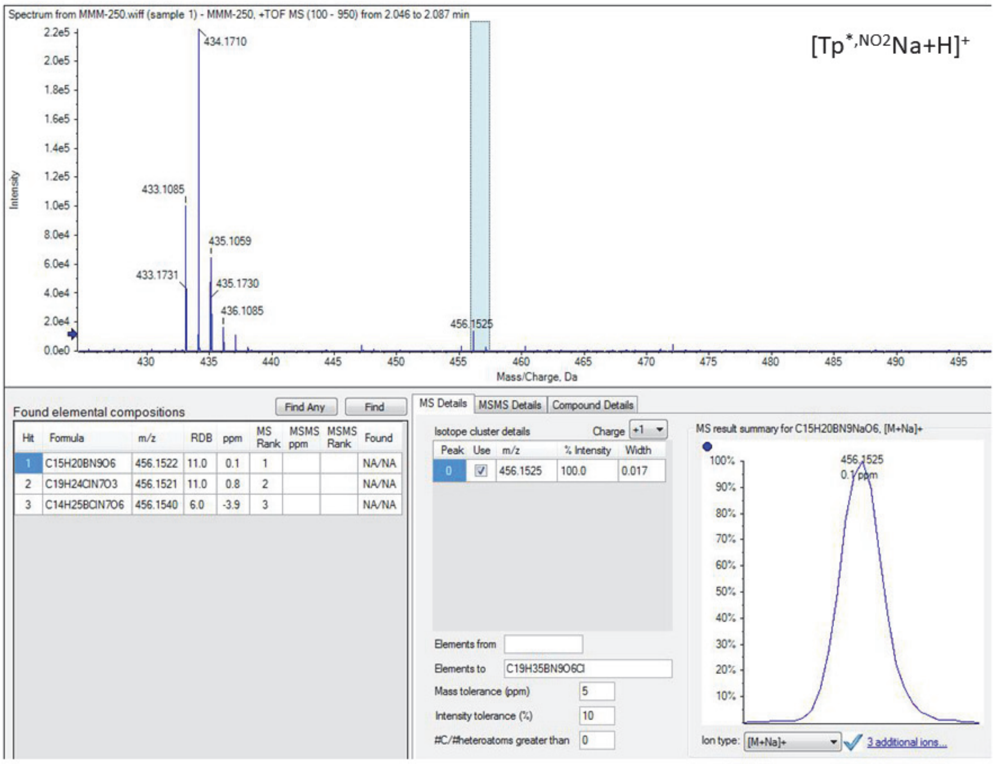

^{1}H ($CDCl_3$, 500 MHz) and $^{11}B\{^{1}H\}$ ($CDCl_3$, 96 MHz) NMR of crude reaction mixture for **$3i_{Tl}$**

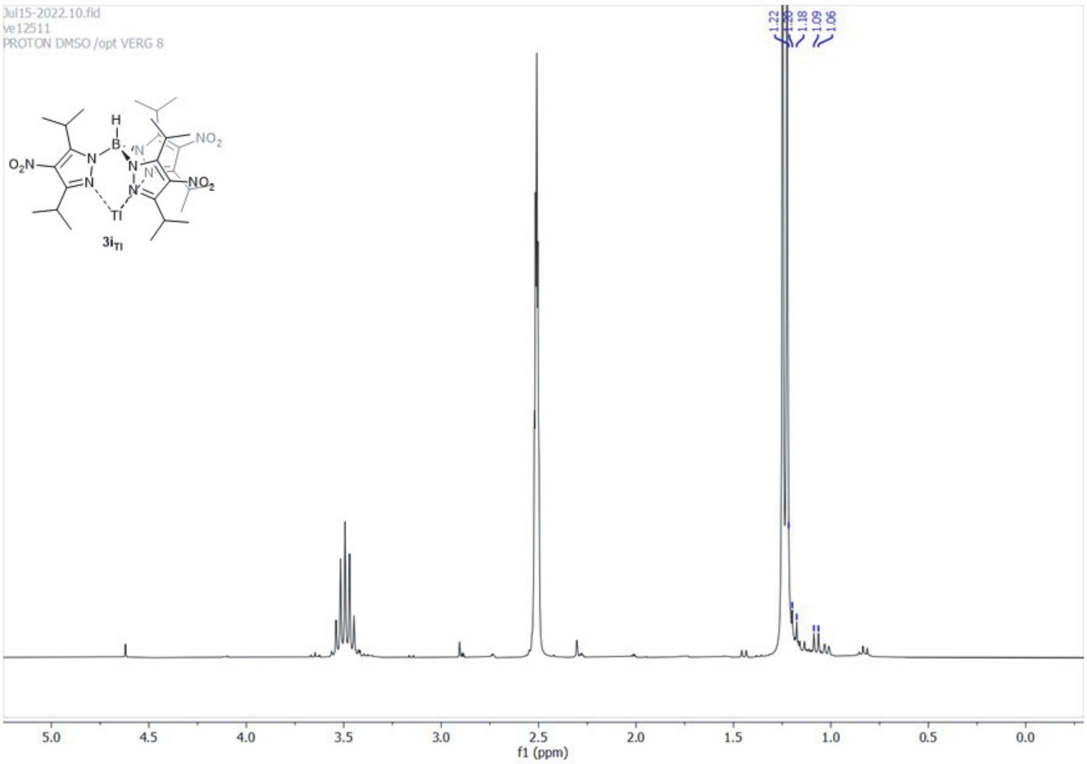

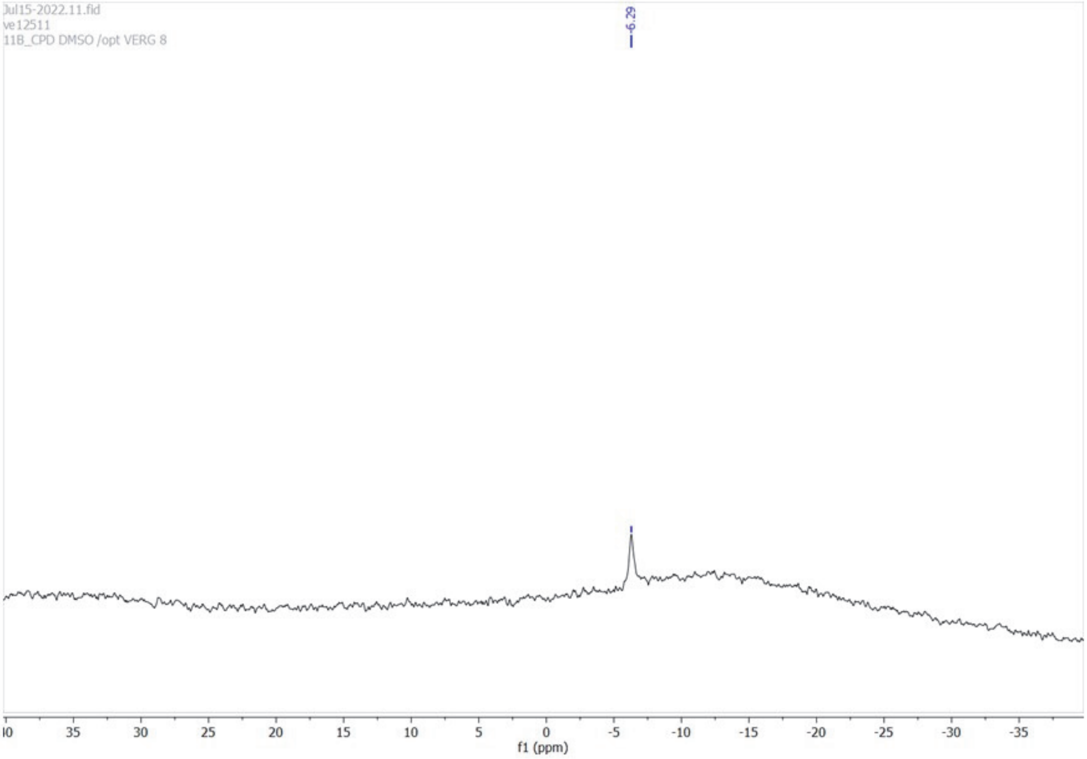

^{1}H ($CDCl_3$, 500 MHz) and $^{11}B\{^{1}H\}$ ($CDCl_3$, 160 MHz) NMR of crude reaction mixture for **3j$_{Tl}$**

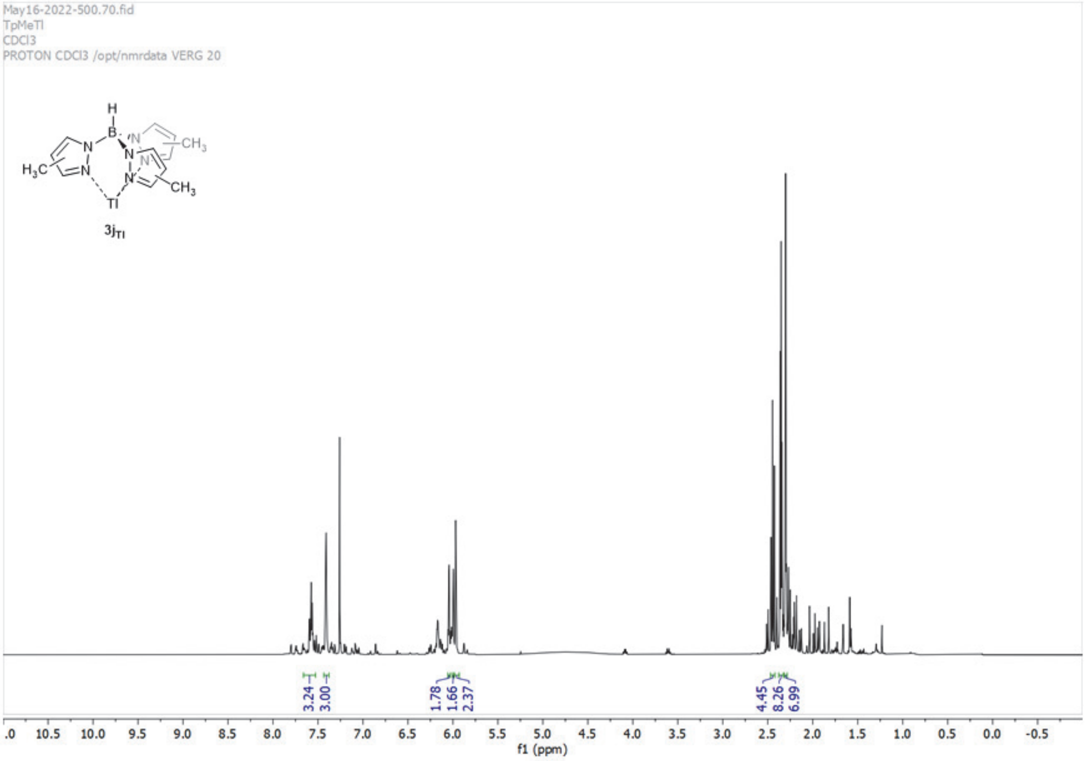

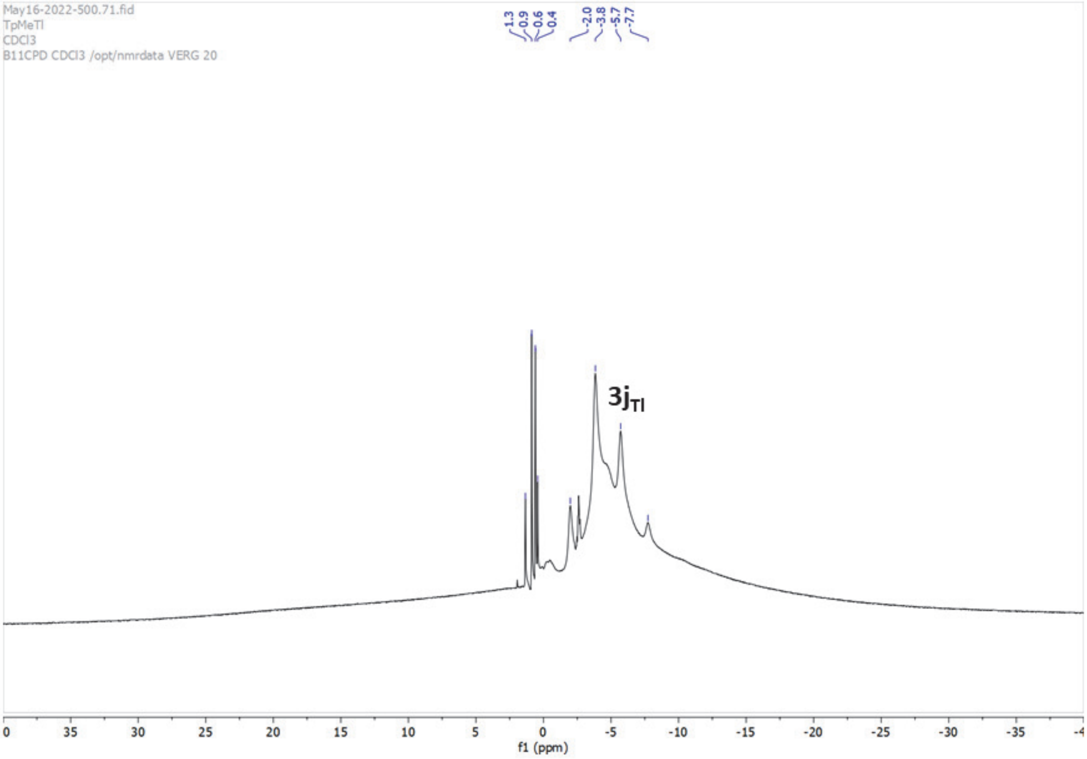

^{1}H ($CDCl_3$, 500 MHz) and ^{11}B ($CDCl_3$, 160 MHz) NMR of **$2a_{Tl}$·0,1 Hexane**

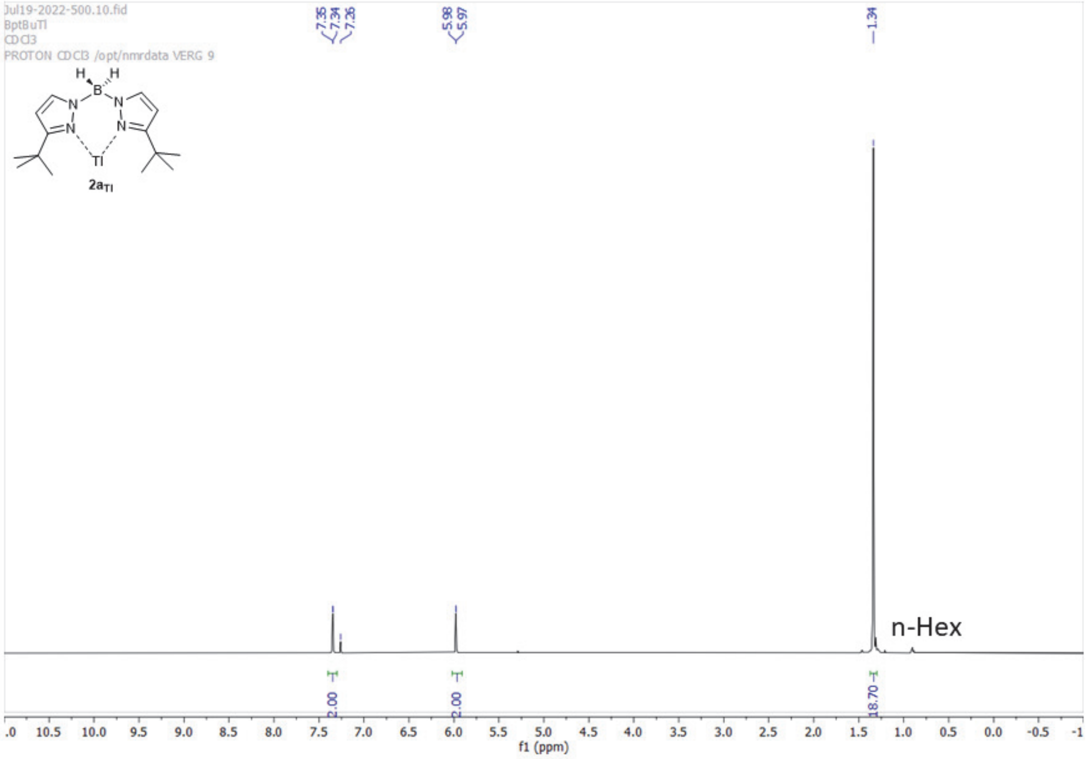

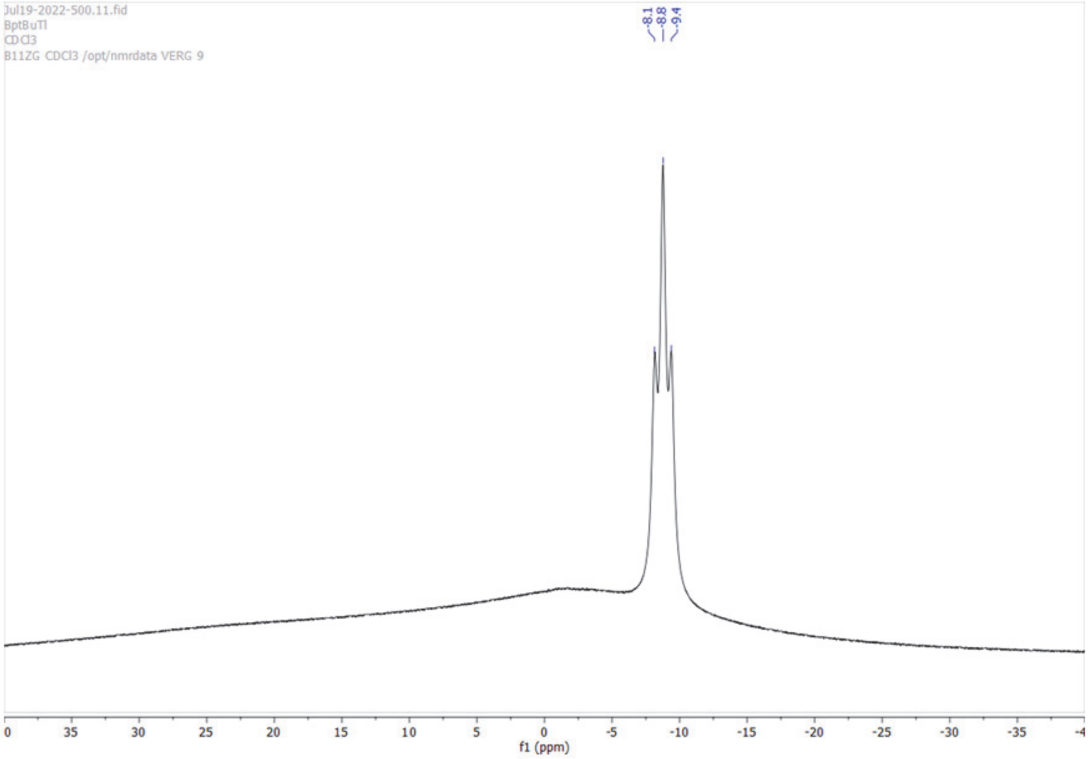

$^{13}C\{^{1}H\}$ ($CDCl_3$, 125 MHz) NMR of **$2a_{Tl}$ ·0,1 Hexane**

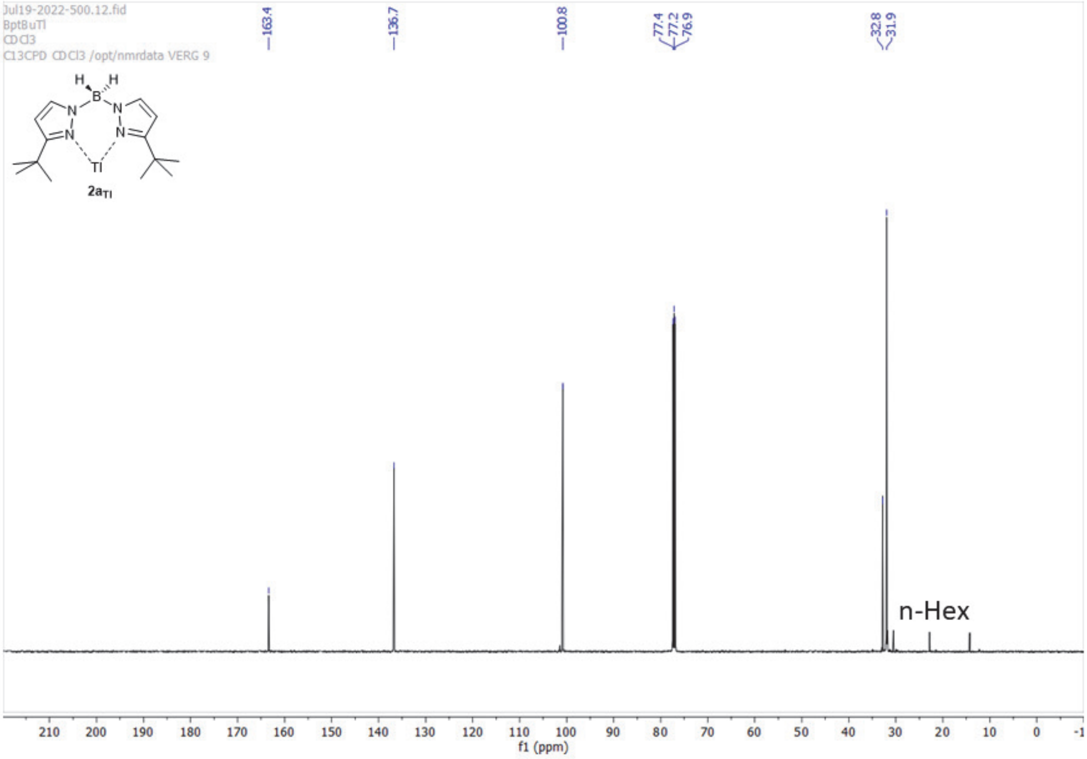

IR (ATR) spectrum of **$2a_{Tl}$**

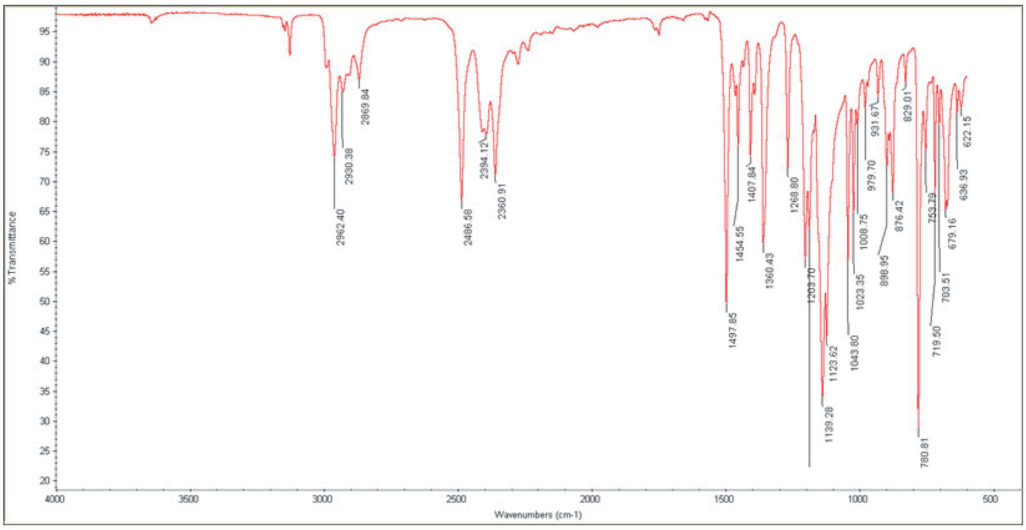

^{1}H ($CDCl_3$, 500 MHz) and ^{11}B ($CDCl_3$, 160 MHz) NMR of **$2b_{Tl}$**

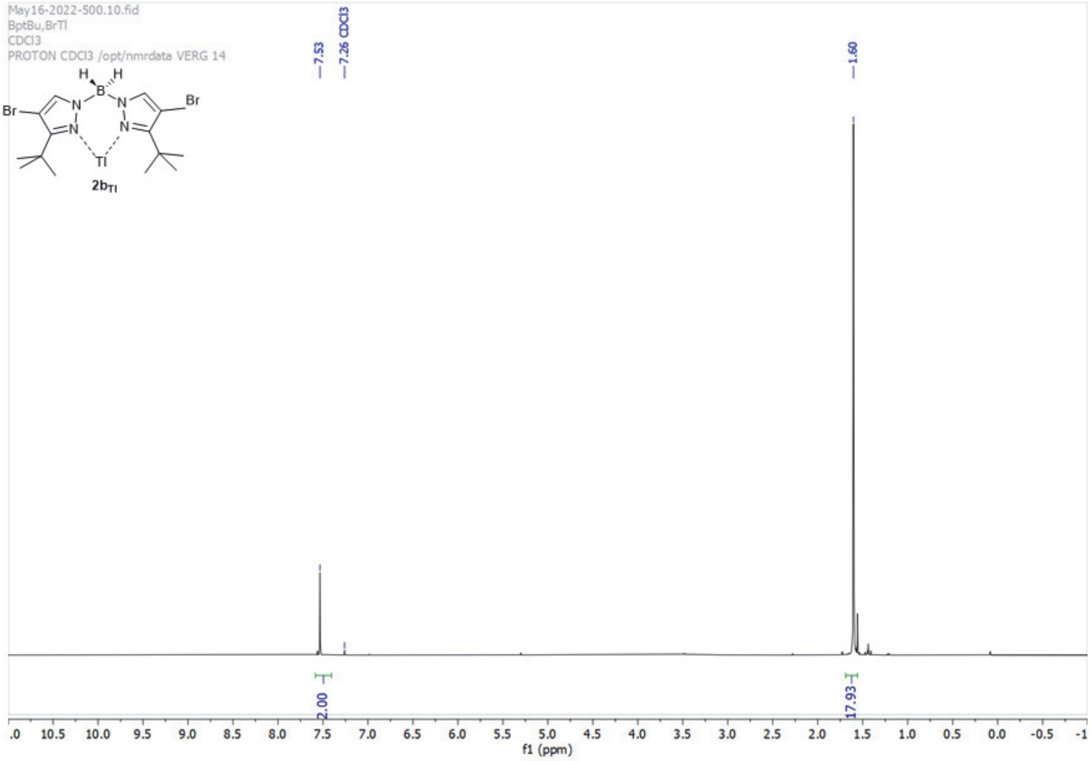

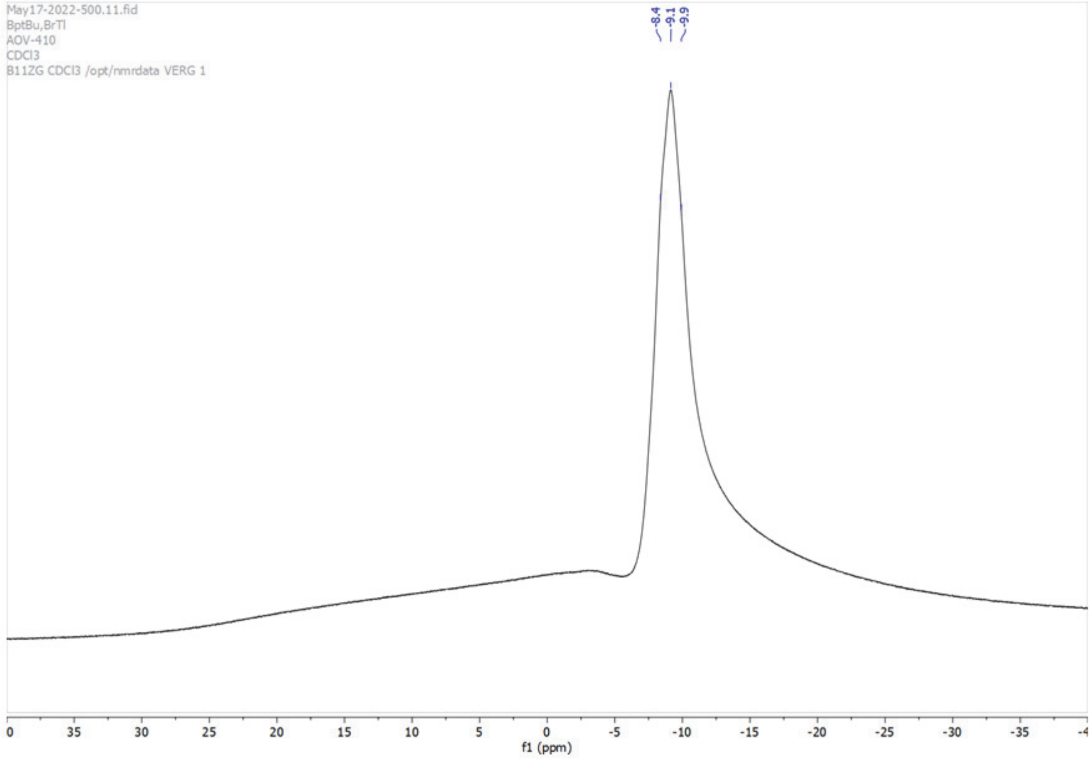

$^{13}C\{^{1}H\}$ ($CDCl_3$, 125 MHz) NMR of **$2b_{Tl}$**

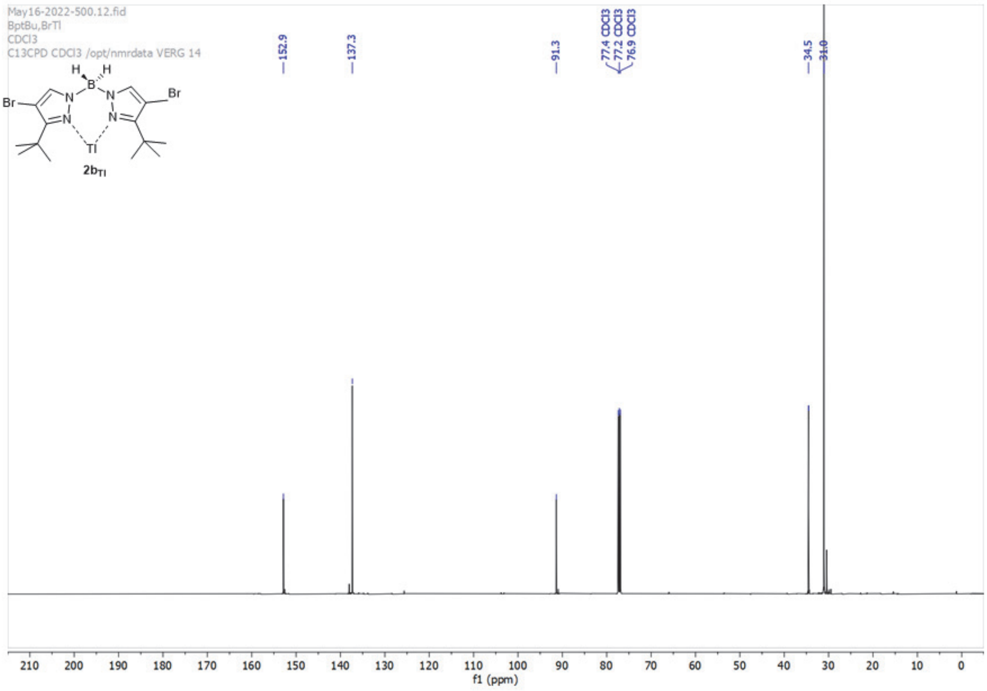

IR (ATR) spectrum of **$2b_{Tl}$**

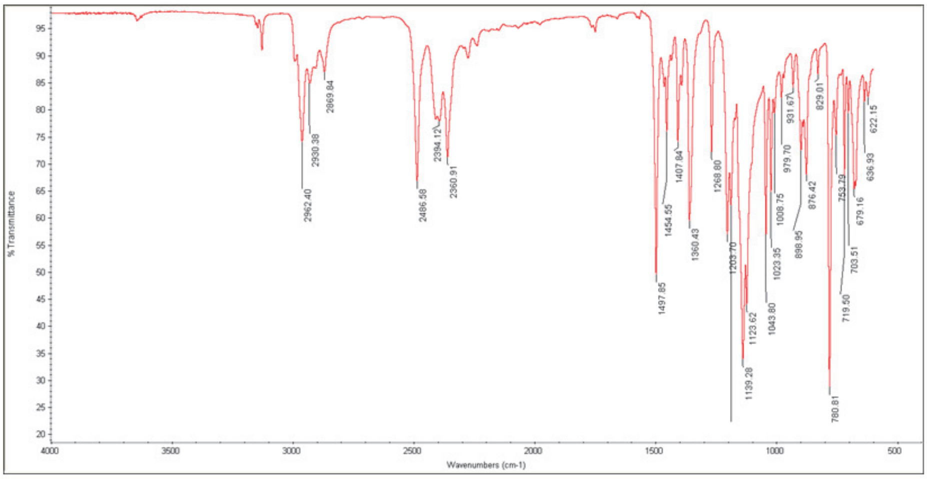

^{1}H (DMSO-d_6, 500 MHz) NMR of **$2b_{Tl}$·THF**

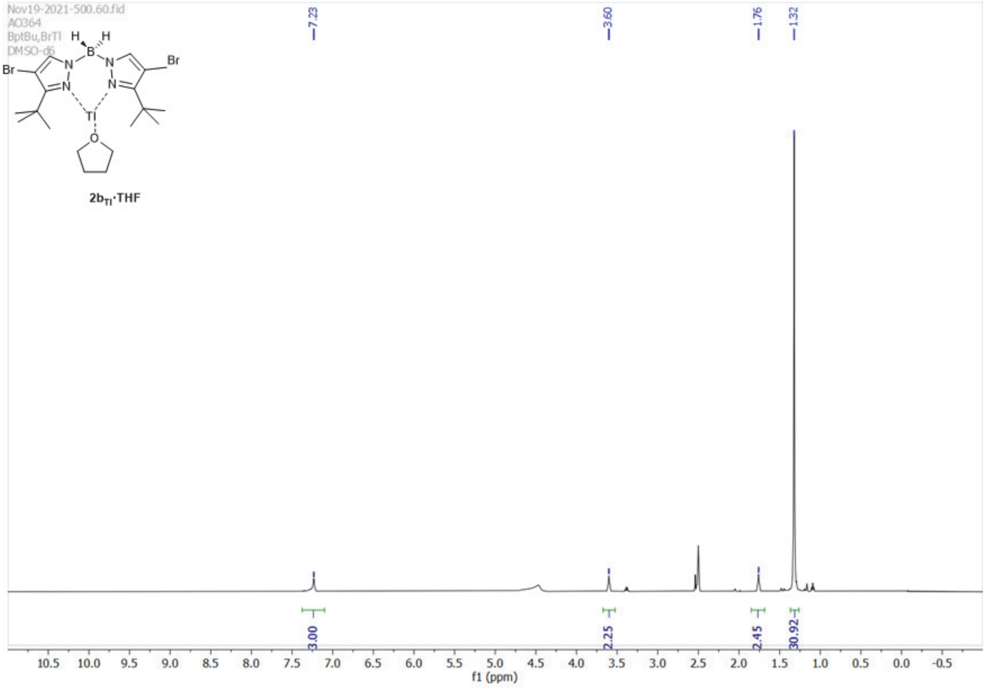

HRMS (+ESI) of **$2b_{Tl}$**:

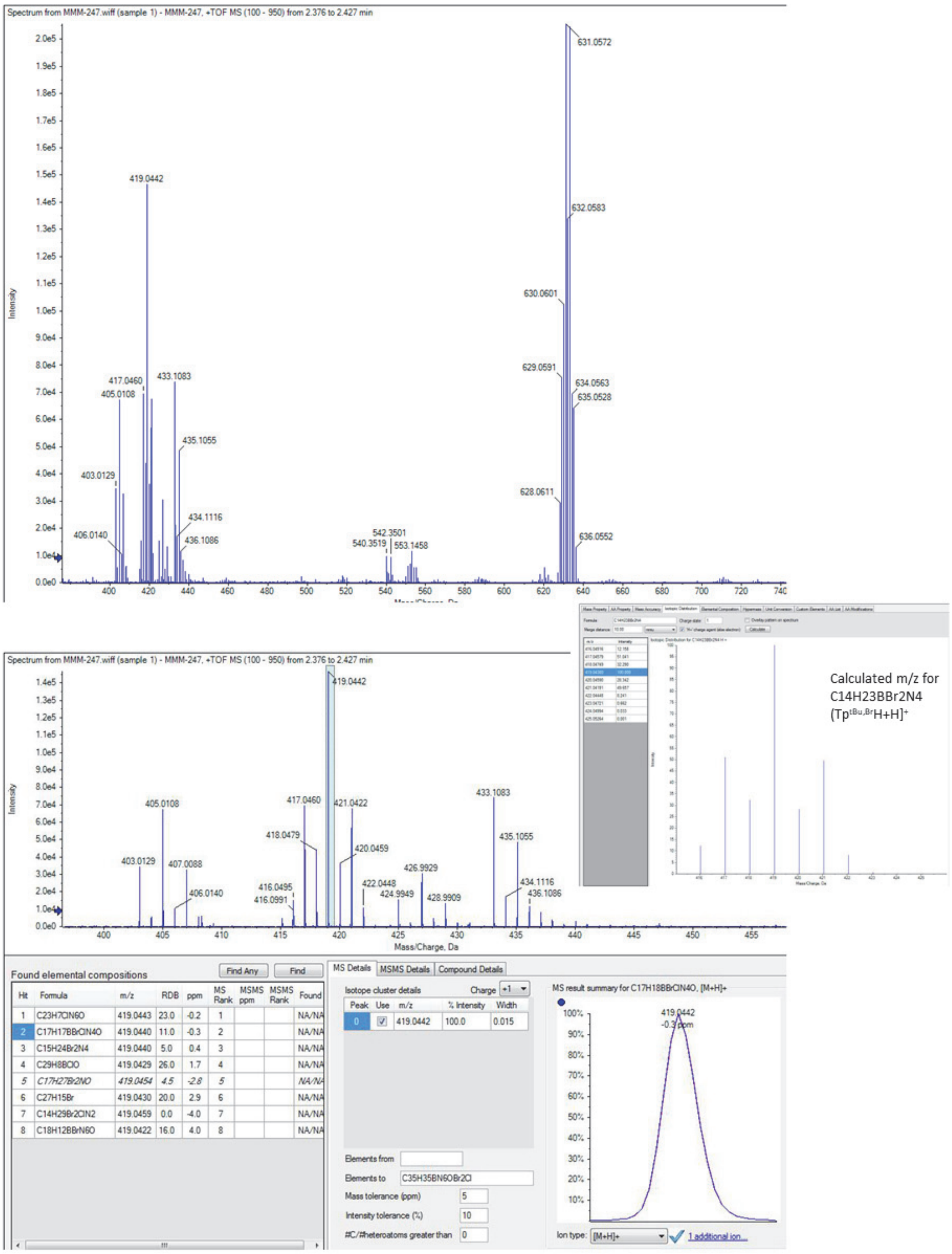

^{1}H (CD_2Cl_2, 500 MHz) and ^{11}B (CD_2Cl_2, 160 MHz) NMR of **$2c_{Tl}$·0,28DCM**

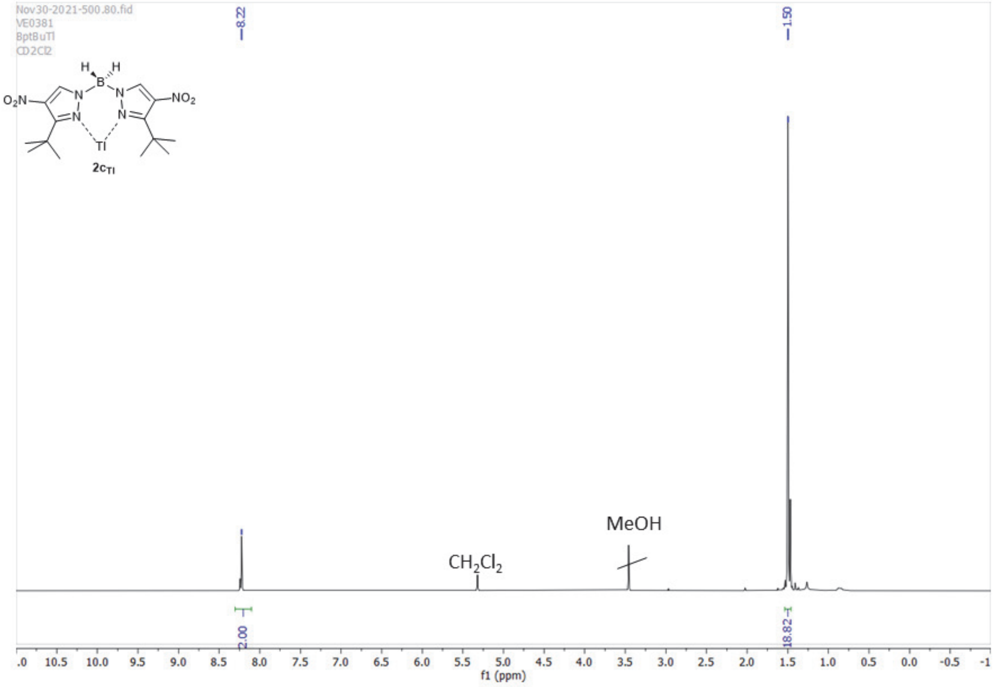

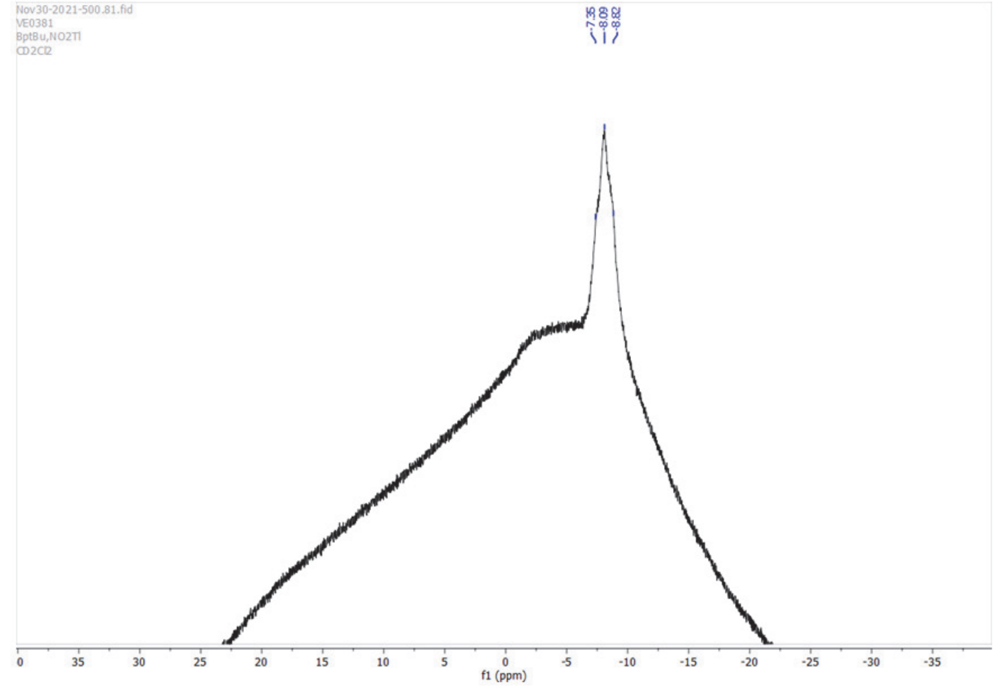

$^{13}C\{^{1}H\}$ ($CDCl_3$, 125 MHz) NMR of **2c$_{Tl}$**

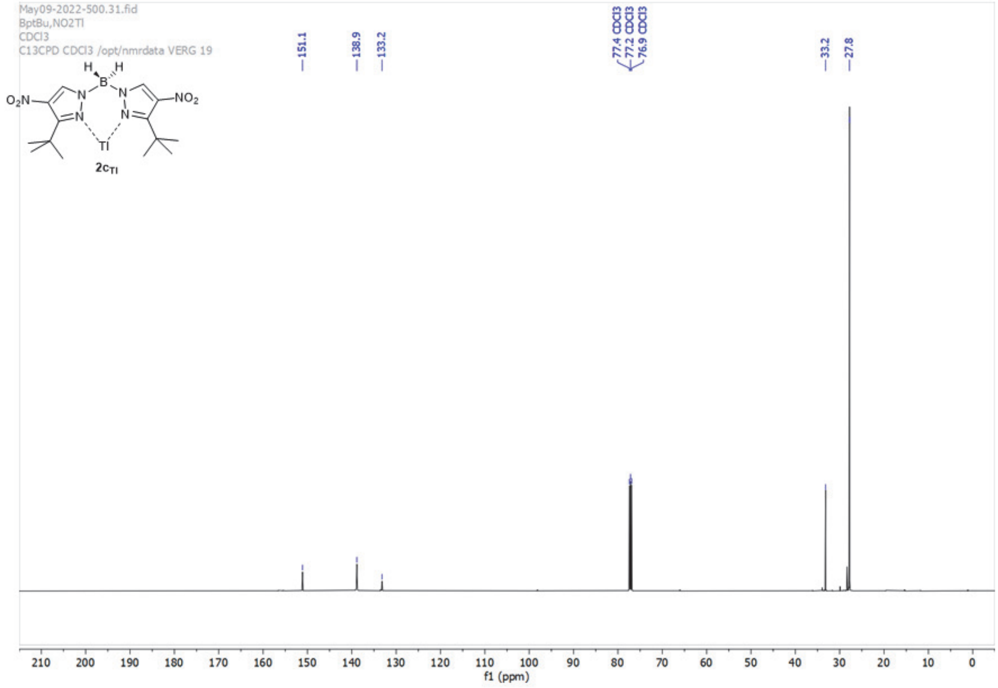

IR (ATR) spectrum of **2c$_{Tl}$**

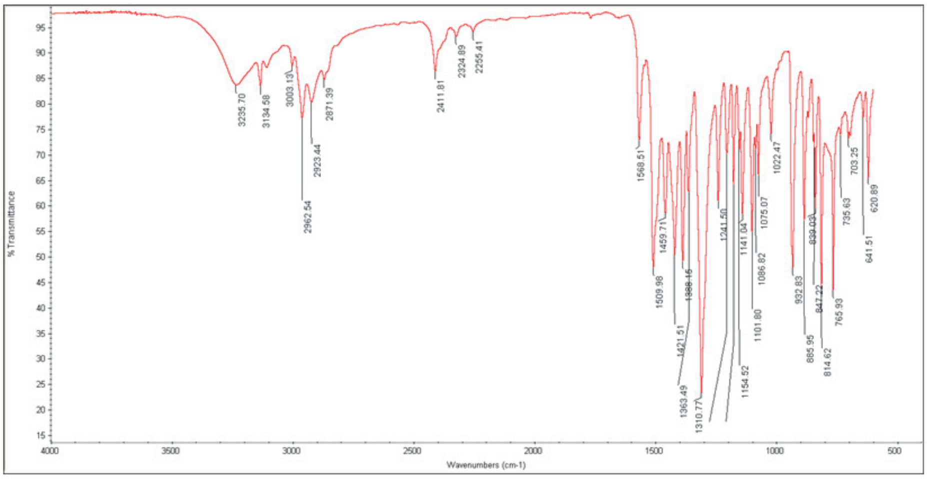

HRMS (+ESI) of **$2c_{Tl}$**:

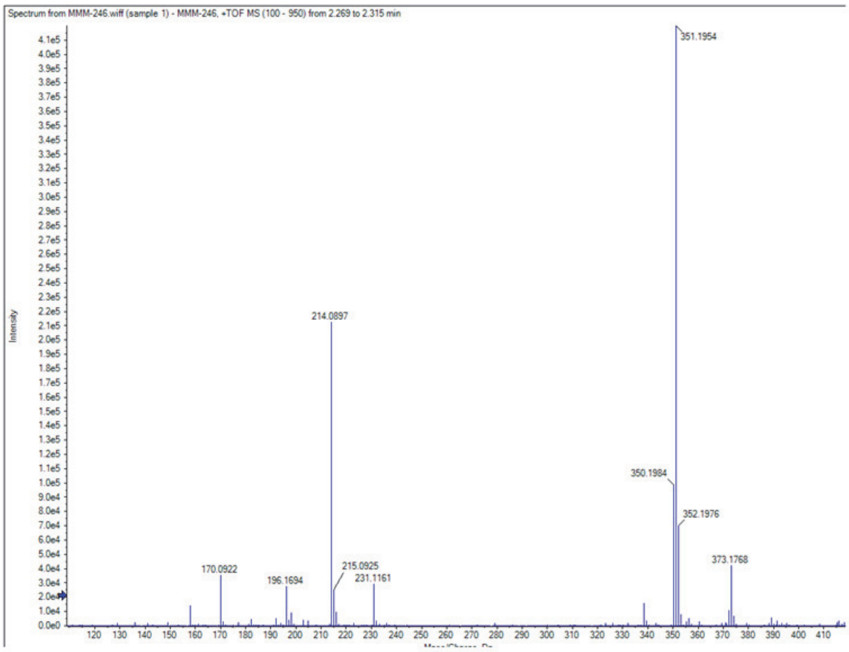

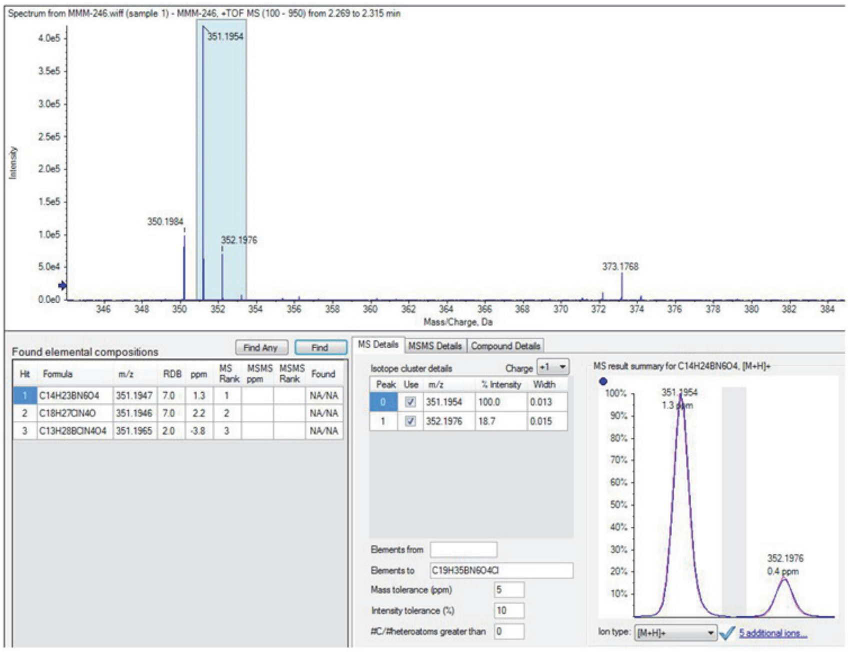

^1H ($CDCl_3$, 500 MHz) and ^{11}B ($CDCl_3$, 160 MHz) NMR of **$2f_{Tl}$**

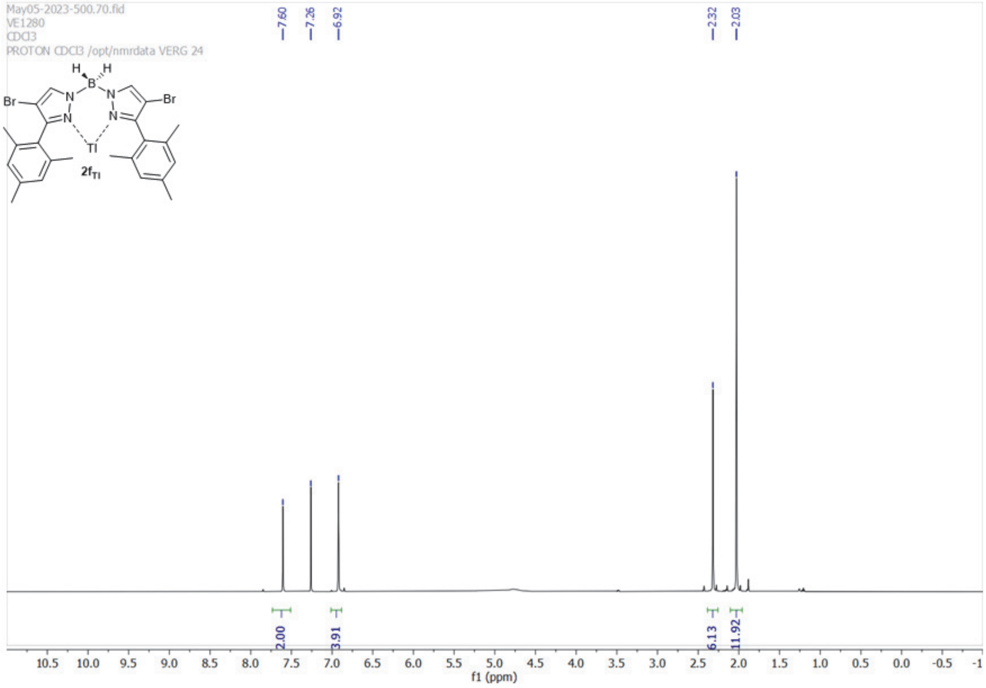

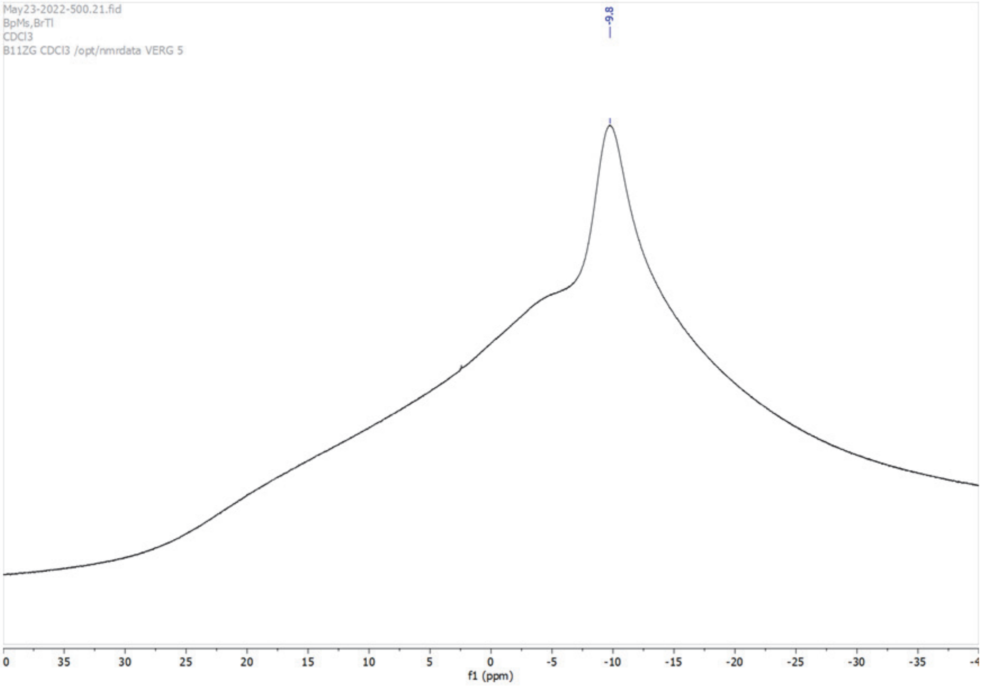

$^{13}C\{^1H\}$ ($CDCl_3$, 125 MHz) NMR of **2f**$_{Tl}$ in $CDCl_3$

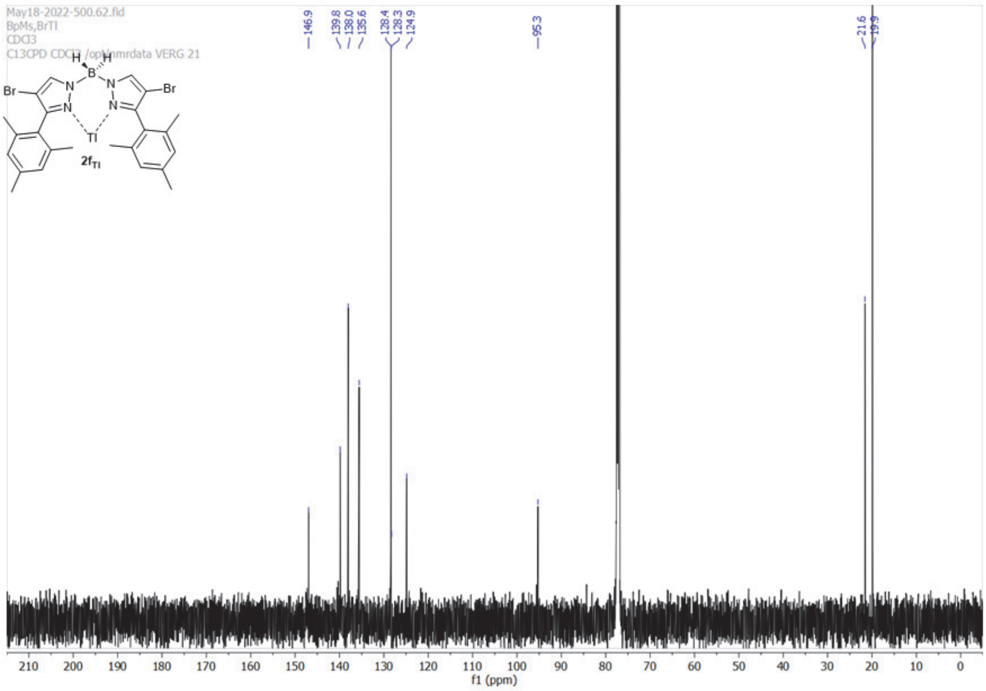

IR (ATR) spectrum of **2f**$_{Tl}$

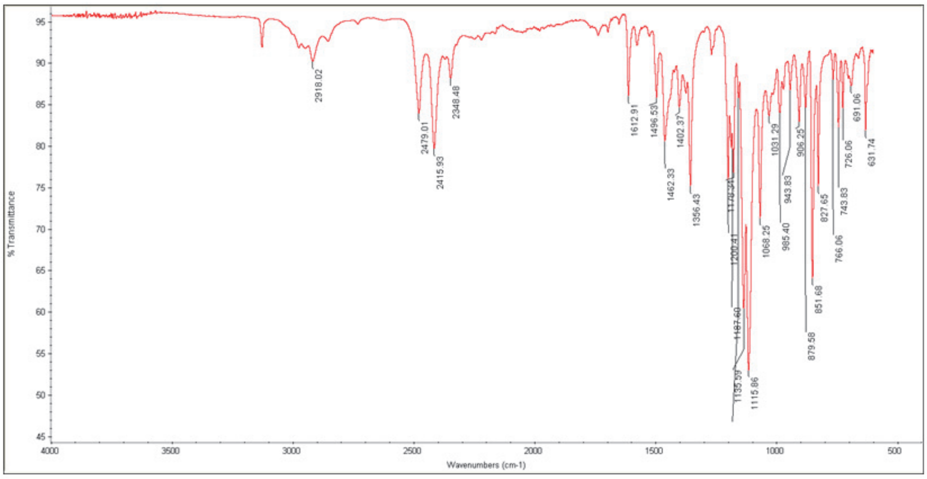

^{1}H ($CDCl_3$, 500 MHz) and ^{11}B ($CDCl_3$, 160 MHz) NMR of **1,7-bis(mesityl)pyrazabole**

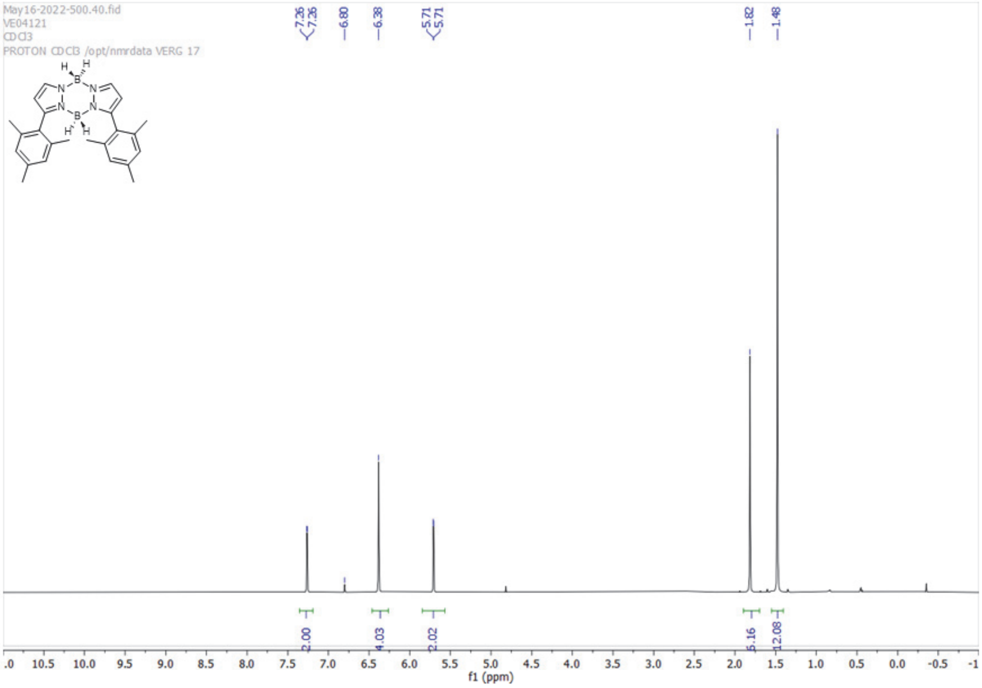

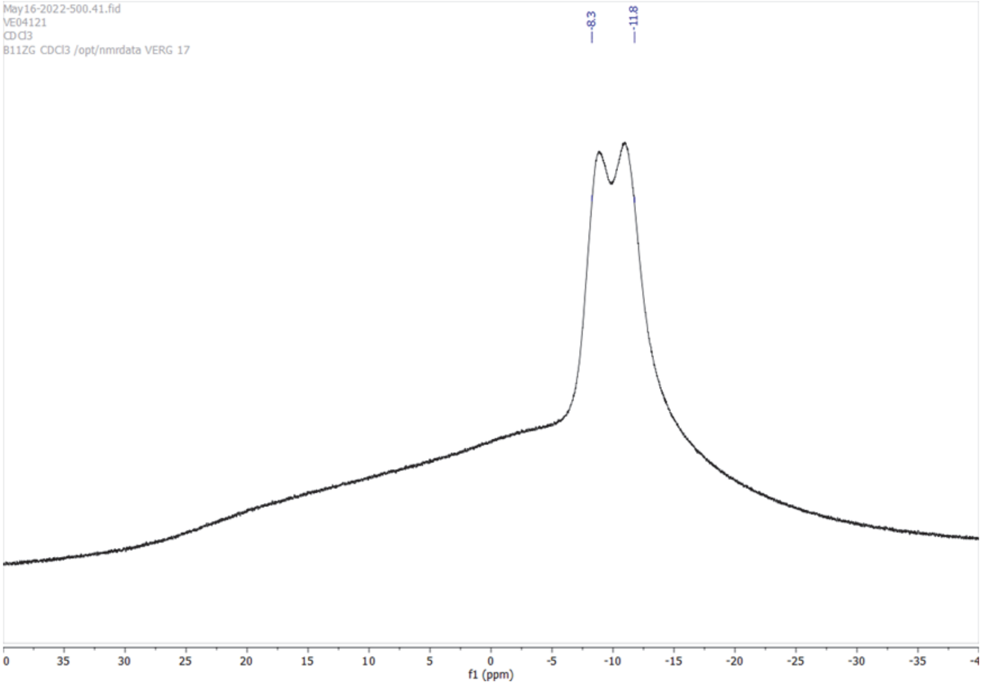

^1H ($CDCl_3$, 500 MHz) and ^{11}B ($CDCl_3$, 160 MHz) NMR of **1,5-bis(mesityl)pyrazabole**

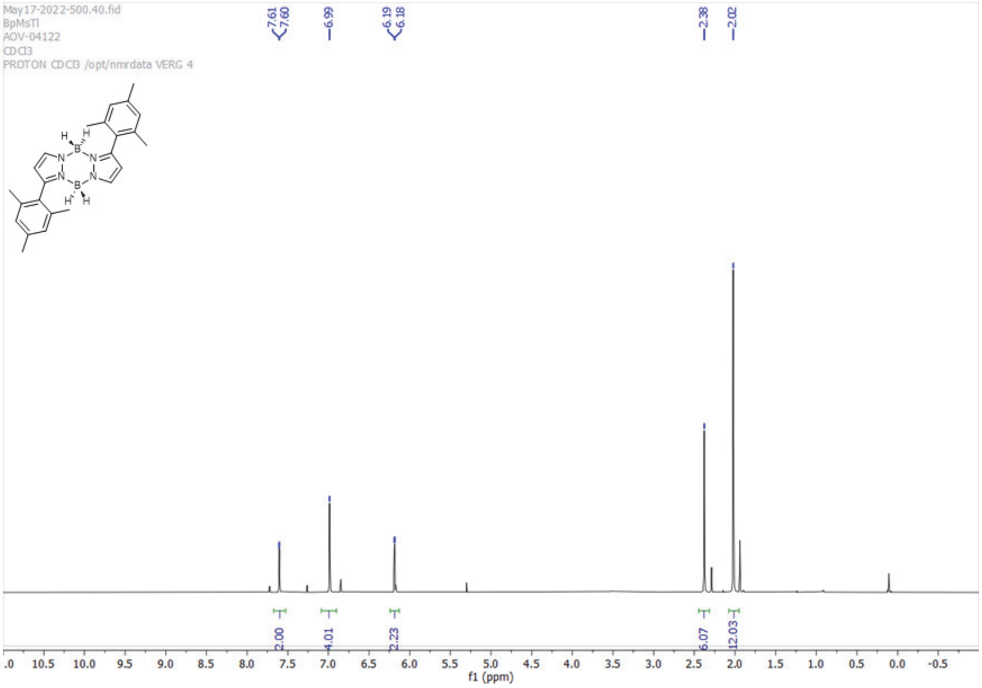

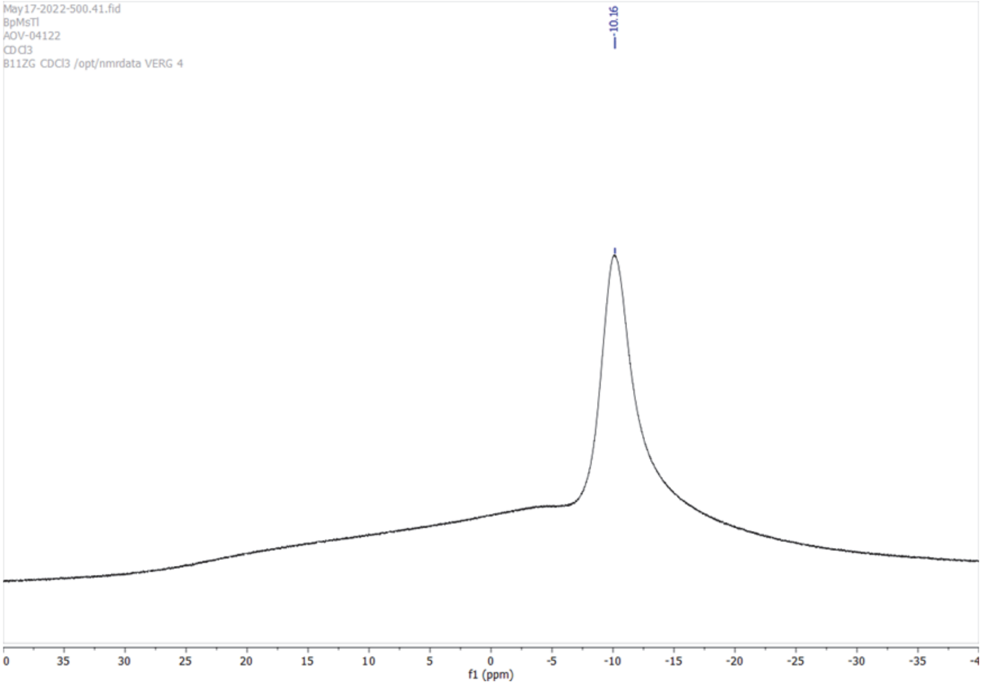

^{1}H ($CDCl_3$, 500 MHz) and ^{11}B ($CDCl_3$, 160 MHz) NMR of **2,6-dibromo-1,5-bis(mesityl)pyrazabole**

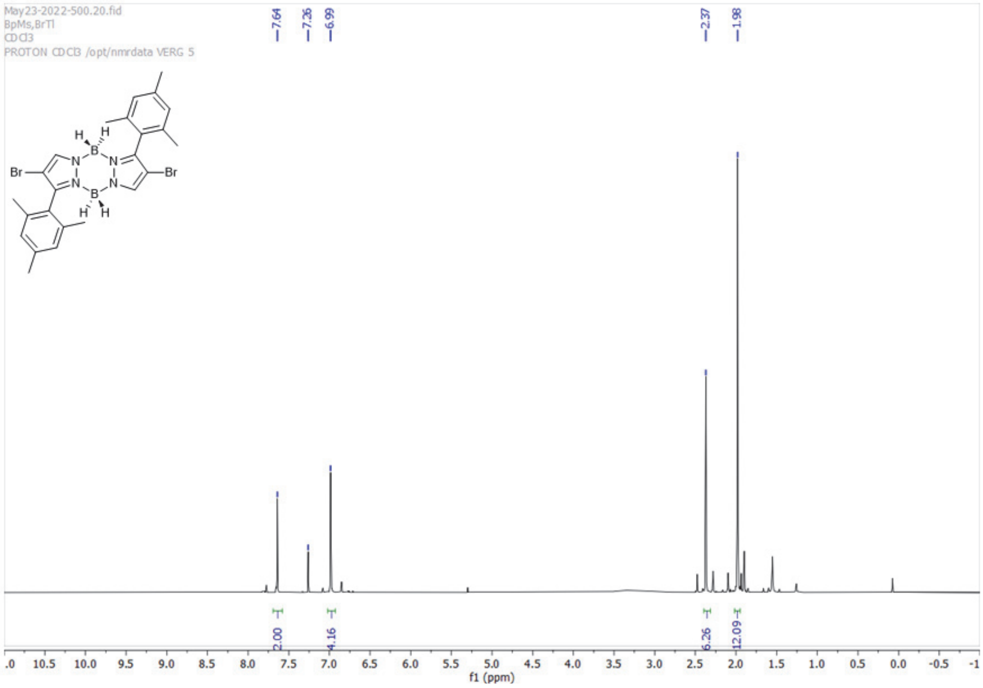

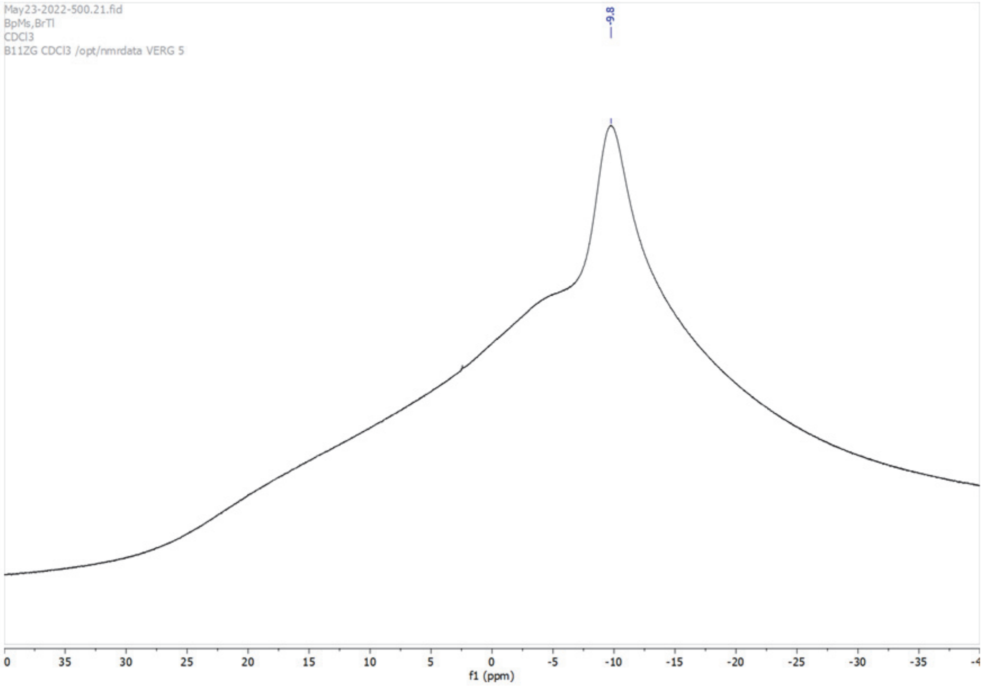

$^{13}C\{^{1}H\}$ ($CDCl_3$, 125 MHz) NMR of **2,6-dibromo-1,5-bis(mesityl)pyrazabole**

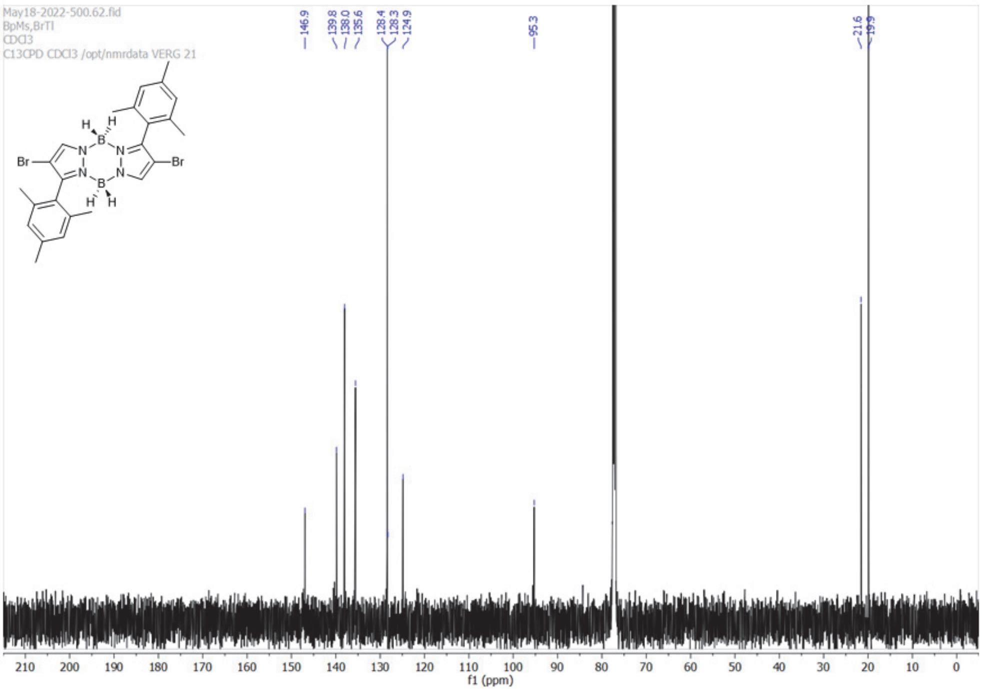

IR (ATR) spectrum of **2,6-dibromo-1,5-bis(mesityl)pyrazabole**

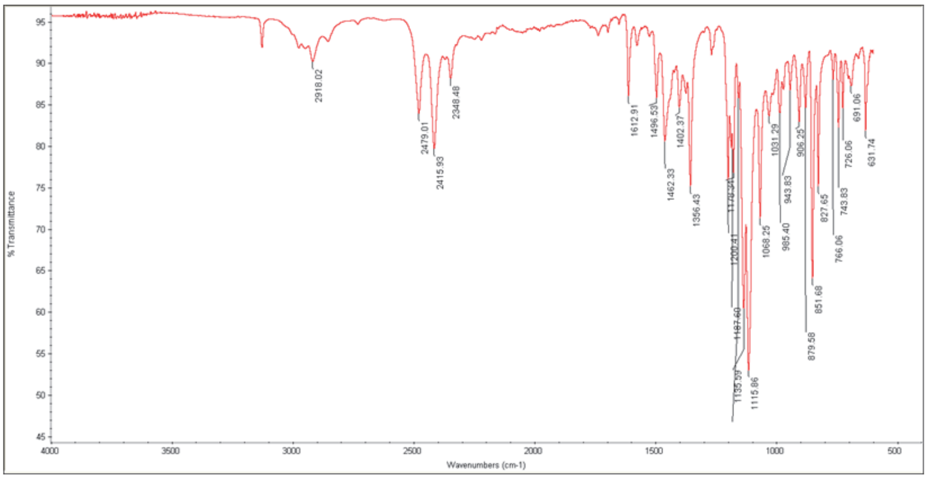

^1H (DMSO-d_6, 500 MHz) and ^{11}B (DMSO-d_6, 160 MHz) NMR of **$4a_{Na}$·1/3THF**

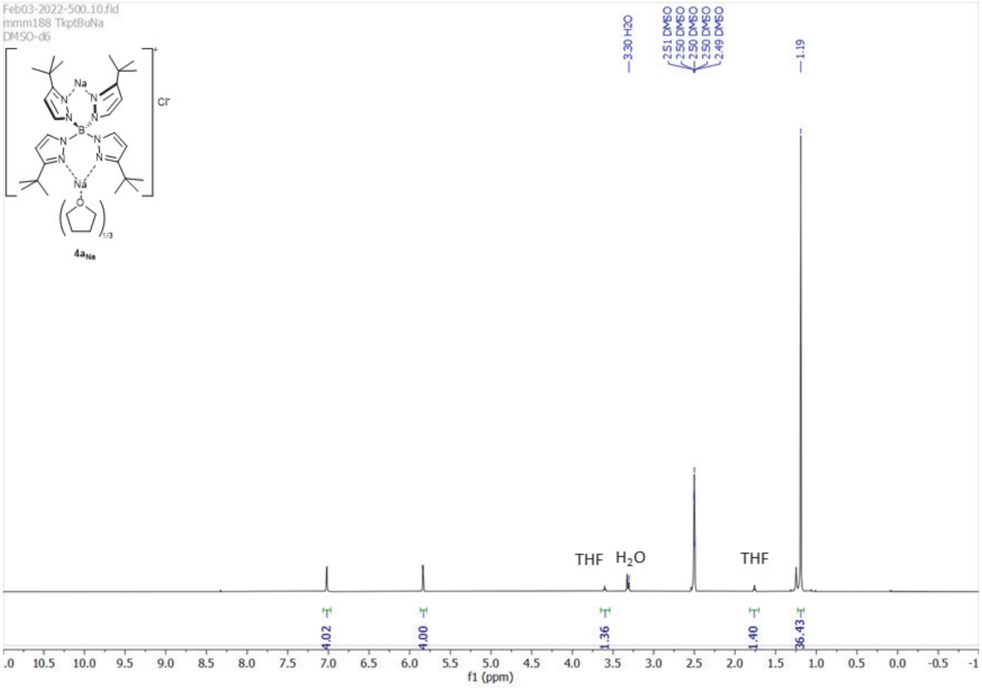

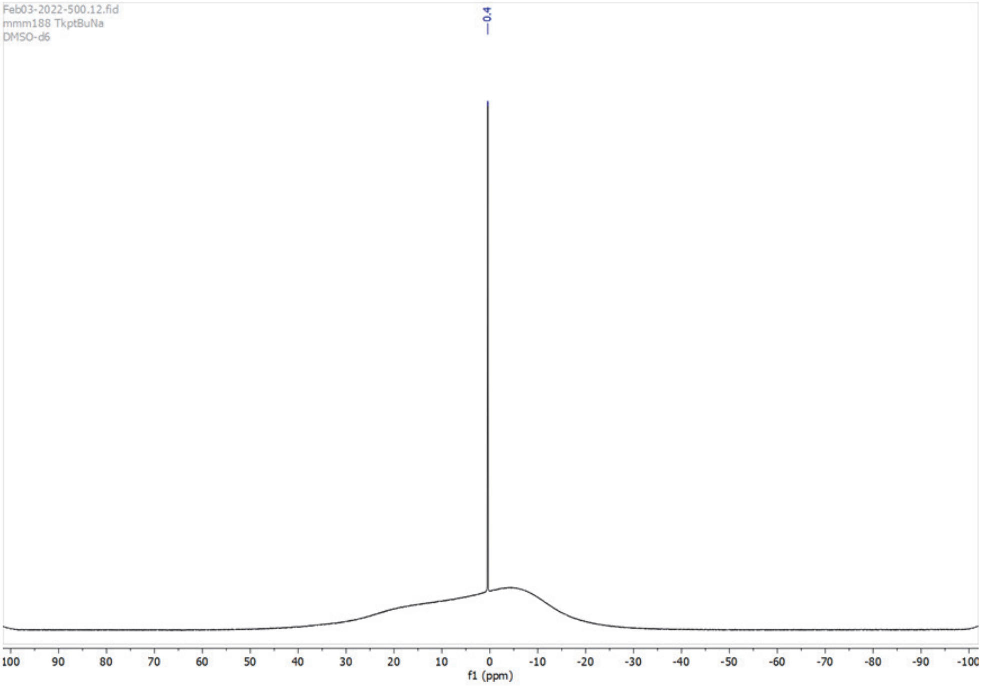

$^{13}C\{^1H\}$ (DMSO-d_6, 125 MHz) NMR of **4a$_{Na}$·1/3THF**

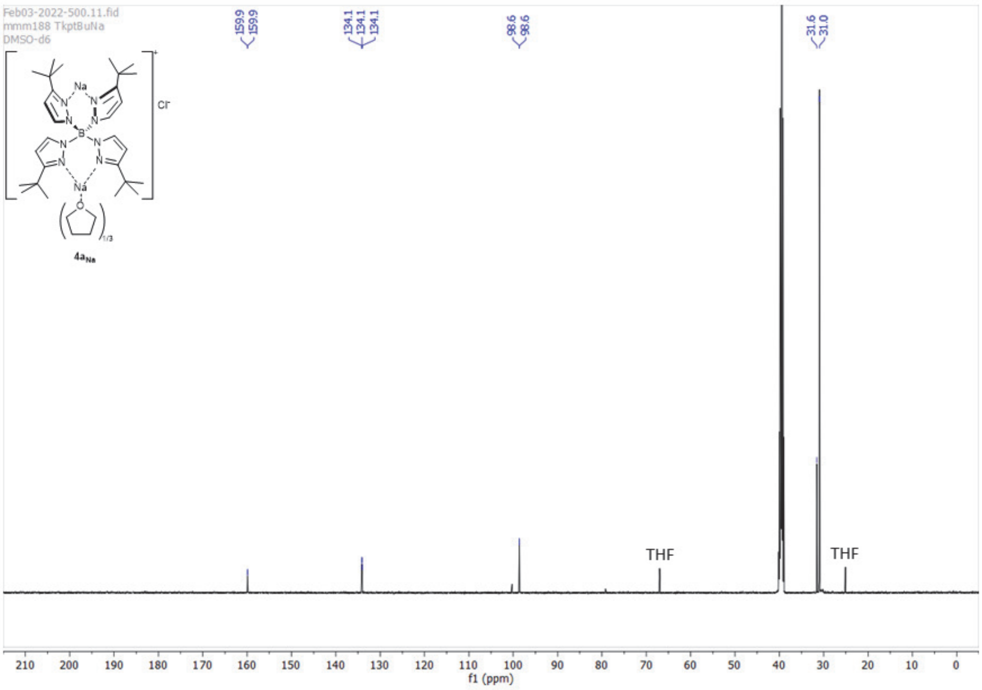

IR (ATR) spectrum of **4a$_{Na}$**

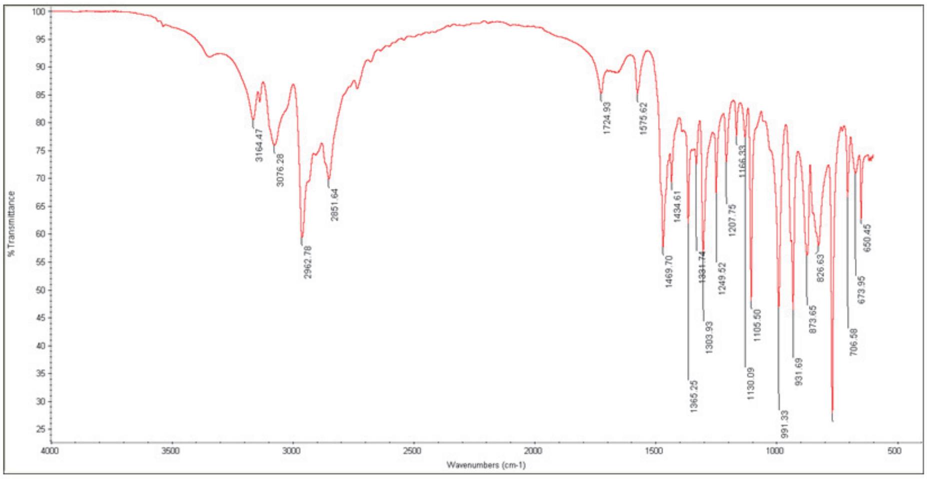

^{1}H (DMSO-d_6, 500 MHz) and ^{11}B (DMSO-d_6, 160 MHz) NMR of **$4b_{Na}$**

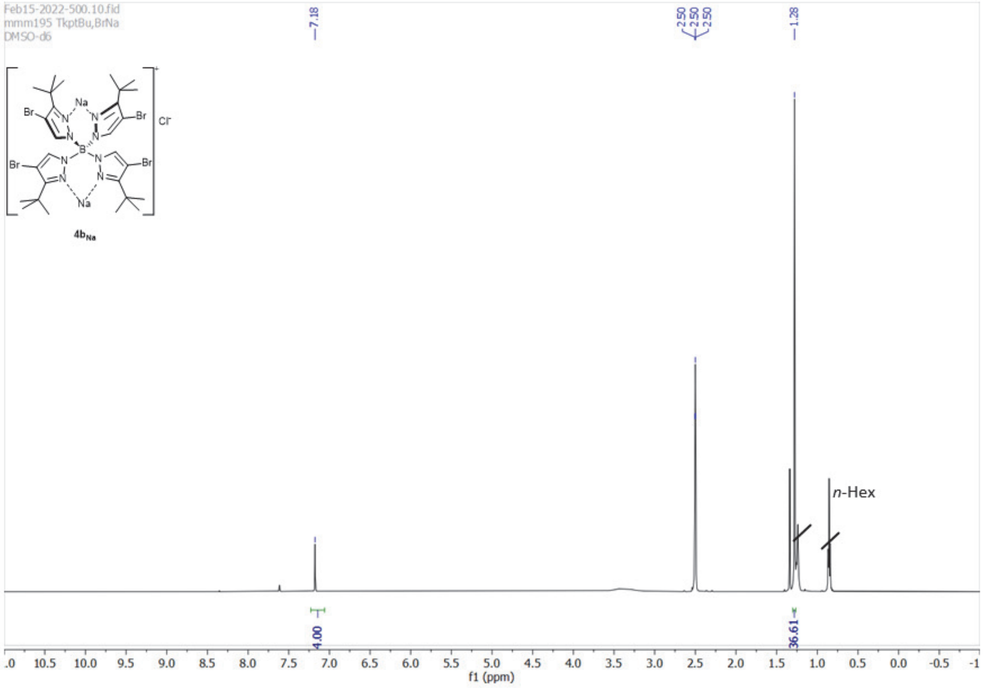

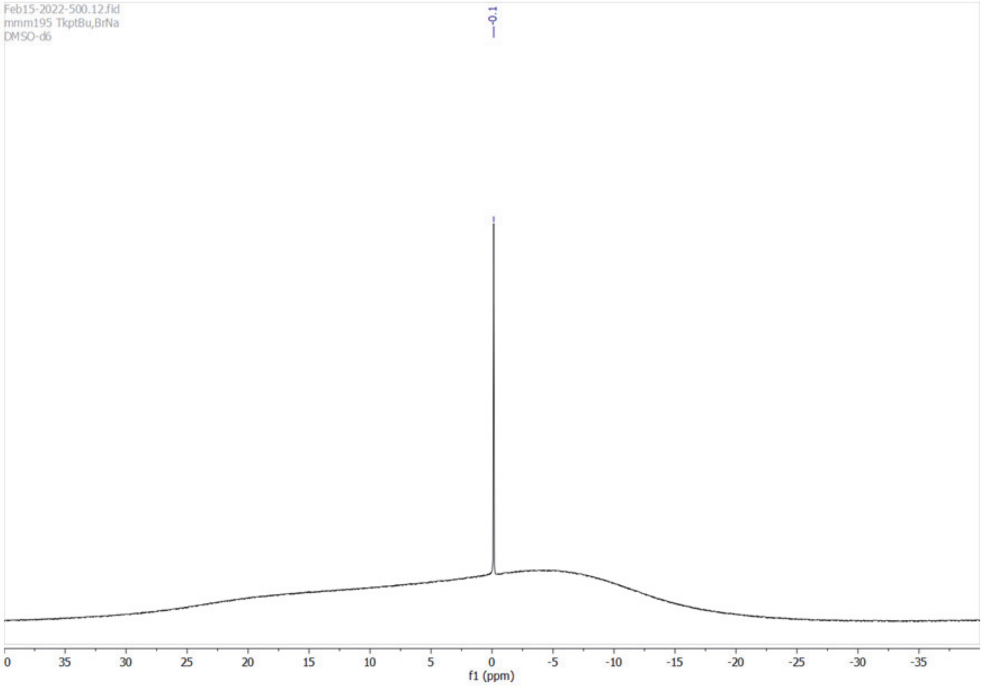

$^{13}C\{^1H\}$ (DMSO-d_6, 125 MHz) NMR of $\mathbf{4b_{Na}}$

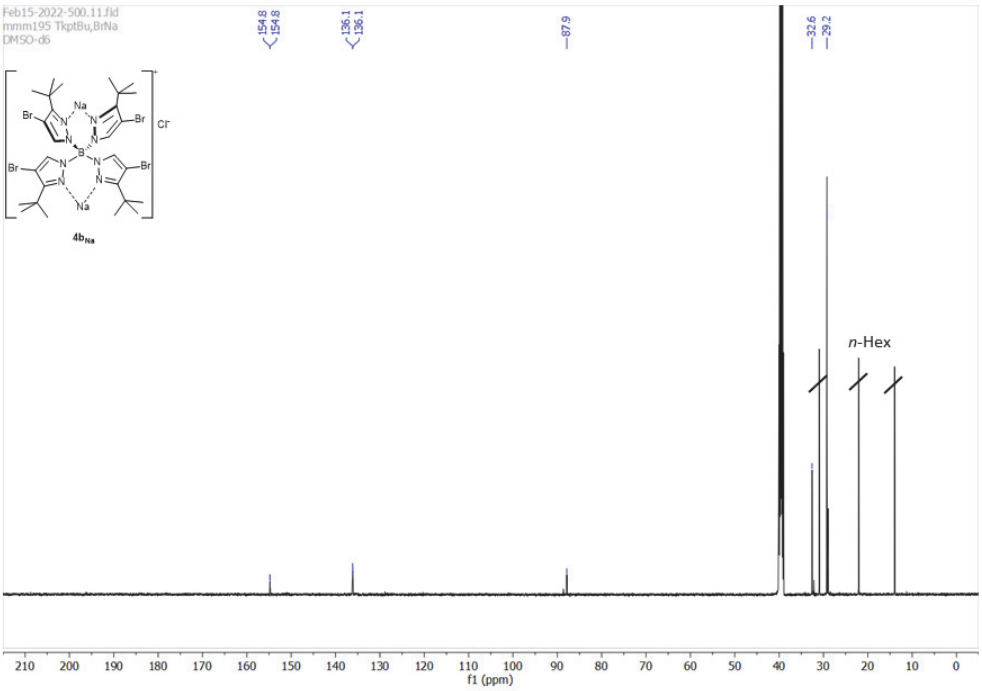

IR (ATR) spectrum of $\mathbf{4b_{Na}}$

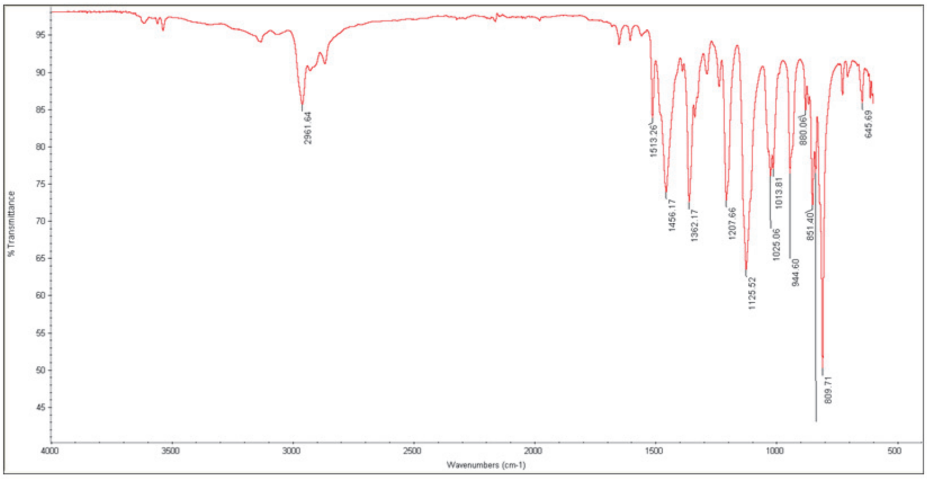

^{1}H (C_6D_6, 500 MHz) and ^{11}B (DMSO-d_6, 160 MHz) NMR of **$4c_{Na}$·1/3THF**

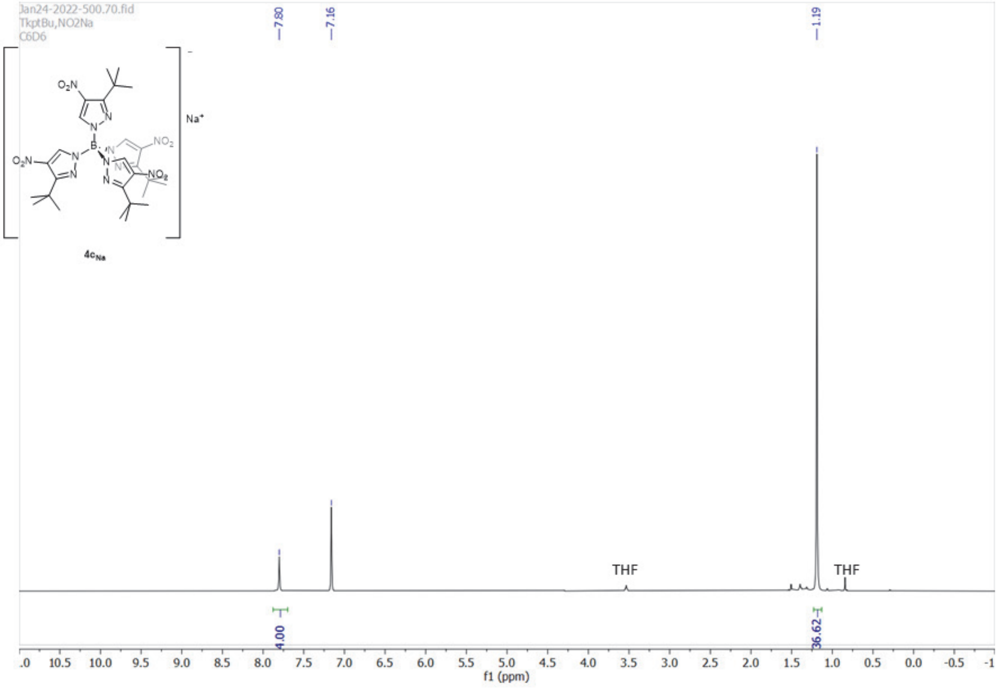

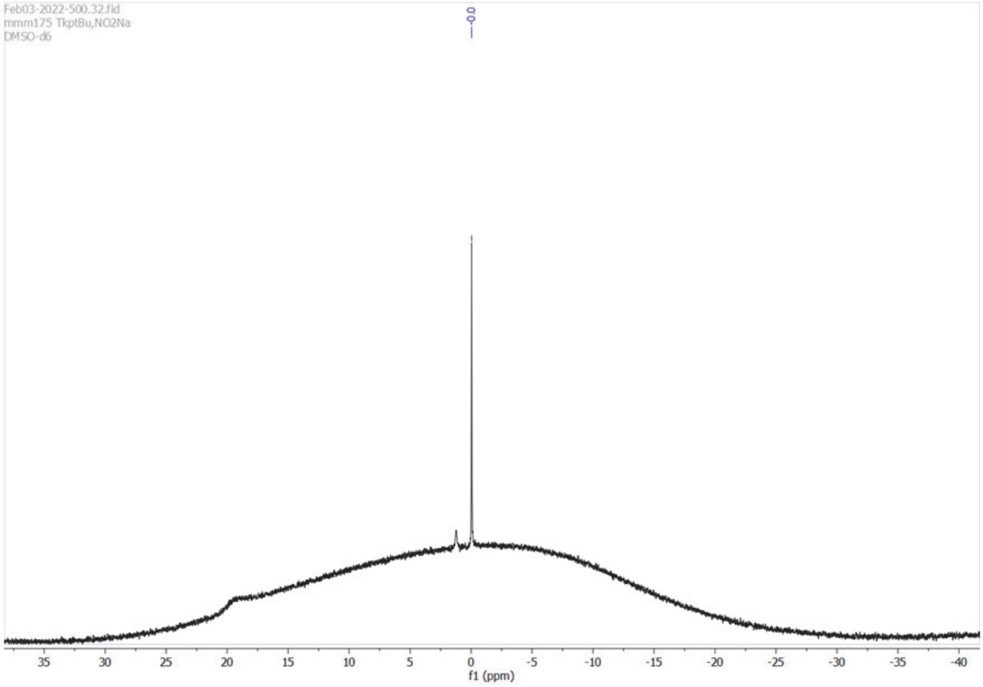

$^{13}C\{^1H\}$ (C_6D_6, 125 MHz) NMR of **$4c_{Na}$·1/3THF**

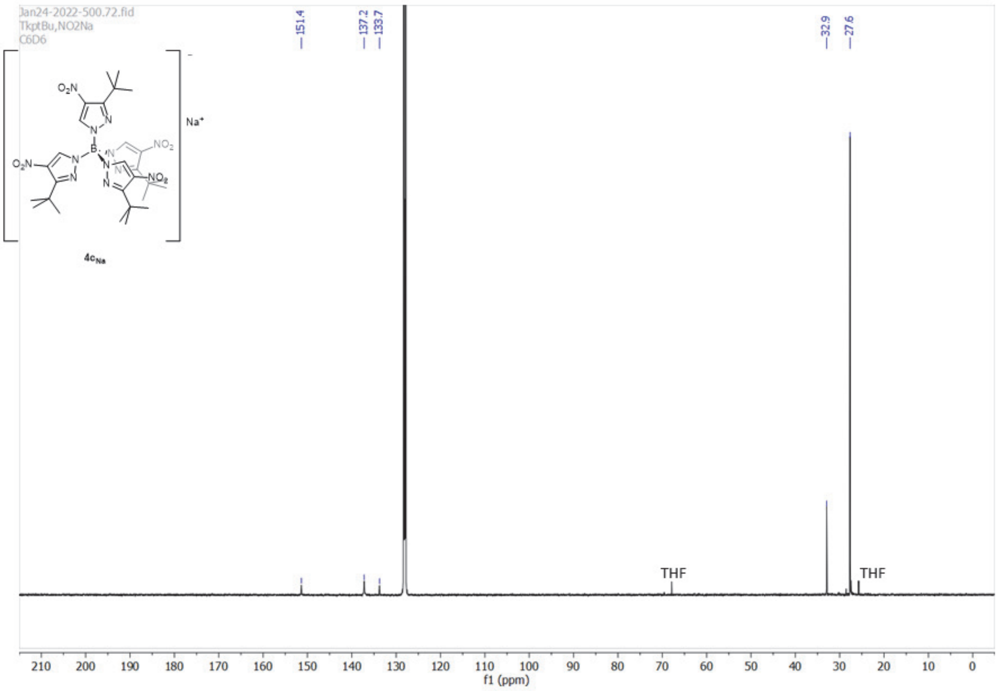

IR (ATR) spectrum of **$4c_{Na}$**

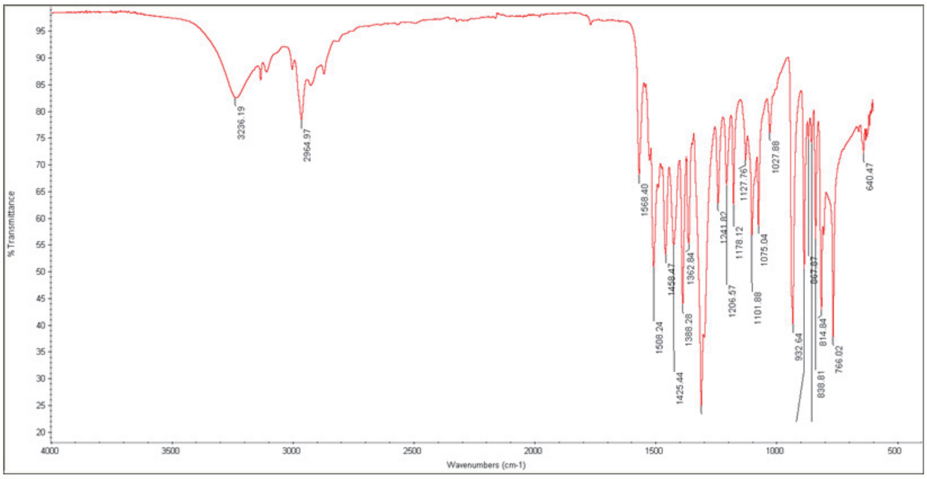

^{1}H ($CDCl_3$, 300 MHz) and ^{11}B ($CDCl_3$, 160 MHz) NMR of **$4f_{Na}$·1/3Hex**

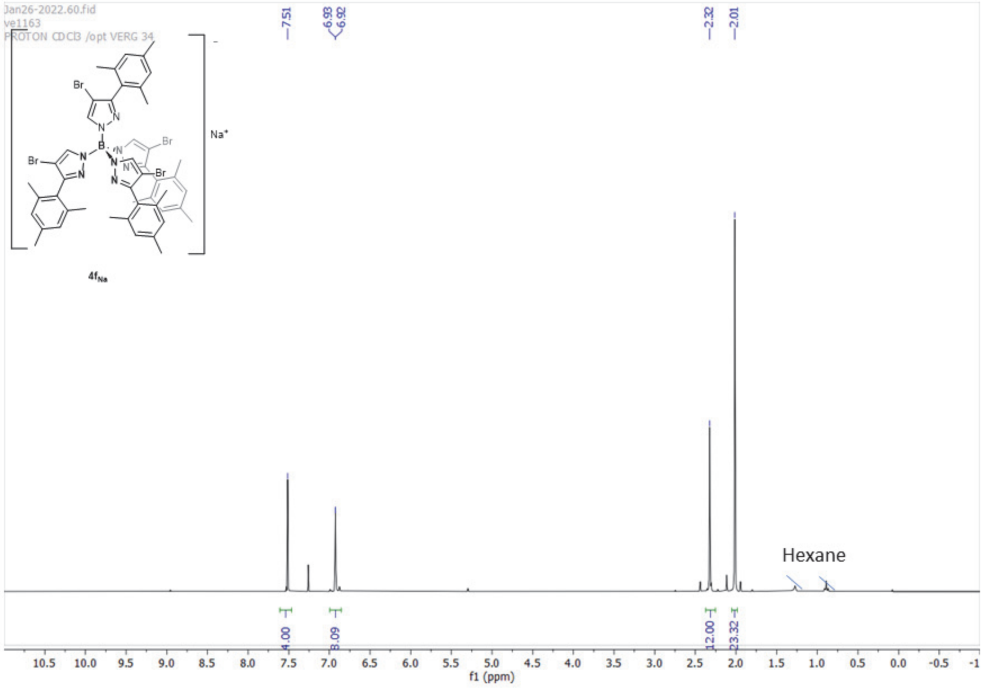

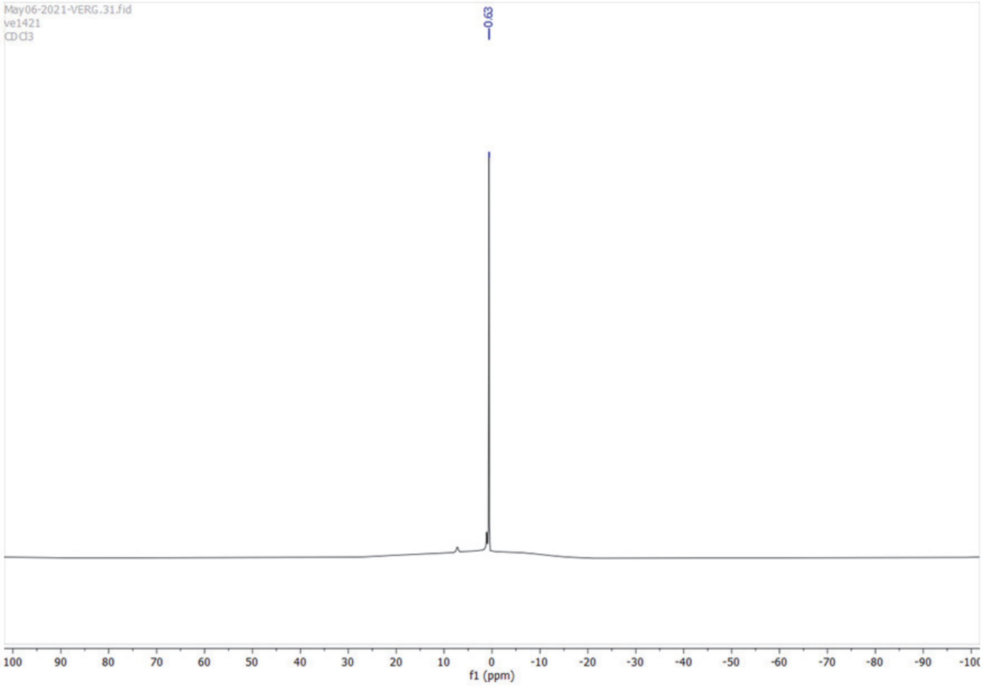

$^{13}C\{^1H\}$ ($CDCl_3$, 75 MHz) NMR of **$4f_{Na}$ ·1/3Hex**

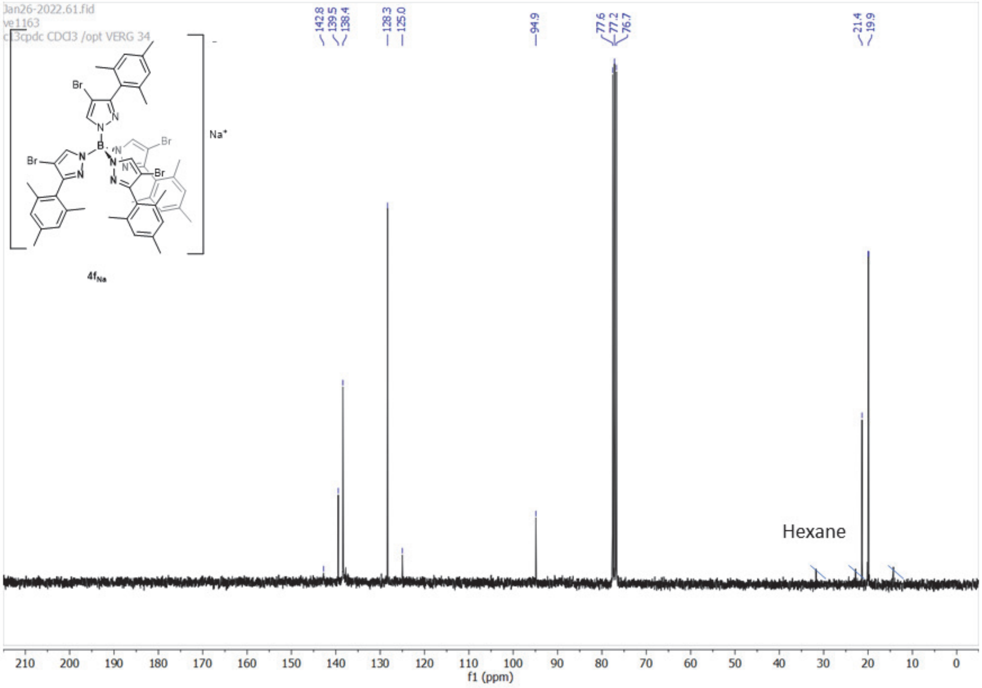

HRMS (+ESI) of **$4f_{Na}$**:

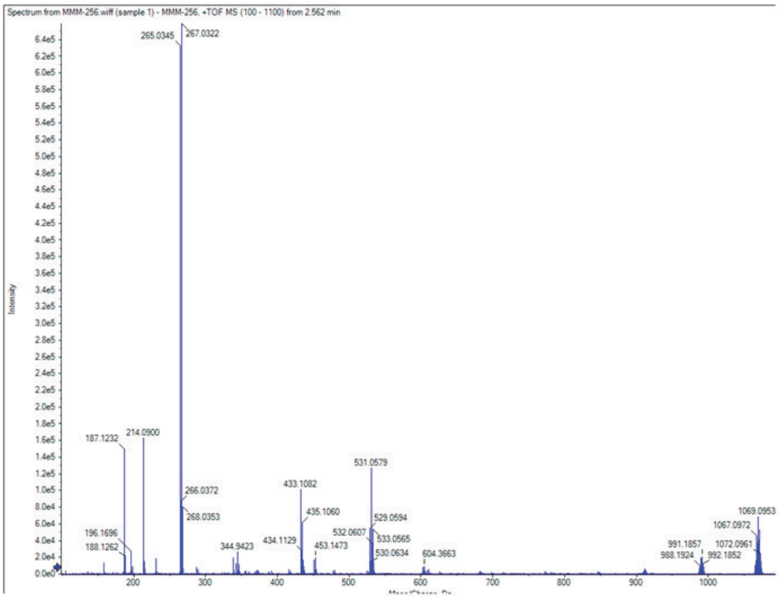

^{1}H (CD_2Cl_2, 500 MHz) and ^{11}B (CD_2Cl_2, 160 MHz) NMR of **$4f_{Tl}$**

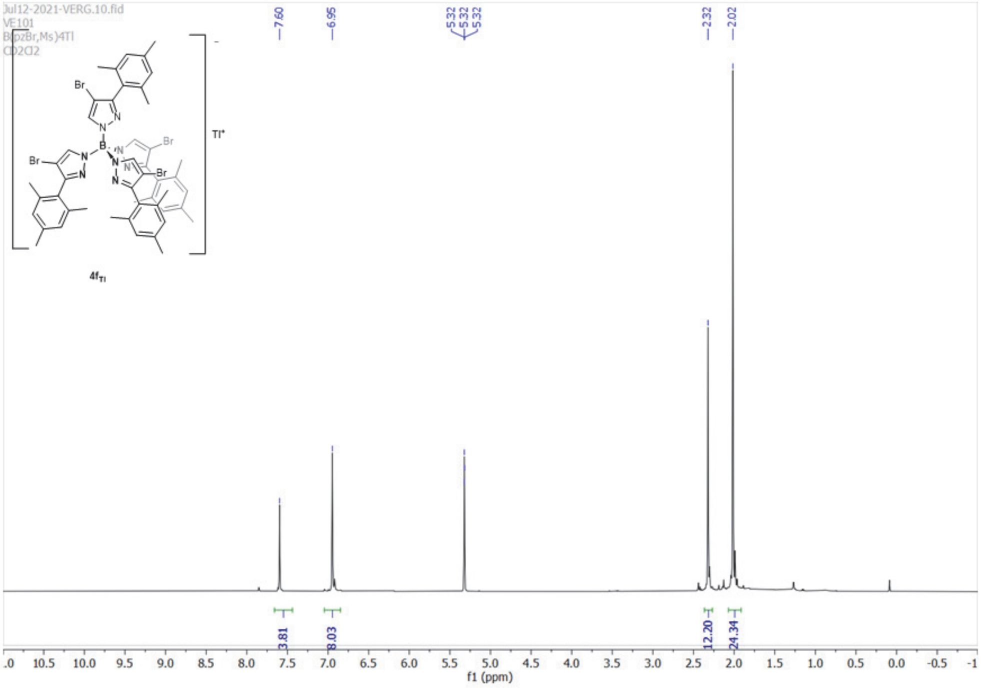

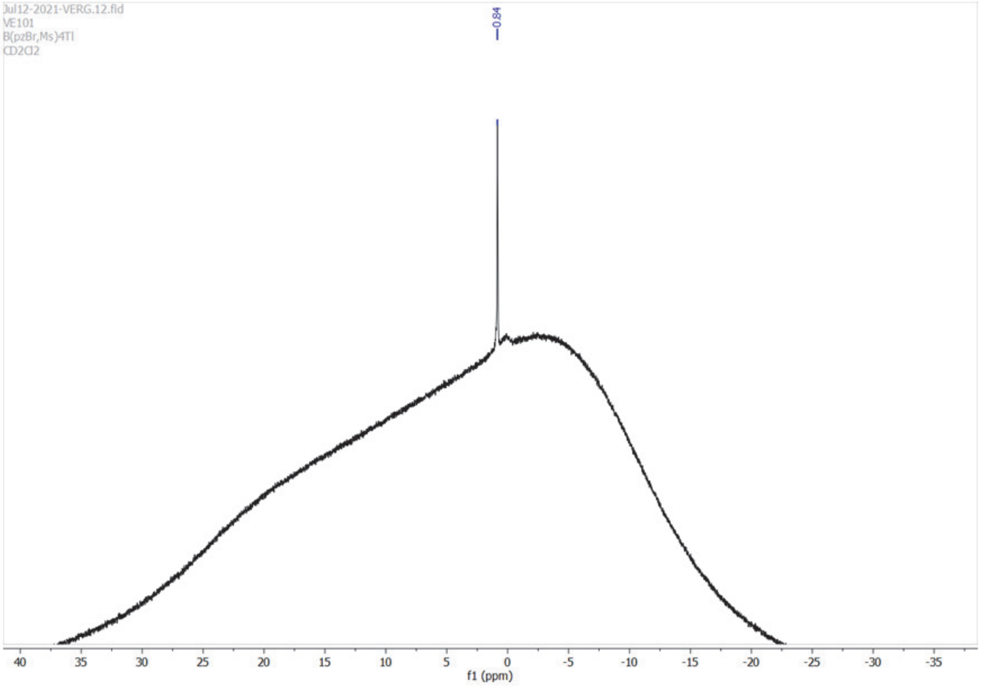

$^{13}C\{^{1}H\}$ (CD_2Cl_2, 125 MHz) NMR of **$4f_{Tl}$**

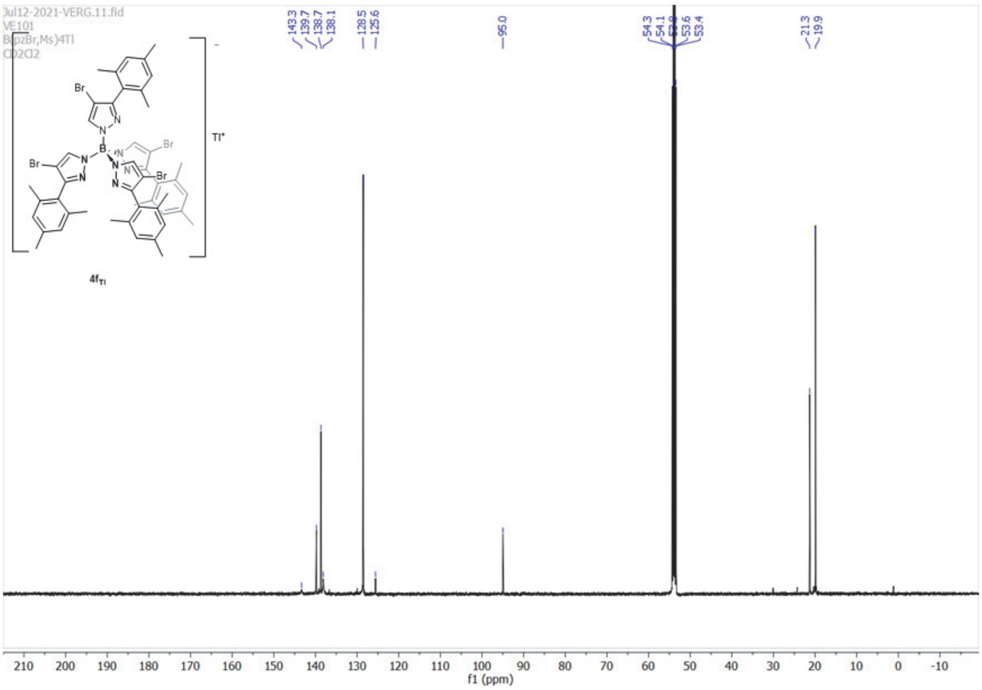

9. Single crystal X ray structure determination for $2c_{Tl}$, $3b_{Na}(OH_2)$, and $4f_{Tl}$

9.1 X-ray crystal structure of $\mathbf{2c_{Tl}}$

The molecular structure shows a symmetric Bp ligand **2c** chelated to the thallium atom. The thallium centre adopts an angular dicoordinated geometry with a N-Tl-N angle of 74.66(9)° (Figure S1). The six member metallacycle formed adopts a boat conformation with a Tl(1)···B(1) distance of 3.3006(48) Å. This metallacycle boat conformation is present in all eleven BpTl X-ray structures deposited in CCDC showing a Tl···B distance range of 3.12-3.83 Å.[14] The presence of a weak intramolecular [Tl···H-B] agostic interaction [Tl···H 2.67(5) Å and Tl···H-B 115(3)°] could favor the metallacycle boat conformation. This [Tl···H-B] interaction has been previously described and is present in all the reported BpTl structures, with Tl···H distance and Tl···H-B angle ranges of 2.25-3.58 Å and 97.0-127.6°, respectively (Table S2).

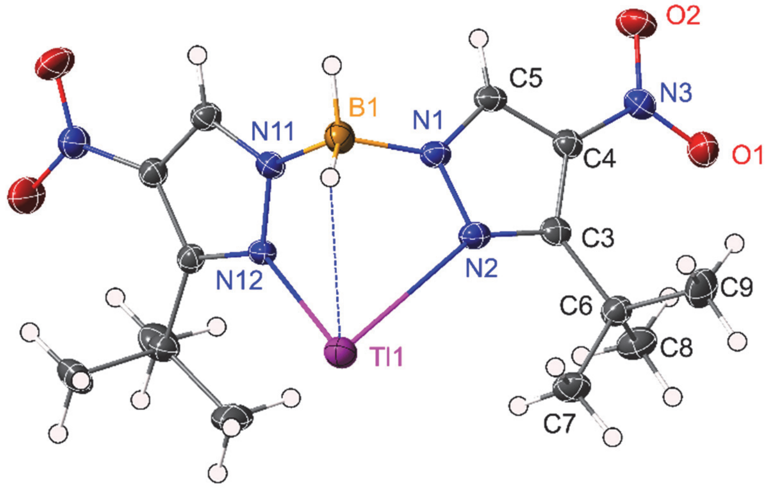

Figure S1. X-ray thermal ellipsoid plot of $\mathbf{2c_{Tl}}$ (50% probability level) with the labelling scheme. Selected bond lengths (Å) and angles (°): Tl(1)-N(12) 2.795(3), Tl(1)-N(2) 2.840(3), N(1)-N(2) 1.373(4), N(1)-B(1) 1.560(6), B(1)-N(11) 1.559(6), B(1)-H(1A) 1.11(4), B(1)-H(1B) 1.11(4), N(12)-Tl(1)-N(2) 74.66(9), N(2)-N(1)-B(1) 121.0(3), N(11)-B(1)-N(1) 110.9(3), H(1A)-B(1)-H(1B) 114(4), N(1)-N(2)-Tl(1) 106.4(2), Tl(1)···B(1) 3.3006(48), Tl(1)···H(1B) 2.675(51), Tl(1)···H(1B)-B(1) 115.1(34).

Crystal data for compound $\mathbf{2c_{Tl}}$: $C_{14}H_{22}BN_6O_4Tl$, M = 553.55, monoclinic, $P_{21/c}$, a = 13.3644(4), b = 9.7649(3), c = 14.9313(4) Å, β = 92.8290(10)°, V = 1946.19(10) Å^3, Z = 4, T = 120(2) K, λ = 0.71073 Å, Dcalc = 1.889 g/cm^3, μ = 8.330 cm^{-1}, 33523 reflections measured, 7680 unique (Rint = 0.0406), colourless prism of 0.18 x 0.14 x 0.12 mm size, crystal structure solved by dual space methods with all non-hydrogen atoms refined anisotropically on F^2 using the programs SHELXT-2018 and SHELXL-2019.[1,2] Hydrogen atoms were included using a riding model or as rigid methyl groups. GOF = 1.127, R (F_o, I > 2σ(I)) = 0.0345, Rw (F_o^2, all data) = 0.0877.

Table S2. Bond lengths (Å) and angles (°) for Tl···H-B interactions in $Bp^{x}Tl$ CCDC deposited structures.

Compound	Tl···B	Tl···H	Tl···H-B
$Bp^{tBu,Me}Tl$	3.118	2.439	112.81
$Bp^{tBu,iPr}Tl$	3.179	2.257	127.62
$Bp^{tBu,tBu}Tl$	3.209	2.573	114.60
$Bp^{Triptycyl}Tl$	3.313	2.701	111.21
BpTl	3.344	2.721	113.57
$Bp^{py}Tl$	3.433	3.024	102.08
$Bp^{Fc}Tl$	3.504	3.181	100.74
bis(indazolyl)Tl	3.519	3.157	108.89
$Bp^{bipy}Tl$	3.827	3.578	96.96

9.2 X-ray crystal structure of **$3b_{Na}(OH_2)$**

The molecular structure shows the sodium atom coordinated to a symmetric η^3-Tp ligand **3b** and a water molecule (Figure S2). The sodium centre adopts a distorted tetrahedral geometry. The molecule presents a plane of symmetry containing the O-Na···B axis and one of the pyrazol moieties [N(11)-C(15)]. The N-Na-N angles [N(2)-Na(1)-N(2)^i 82.35(10)°, N(2)-Na(1)-N(12) 84.43(7)°; [i = + x, + 0.5 - y, + z] are bigger than those found for other tetrahedral trispyrazolyl sodium complexes, that lie within the 75.75 – 80.96° range.[15] On the other hand, the O-Na-N angles [O(1)-Na(1)-N(2) 130.20(7)°, O(1)-Na(1)-N(12) 128.38(12)°] are within the 127.29-151.65° range found for tetrahedral trispyrazolyl sodium complexes. For all the described structures of this type of complexes, the N-Na-N angles are clearly closer than the N-Na-O ones, probably due to the bite requirements of the η^3-Tp ligand.

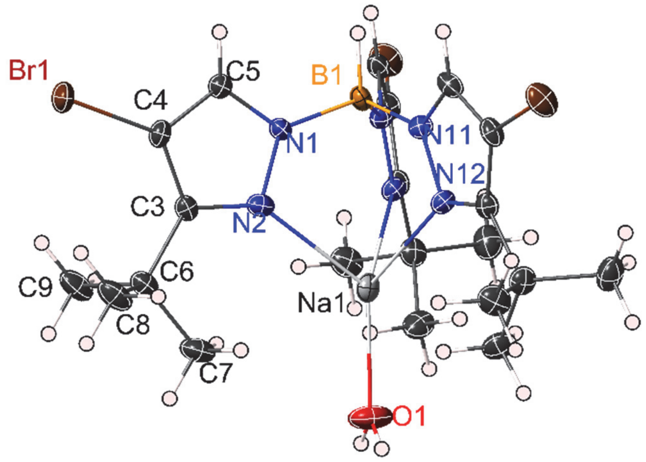

Figure S2. X-ray thermal ellipsoid plot of **$3b_{Na}(OH_2)$** (50% probability level) with the labelling scheme. Selected bond lengths (Å) and angles (°): Na(1)-O(1) 2.277(3),

Na(1)-N(2) 2.445(2), Na(1)-N(12) 2.452(3), B(1)-N(1) 1.540(3), B(1)-N(11) 1.551(4), N(1)-N(2) 1.373(2), N(11)-N(12) 1.376(4), O(1)-Na(1)-N(2) 130.20(7), N(2)-Na(1)-N(2)I 82.35(10), O(1)-Na(1)-N(12) 128.38(12), N(2)-Na(1)-N(12) 84.43(7), N(1)-B(1)-N(1)i113.4(3), N(1)-B(1)-N(11) 110.77(17), N(2)-N(1)-B(1) 124.6(2), N(12)-N(11)-B(1) 124.5(3).

Crystal data for compound **$3b_{Na}(OH_2)$**: $C_{21}H_{33}BBr_3N_6NaO$, M = 659.06, orthorhombic, Pnma, a = 11.3179(3), b = 16.9694(5), c = 14.6908(5) Å, V = 2821.49(15) Å^3, Z = 4, T = 120(2) K, λ = 0.71073 Å, D_{calc} = 1.552 g/cm^3, μ = 4.329 cm^{-1}, 30642 reflections measured, 4463 unique (Rint = 0.0727), colourless block of 0.12 x 0.10 x 0.02 mm size, crystal structure solved by dual space methods with all non-hydrogen atoms refined anisotropically on F^2 using the programs SHELXT-2018 and SHELXL-2019.[1,2] Hydrogen atoms were included using a riding model or as rigid methyl groups. GOF = 1.029, R (F_o, I > 2σ(I)) = 0.0632, Rw (F_o^2, all data) = 0.0718.

9.3 X-ray crystal structure of $\mathbf{4f_{Tl}}$ as a proof of composition

Very poorly diffracting crystals with high wR2 value and low bond precision on C-C bonds were obtained for compound $\mathbf{4f_{Tl}}$. All non-hydrogen atoms were refined with restrained U values (command RIGU). Local ring geometry of aromatic groups was also restrained (commands FLAT, SAME). GOF = 1.827, R1 (Fo, I > 2σ(I)) = 0.1479, wR2 (Fo2, all data) = 0.3927. The molecular structure shows the thallium centre coordinated to a symmetric η^3-Tkp ligand **4f** adopting a trigonal pyramidal geometry (Figure S3). The structure shows a highly encumbered environment of **4f** ligand. Low quality of data prevents for good refinement results even though low temperature was employed for measurements. The structure could be considered a proof of composition and the coordination mode of the complex.

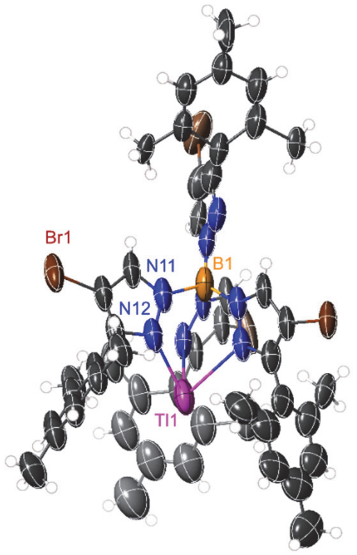

Figure S3. X-ray thermal ellipsoid plot of $\mathbf{4f_{Tl}}$ (30% probability level) with the labelling scheme.

Crystal data for compound **4f$_{Tl}$**: $C_{48}H_{48}BBr_4N_8Tl$, M = 1271.76, triclinic, P$\bar{1}$, a = 11.8894(17), b = 12.561(2), c = 16.881(3) Å, V = 2442.5(7) Å^3, Z = 2, T = 120(2) K, λ = 0.71073 Å, Dcalc = 1.729 g/cm3, μ = 6.620 cm-1, 51328 reflections measured, 8273 unique (Rint = 0.1562), colourless tablet of 0.13 x 0.09 x 0.06 mm size, crystal structure solved by dual space methods with all non-hydrogen atoms refined anisotropically on F^2 using the programs SHELXT-2018 and SHELXL-2019.[15] Hydrogen atoms were included using a riding model or as rigid methyl groups. Mesityl group C(81)-C(89) is disorder over two sites and has been refined with 51/49 % site occupancies, restrained U_{ij} components and appropriate similarity restraints (commands ISOR, SAME). Badly-behaving mesityl groups could be the reason for very poorly diffracting crystals with high wR^2 value and low bond precision on C-C bonds. All non hydrogen atoms were refined with restrained U values (command RIGU). Local ring geometry of aromatic groups was also restrained (commands FLAT, SAME). GOF = 1.827, R1 (F_o, I > 2σ(I)) = 0.1479, wR^2 (F_o2, all data) = 0.3927.

CCDC 2217594-2217596 contain the supplementary crystallographic data for this paper. These data can be obtained free of charge from The Cambridge Crystallographic Data Centre via www.ccdc.cam.ac.uk/structures.

10. References

(S1) Sheldrick, G. M. SHELXT - Integrated space-group and crystal-structure determination. *Acta Cryst.* **2015,** *A71*, 3-5. DOI: 10.1107/S2053273314026370.

(S2) Sheldrick, G. M. Crystal structure refinement with SHELXL. *Acta Cryst.* **2015,** *C71*, 3-5. DOI: 10.1107/S2053229614024218.

(S3) Trofimenko, S.; Calabrese, J. C.; Thompson, J. S. Novel polypyrazolylborate ligands: coordination control through 3-substituents of the pyrazole ring *Inorg. Chem.* **1987**, *26*, 1507-1514. DOI: 10.1021/ic00257a010.

(S4) Jain, R.; Mathur, M.; Lan, J.; Costales, A.; Atallah, G.; Ramurthy, S.; Subramanian, S.; Setti, L.; Feucht, P.; Warne, B.; Doyle, L.; Basham, S.; Jefferson, A. B.; Lindvall, M.; Appleton, B. A.; Shafer, C. M. Discovery of Potent and Selective RSK Inhibitors as Biological Probes. *J. Med. Chem.* **2015**, *58,* 6766-6783. DOI: 10.1021/acs.jmedchem.5b00450.

(S5) Yang, G.; Baran, P.; Martínez, A. R.; Raptis, R. G. Substituent Effects on the Supramolecular Aggregation of Ag^{I}-Pyrazolato Trimers. *Cryst. Growth Des.* **2013**, *13*, 264-269. DOI: 10.1021/cg301411j.

(S6) Janssen, J. W. A. M.; J. Koeners, H.; Kruse, C. G.; Habraken, C. L. Pyrazoles. XII. Preparation of 3(5)-nitropyrazoles by thermal rearrangement of N-nitropyrazoles. *J. Org. Chem.,* **1973**, *38*, 1777-1782. DOI: 10.1021/jo00950a001.

(S7) Rheingold, A. L.; White, C. B.; Trofimenko, S. Hydrotris(3-mesitylpyrazol-1-yl)borate and hydrobis(3-mesitylpyrazol-1-yl)(5-mesitylpyrazol-1-yl)borate: symmetric and asymmetric ligands with rotationally restricted aryl substituents *Inorg. Chem.* **1993**, *32*, 3471-3477. DOI: 10.1021/ic00068a015.

(S8) Baraldi, P. G.; Cacciari, B.; Spalluto, G.; Romangnoli, R.; Braccioli, G. Zaid, A. N.; Pineda de las Infantas, M. J. A New Synthetic Approach to Indazole Synthesis. *Synthesis*, **1997**, 1140-1142. DOI: 10.1055/s-1997-1328.

(S9) Lebedev, A. V.; Lebedeve, A. B.; Sheludyakov, V. D.; Kovaleva, E. A.; Ustinova, O. L.; Kozhevnikov, I. B. Synthesis of 3-Substituted Arylpyrazole-4-carboxylic Acids. *Russ. J. Gen. Chem.* **2005**, *75*, 782-789. DOI: 10.1007/s11176-005-0318-7.

(S10) Tang, Y.; Huang, W.; Kumar Chinmam, A.; Singh, J.; Staples, R. J.; Shreeve, J. M. Energetic Tricyclic Polynitropyrazole and Its Salts: Proton-Locking Effect of Guanidium Cations. *Inorg. Chem.* **2021**, *60*, 8339-8345. DOI: 10.1021/acs.inorgchem.1c01202.

(S11) Saito, M.; Ueda, T.; Tani, T.; Nakamura, K. US7393982B2, **2008**.

(S12) Ochando, L. E.; Amigó, J. M.; Rius, J.; Louër, D.; Fontenas, Ch.; Elguero. J. The crystal structure of 3,5-diisopropyl-4-nitropyrazole from X-ray powder diffraction data. *J. Molec. Structure*, **2001**, *562*, 11-17. DOI: 10.1016/S0022-2860(00)00766-3.

(S13) a) Trofimenko, S. Boron-pyrazzole chemistry. I. Prrazaboles. *J. Am. Chem. Soc.* **1967**, *89*, 3165-3170. DOI: 10.1021/ja00989a016; b) Trofimenko, S. Boron-pyrazole chemistry. III. Chemistry of pyrazaboles. *J. Am. Chem. Soc.* **1967**, *89*, 4948-4952. DOI: 10.1021/ja00995a021.

(S14) a) Mann, K. L. V.; Jeffery, J. C.; McCleverty, J. A.; Ward, M. D. Synthesis of the new ligand bis[3-(2-pyrazinyl-pyrazol-1-yl) dihydroborate, and the crystal structures of its complexes with thallium(I) and lead(II). *Polyhedron*, **1999**, *18*, 721-727. DOI: 10.1016/S0277-5387(98)00345-3; b) Sirianni, E. R.; Yap, G. P. A.; Theopold, K. H.

Ferrocenyl-Substituted Tris(pyrazolyl)borates—A New Ligand Type Combining Redox Activity with Resistance to Hydrogen Atom Abstraction. *Inorg. Chem.* **2014**, *53*, 9424-9430. DOI: 10.1021/ic5015658; c) Ghosh, P.; Hascall, T.; Dowling, C.; Parkin, G. Asymmetric bis(pyrazolyl)hydroborato ligands via direct synthesis: structural characterization of thallium and zinc complexes. *J. Chem. Soc. Dalton Trans.* **1998**, 3355-3358. DOI: 10.1039/A806298F; d) Fillebeen, T.; Hascall, T.; Parkin, G. Bis- and Tris(pyrazolyl)hydroborato Ligands with Bulky Triptycyl Substituents: The Synthesis and Structural Characterization of Tl[Bp^{Trip}] and Tl[Tp^{Trip}]. *Inorg. Chem.* **1997**, *36*, 3787-3790. DOI: 10.1021/ic970259r; e) Dowling, C.; Ghosh, P.; Parkin, G. Structural characterization of bis(pyrazolyl)hydroborato thallium complexes: monomeric "two-coordinate" thallium derivatives supplemented by [Tl…H-B] interactions. *Polyhedron* **1997**, *16*, 3469-3473. DOI: 10.1016/S0277-5387(97)00120-4; f) Davies, G. M.; Jeffery, J. C.; Ward, M. D. Chiral coordination polymers based on thallium(I) complexes of new bis- and tris(pyrazolyl)borate ligands with externally-directed 4-pyridyl groups. *New J. Chem.* **2003**, *27*, 1550-1553. DOI: 10.1039/B306659B; g) Ghosh, P.; Rheingold, A. L.; Parkin, G. Synthesis and Molecular Structure of Bis(pyrazolyl)hydroborato Thallium {[Bp]Tl}$_2$: A [$Bp^{RR'}$]Tl Complex with an Unbridged Close $Tl^{I}\cdots Tl^{I}$ Contact. *Inorg. Chem.* **1999**, *38*, 5464-5467. DOI: 10.1021/ic990647o; h) Craven, E.; Mutlu, E.; Lundberg, D.; Temizdemir, S.; Dechert, S.; Brombacher, H.; Janiak, C. Thallium(I) complexes with modified poly(pyrazolyl)borate ligands—metal-ligand coordination and crystal packing. *Polyhedron* **2002**, *21*, 553-562. DOI: 10.1016/S0277-5387(01)01026-9.

(S15) Dias, H. V. R.; Goh, T. K. H. H. Fluorinated tris(pyrazolyl)borates. Syntheses and characterization of sodium and copper complexes of $[HB(3\text{-}(CF_3),5\text{-}(Ph)Pz)_3]^-$ *Polyhedron* **2004**, *23*, 273-282. DOI: 10.1016/j.poly.2003.11.016.
